# The Mobilome; A Major Contributor to *Escherichia coli stx2*-Positive O26:H11 Strains Intra-Serotype Diversity

**DOI:** 10.3389/fmicb.2017.01625

**Published:** 2017-09-06

**Authors:** Sabine Delannoy, Patricia Mariani-Kurkdjian, Hattie E. Webb, Stephane Bonacorsi, Patrick Fach

**Affiliations:** ^1^Université Paris-Est, ANSES, Food Safety Laboratory, Platform IdentyPath Maisons-Alfort, France; ^2^Assistance Publique Hopitaux de Paris, Hôpital Robert-Debré, Service de Microbiologie, CNR Associé Escherichia coli Paris, France; ^3^Infection, Antimicrobials, Modelling, Evolution, UMR 1137, Institut National de la Santé et de la Recherche Médicale Paris, France; ^4^Infection, Antimicrobials, Modelling, Evolution, UMR 1137, Univ Paris Diderot, Sorbonne Paris Cité Paris, France; ^5^Department of Animal and Food Sciences, Texas Tech University Lubbock, TX, United States

**Keywords:** *Escherichia coli*, *E. coli*, STEC, *stx2*, Shiga toxin-producing *E. coli*, mobile genetic elements, comparative genomics, phylogenetic relationship

## Abstract

Shiga toxin-producing *Escherichia coli* of serotype O26:H11/H- constitute a diverse group of strains and several clones with distinct genetic characteristics have been identified and characterized. Whole genome sequencing was performed using Illumina and PacBio technologies on eight *stx2*-positive O26:H11 strains circulating in France. Comparative analyses of the whole genome of the *stx2*-positive O26:H11 strains indicate that several clones of EHEC O26:H11 are co-circulating in France. Phylogenetic analysis of the French strains together with *stx2*-positive and *stx*-negative *E. coli* O26:H11 genomes obtained from Genbank indicates the existence of four clonal complexes (SNP-CCs) separated in two distinct lineages, one of which comprises the “new French clone” (SNP-CC1) that appears genetically closely related to *stx*-negative attaching and effacing *E. coli* (AEEC) strains. Interestingly, the whole genome SNP (wgSNP) phylogeny is summarized in the *cas* gene phylogeny, and a simple qPCR assay targeting the CRISPR array specific to SNP-CC1 (SP_O26-E) can distinguish between the two main lineages. The PacBio sequencing allowed a detailed analysis of the mobile genetic elements (MGEs) of the strains. Numerous MGEs were identified in each strain, including a large number of prophages and up to four large plasmids, representing overall 8.7–19.8% of the total genome size. Analysis of the prophage pool of the strains shows a considerable diversity with a complex history of recombination. Each clonal complex (SNP-CC) is characterized by a unique set of plasmids and phages, including *stx*-prophages, suggesting evolution through separate acquisition events. Overall, the MGEs appear to play a major role in O26:H11 intra-serotype clonal diversification.

## Introduction

Shiga toxin-producing *Escherichia coli* (STEC) of serotype O26:H11/H- have been recognized for several years as public health threats. Indeed, they constitute the second most frequent serotype associated with clinical *E. coli* cases worldwide (Brooks et al., 2005; Mellmann et al., 2008; Vally et al., 2012; Marejkova et al., 2013; Byrne et al., 2014; EFSA and ECDC, 2014). STEC O26 are a diverse group of strains, and several clones of STEC O26 with distinct genetic characteristics have been identified and characterized (Zhang et al., 2000; Bielaszewska et al., 2013; Delannoy et al., 2015a).

One clone of STEC O26:H11 that is frequently isolated from food and associated with mild diseases and sporadic outbreaks traditionally harbors the Shiga toxin gene *stx1* alone or in combination with *stx2*. Previous studies have also determined that strains of this O26:H11 clone belong to sequence type 21 (ST21), are associated with allelic type 14 of the *arcA* gene, possess the *espK* gene, and react with the CRISPR-specific qPCR assays SP_O26-C and/or –D (Miko et al., 2010; Bugarel et al., 2011; Delannoy et al., 2012). In addition, this clone possesses the plasmid gene combination *ehxA*+ / *katP*+ / *espP*+ / *etpD*− (Zhang et al., 2000; Bielaszewska et al., 2013; Delannoy et al., 2015a).

Since the mid-1990s, a new highly pathogenic STEC O26:H11 clone —herein referred to as the “new European clone”— has been described and is commonly associated with hemolytic uremic syndrome (HUS; Zhang et al., 2000; Bielaszewska et al., 2013). Since its first description in Germany, the “new European clone” appears to have disseminated throughout Europe and has more recently emerged on the American and Asian continents (Brooks et al., 2005; Rivas et al., 2006; Zweifel et al., 2013; Trees et al., 2014; Januszkiewicz et al., 2015; Ishijima et al., 2017). The “new European clone” is characterized by belonging to ST29, the presence of the Shiga toxin *stx2a* gene only, and possesses the plasmid gene combination *ehxA*+ / *katP*− / *espP*− / *etpD*+ (Bielaszewska et al., 2013).

Recently we studied O26:H11 strains only harboring *stx2* isolated from pediatric patients with HUS in France (Delannoy et al., 2015a). In this study, some of the strains possessed the aforementioned genetic characteristics of the “new European clone,” confirming that it is established in France. However, some of the ST29 strains were negative for the plasmid and chromosomal genetic markers previously associated with this clone. Furthermore, several strains had an *stx2d* subtype. To our knowledge, this was the first description of *E. coli* O26:H11 carrying *eae* and *stx2d* isolated from human samples. These strains shared related CRISPR arrays with the presence of a large transposon within the first spacer of CRISPR2a. In response, a new CRISPR-specific qPCR, SP_O26-E, was designed to detect this “new French clone.”

The existence of these various clones demonstrates the continuing evolution of enterohemorrhagic *E. coli* (EHEC) O26:H11 and several studies have started to investigate the phylogenetic relationships of O26:H11 strains (Bletz et al., 2013; Ison et al., 2015a; Norman et al., 2015). Using whole genome sequences from ten EHEC O26:H11, Bletz et al. (2013) established an evolutionary model of STEC O26 based on 48 SNPs spread across the genome. This model defines four STEC O26 groups, or phylogenetically meaningful clonal complexes (SNP-CCs), with different genotypic and clinical characteristics. Bletz et al. (2013) hypothesized that EHEC O26 sequentially diverged from SNP-CC1 to SNP-CC4 from a common ancestor. According to this model the “new European clone” belongs to SNP-CC2, while the aforementioned ST21 *stx1*-positive strains belong to the newer SNP-CC3 and SNP-CC4. Using a similar approach, Norman et al. (2015) analyzed 180 STEC and non-STEC O26 strains to identify phylogenetically informative SNPs. The resulting set of SNPs shared only three common SNPs with that of Bletz et al. (2013). Interestingly, the 64 SNPs identified by Norman et al. (2015) clustered the *stx2*-positive strains with the non-STEC strains. This suggests that the *stx2*-positive strains were more closely related to the *stx*-negative attaching and effacing *E. coli* (AEEC) strains than to the *stx1*-positive STEC strains. Coincidentally, Ison et al. (2015b) recently showed that the CRISPR-specific qPCR assay SP_O26-E was found positive in most AEEC O26 strains isolated from US cattle they studied. Together, these data support a close evolutionary proximity between the AEEC O26 strains and the new *stx2*-positive strains. Incidentally, using the SNPs typing scheme proposed by Bletz et al. (2013), the American AEEC cattle strains clustered in the same clonal complex as the *stx2*-positive sequence type 29 (ST29) human O26:H11 strains demonstrating the close phylogenetic relatedness of these strains (Ison et al., 2015a).

In order to gain a better insight into the phylogenetic relationships of the various *stx2*-positive O26:H11 strains circulating in France, we have sequenced the whole genome of eight representative strains isolated in France (Delannoy et al., 2015b) using both Illumina and PacBio technologies. In the present study, a combination of bioinformatics methodologies was used to perform a comparative and phylogenetic analysis of these strains.

## Materials and methods

### Strains analyzed

A total of 18 *E. coli* strains have been used in this study (Table 1). Eight strains were sequenced by us (Delannoy et al., 2015b). In addition, 10 genomes were obtained from publicly available databases. These included the *stx1*-positive O26:H11 reference strain 11368, and six *stx*-negative O26:H11 strains isolated from humans and for which the CRISPR sequences were previously analyzed. Five of these had CRISPR arrays related to the CRISPR array of the “new French clone” (Yin et al., 2013; DEC9A, DEC9B, DEC9C, DEC9D, and DEC9E) and one isolated in France in 1952 (DEC10D) with a CRISPR array related to the reference EHEC O26:H11 strain (Hazen et al., 2012). Three human O26:H11 *stx2a* positive strains isolated in Norway (FHI4, FHI24, and FHI27) were also included (Haugum et al., 2014).

**Table 1:** Genetic characteristics of the *E. coli* O26:H11 strains included in this study.

**Sample**	***Stx* subtype**	**MLST**	**SNP-CC**	**SP_O26-E**	**Country of isolation (year)**	**Symptoms**	**Accession number**	**References**
36084	*2a*	ST21	CC3	−	France (2013)	HUS	LDXI00000000	Delannoy et al., 2015b
36079	*2a*	ST21	CC3	−	France (2013)	HUS	LDXH00000000	This study
36708	*2a*	ST29	CC2	−	France (2013)	HUS	LDXG00000000	Delannoy et al., 2015b
34827	*2a*	ST29	CC1	+	France (2012)	HUS	LDXF00000000	Delannoy et al., 2015b
34870	*2a*	ST29	CC1	+	France (2012)	HUS	LDXE00000000	Delannoy et al., 2015b
36348	*2d*	ST29	CC1	+	France (2013)	HUS	LDXD00000000	Delannoy et al., 2015b
36293	*2d*	ST29	CC1	+	France (2013)	HUS	LDXC00000000	Delannoy et al., 2015b
36493	*2d*	ST29	CC1	+	France (2013)	HUS	LDXB00000000	Delannoy et al., 2015b
DEC10D	−	ST21	CC3	−^#^	France (1952)	Diarrhea	NZ_AIGS00000000.1	Hazen et al., 2012
DEC9A	−	ST29	CC1	+^#^	USA (1961)	Diarrhea	NZ_AIGK00000000.1	Hazen et al., 2012
DEC9B	−	ST29	CC1	+^#^	USA (1979)	Diarrhea	NZ_AIGL00000000.1	Hazen et al., 2012
DEC9C	−	ST29	CC1	+^#^	Switzerland (1952)	Diarrhea	NZ_AIGM00000000.1	Hazen et al., 2012
DEC9D	−	ST29	CC1	+^#^	Denmark (1967)	Diarrhea	NZ_AIGN00000000.1	Hazen et al., 2012
DEC9E	−	ST29	CC1	+^#^	Mexico (1986)	Diarrhea	NZ_AIGO00000000.1	Hazen et al., 2012
FHI4	*2a*	ST21	CC3	−^#^	Norway (2002)	HUS	GCF_000951835.1	Haugum et al., 2014
FHI24	*2a*	ST29	CC2	−^#^	Norway (2007)	HUS	GCA_000936225.1	Haugum et al., 2014
FHI27	*2a*	ST29	CC2	−^#^	Norway (2008)	HUS	GCA_000951875.1	Haugum et al., 2014
11368	*1a*	ST21	CC4	−^#^	Japan (2001)	Diarrhea	NC_013361.1	Ogura et al., 2009

*The genome sequenced extracted from Genbank were tested in silico for the SP_O26_E marker (indicated by ^#^), while the other strains were verified by qPCR*.

### Whole genome sequencing, assembly, and annotation

Library preparation, Illumina sequencing and assembly of the eight French isolates were previously described (Delannoy et al., 2015b). In addition, in order to improve these draft sequences the same strains were sequenced in this study using a PacBio RSII system (Pacific Biosciences, Menlo Park, CA) at the GenoToul GetPlaGe sequencing core facility. The read count obtained during SMRT sequencing varied between 94,740 and 199,425 reads/sample, resulting in a 170- to 261-fold coverage of the genomes. Error correction and *de novo* assembly were performed using the CLC Genomics Workbench version 8.0.2 with the Genome finishing plug-in. Illumina short reads were mapped on the contigs generated and the consensus sequence was extracted. In the regions of low coverage (equal to or below five), “N” ambiguity symbols were inserted, except for strain 36079 for which these regions were filled from the PacBio generated *de novo* assembly sequence. Conflicts were solved by votes, including the quality score of the reads. Ambiguity symbols (i.e., Ns) made up <0.2% of the resulting sequences. Unless otherwise stated within the text these PacBio/Illumina hybrid assemblies were used for the analysis of the French isolates.

Annotations of all 17 draft genomes, including the eight French and three Norwegian isolate genomes were performed using PROKKA (Seemann, 2014) on the Aries galaxy platform (https://aries.iss.it/).

### Comparative genomic analysis

To evaluate the conservation of genetic material of the French strains compared to the reference strain 11368, the reads obtained by Illumina sequencing after trimming and quality filtering (Delannoy et al., 2015b) were mapped on strain 11368 genome using the CLC Genomics workbench version 7.5.1.

The Gegenees software version 2.1 (Agren et al., 2012) was used to perform phylogenomic analyses on the assembled genomes to evaluate the overall nucleotide conservation of the whole genome. A fragment length of 200 bp, a step-size of 100 bp was used and a threshold of 30%. The average normalized blastn scores of all fragment comparisons above the threshold were used as a measurement of overall genomic similarity and are shown in a heat-plot. The phylip file was exported from Gegenees and used in Mega 6 to build a dendrogram using the Neighbor-joining method.

Additionally, the MAUVE (version 2.3.1) Move Contigs tool was used to reorder the contigs obtained with the PacBio/Illumina hybrid assemblies to match the genomic arrangement of the O26:H11 reference strain 11368 finished genome. The best alignment was then chosen based on the highest weight score, an indicator of whether the predicted rearrangement exists, and the lowest number of locally collinear blocks (LCBs). All tiled genomes were then aligned against the reference *E. coli* O26:H11 11368 finished genome using progressive Mauve.

### Multi locus sequence typing

The sequence types (ST) of DEC9A, DEC9B, DEC9C, DEC9D, DEC9E, DEC10D, FHI4, FHI24, and FHI27 were determined from whole genome sequence data on the Center for Genomic Epidemiology (Scott et al., 2009) website (https://cge.cbs.dtu.dk/services/MLST/), using the Achtman *E. coli* MLST scheme (Wirth et al., 2006). The sequence types of the French strains as determined using the Achtman *E. coli* MLST scheme have already been published (Delannoy et al., 2015a).

### SNP typing of the isolates

The SNPs described by Bletz et al. (2013) were extracted from the whole genome data of all strains. The SNPs were then concatenated and Splitstree 4.12.6 was used to build a minimum spanning tree (Huson and Bryant, 2006). We used the SNPs genotypes of the 10 representative strains described by Bletz et al. (2013) to guide the construction of the tree and determine the relationship of the strains to the previously described clonal complexes (SNP-CCs). The same process was applied for the SNPs described by Norman et al. (2015).

### Whole genome SNP analysis

The phylogenetic relationship of the strains was assessed by whole-genome SNP (wgSNP) analysis using CSI Phylogeny 1.2 on the CGE server (https://cge.cbs.dtu.dk/services/CSIPhylogeny/; Kaas et al., 2014). The SNP alignment generated was treated in MEGA version 6.06 and in Splitstree 4.12.6 to generate a Maximum likelihood tree or a minimum spanning tree, respectively.

### CRISPR analysis

Analysis of CRISPR sequences was performed by using the CRISPy Python Script (2.7.6) developed in-house. This script uses a method developed by Yin et al. (2013) to assign allele numbers for each strain. Briefly, each unique spacer and repeat are recorded in separate databases associated with a number and a letter, respectively. Each unique spacer combination within a CRISPR locus defines a CRISPR allele, the listing of which is also contained in a database.

### *Cas* genes analysis

The naming of *cas* genes and their classification into *Cas* array subtypes were done according to Makarova et al. (2011). Nucleotide sequences of all *cas* genes of each isolate were extracted in Artemis 16.0.0 (Rutherford et al., 2000) from the PROKKA annotated sequences. When all eight *cas* genes were found, the concatenated sequence of all ORFs were aligned in BioEdit 7.1.3.0 (Hall, 1999). The alignment generated was then used to create a maximum likelihood tree in Mega 6.

### *Stx* gene analysis

Nucleotide sequence of the *stx2* gene of each isolate was extracted in Artemis 16.0.0 from the PROKKA annotated sequences. The full nucleotide sequences, including both A and B subunits sequences and the short intergenic sequence, of the *stx2* genes were aligned with sequences of reference *stx2a* and *stx2d* genes from Scheutz et al. (2012) in CLC sequence viewer 7.0.2. The alignment generated was then used to create a tree in Mega 6 using the maximum likelihood method with the Jukes Cantor correction and using a circular representation.

### Prophages prediction and *Stx* prophage comparison

The contigs containing the *stx* gene of all isolates were identified after annotation and / or Blastn analysis. They were aligned with CLC Genomics Workbench (version 8.0.2). The percentage of nucleotide identity (similarity matrix) and number of SNPs (difference count matrix) were calculated with BioEdit 7.1.3.0.

In order to obtain prophage sequences ordered along the genome, the fasta files generated after Mauve rearrangement (see above) were concatenated and prophages were predicted using the PHASTER (Arndt et al., 2016) server (http://phaster.ca/). This application outputs a fasta file containing all predicted prophage regions (the “phageome”) for each isolate. The global “phageome” of all strains was compared using the Gegenees software (see above). The predicted prophage regions were then pairwise compared using the progressive Mauve alignment tool, EasyFig (Sullivan et al., 2011) and Blastn.

### Plasmids analysis

A database containing all complete plasmid sequences from *E. coli* (*n* = 226) was downloaded from NCBI. A local blastn was performed using contigs from each genome as a query against the plasmid database to identify contigs matching known plasmid sequences. Each contig producing an alignment corresponding to >50% of its length was further checked by Blastn against the non-redundant nucleotide (nr/nt) database. When necessary, the closest identified plasmid was used to identify and reorder contigs with blastn and Mauve, respectively.

Replicons were identified with the Plasmid Finder 1.3 tool and antimicrobial resistance genes were identified with the ResFinder 2.1 application, both on the CGE website (https://cge.cbs.dtu.dk/services/PlasmidFinder/; https://cge.cbs.dtu.dk/services/ResFinder/).

## Results and discussion

### Overview

Based on a previous analysis of *E. coli* O26:H11 French isolates carrying the *stx2* gene only (Delannoy et al., 2015a), we sequenced eight strains representing the different genetic profiles identified using both an Illumina MiSeq (Delannoy et al., 2015b) and a PacBio RS II (Pacific Biosciences). The eight new *E. coli* O26:H11 genomes were compared to each other as well as to previously reported O26:H11 genomes derived from patients with diarrhea and HUS, including: the O26:H11 reference strain 11368, a set of strains (FHI4, FHI24, FHI27) isolated from HUS patients in Norway and carrying the *stx2a* gene only (Haugum et al., 2014), and a set of *stx*-negative strains (DEC9A, DEC9B, DEC9C, DEC9D, DEC9E and DEC10D) isolated from diarrheagenic patients and selected according to their CRISPR profiles (Hazen et al., 2012).

Assembly of the reads generated by the PacBio produced between 23 and 41 contigs with an *N*_50_ between 424 and 785 kb (Supplementary Table 1), which are ~10-fold improvements compared to assemblies generated with the Illumina MiSeq short reads (Delannoy et al., 2015b). The average total genome size obtained with the PacBio sequencing was 5.8 Mbp. This is comparable to the total genome size (chromosome and plasmids) of 5.8 Mbp of the O26:H11 reference strain 11368. Although the PacBio library sizing at 10 kb eliminated all small plasmids, the smaller genome size obtained with the Illumina sequencing (5.4 Mbp in average) indicates a greater loss of genomic information in the assemblies generated with the short reads. The combination of Illumina and PacBio sequencing allowed us to obtain high quality sequences that can be used for comparative genomic analyses.

### Whole genome comparisons

We performed whole genome comparisons to examine the overall genetic relatedness of the isolates.

**Figure 1:**
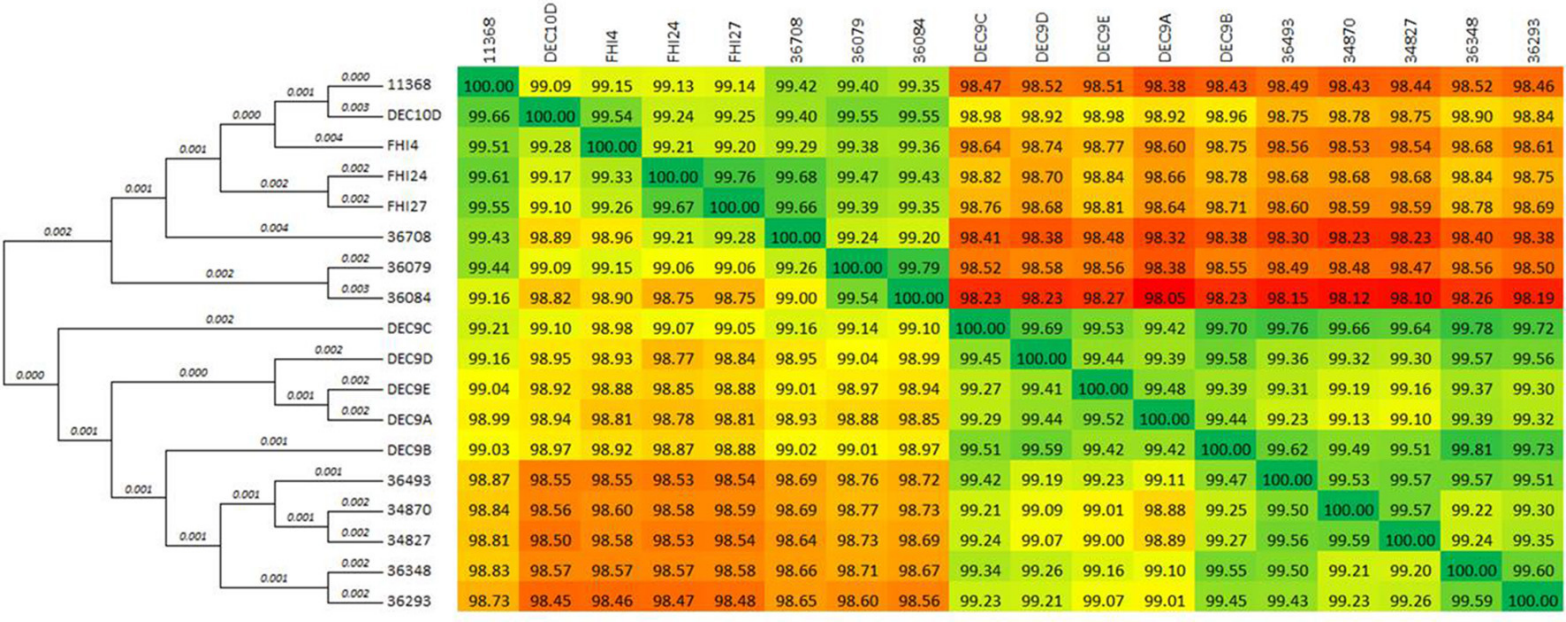
Phylogenetic overview of the O26:H11 isolates. The whole genomes of the 18 isolates were compared using Gegenees 2.1 and Mega 6.06. The heat-plot comparison matrix is based on a fragmented alignment using BLASTN (200/100) with a threshold set at 30%. The percentage similarities between the conserved regions of the genomes are indicated in the comparison matrix, where the colors vary from red (low similarity) to green (high similarity). The distance matrix was used to produce a dendrogram in Mega using the neighbor-joining method. The optimal tree with the sum of branch length = 0.05552887 is displayed (branch lengths are shown above the branches).

The comparison matrix of a fragmented alignment of all isolates generated with the software Gegenees showed that the genomes of the 18 strains exhibited extensive similarity, showing 98.1–99.79% overall nucleotide identity to each other (Figure 1). Isolates 36084 and 36079 appear to be more closely related to the reference genome 11368 than isolates 36348, 34870, and 34827.

A comparison of the genomes by multiple genome alignment in the program MAUVE showed that the genomic architecture of these strains is syntenic and share a conserved chromosomal backbone (Supplementary Figure 1). Additionally, mapping the short reads of the French isolates to the reference strain 11368 genome sequence indicated a high degree of conservation across the chromosomal backbones of all isolates. In fact, 92–98% of the reference genome was conserved in all isolates (Supplementary Table 2). Among the French isolates, strains 36084, 36079, and 36708 appeared to have more genetic material in common with reference strain 11368; specifically, 96–98% of the reference strain genome was conserved, with 4–5% of the reads representing extra genetic material. Strains 34827, 34870, 36348, 36293, and 36493 have conserved 92–93% of the reference strain genome with 10–18% of the reads representing extra genetic material. Most of the divergence appears to be concentrated in mobile genetic elements (MGEs) such as prophages, integrated elements, and plasmids (Supplementary Figure 2). Very few ORFs located on the chromosome backbone of reference strain 11368, excluding prophages and integrative elements, are absent from the French isolates. Those that have known functions include genes involved in adhesion properties and host adaptation and survival. Briefly, the genes *frmR* and *frmA* of the *frmRAB* operon (loci ECO26_0391, ECO26_0392 and ECO26_0393 in AP010953), involved in the degradation of formaldehyde, a by-product of lignin degradation (Herring and Blattner, 2004), are absent in strain 36493. Some of the genes (ECO26_2103, ECO26_2104, ECO26_5529) involved in type I fimbriae synthesis (Pusz et al., 2014) are absent from strains 36493, 34870, 34827, 36293, and 36348. Additionally, the fimbrial genes *fimA* (ECO26_5511) and *fimH* (ECO26_5517) in strains 36493, 34870, 34827, 36293, and 36348 are more similar to the corresponding genes from O111 reference strain 11128 (99–100% nucleotide identity vs. 96–98% nucleotide identity with O26:H11 strain 11368). Furthermore, the *symE* gene (ECO26_5539), involved in oxidative stress response (Kawano et al., 2007; Barbagallo et al., 2011; Campilongo et al., 2014), and several restriction endonucleases (ECO26_5540, ECO26_5541, ECO26_5542) involved in defense mechanisms were absent from strains 36493, 34870, 34827, 36293, and 36348. The *speG* gene (ECO26_2286), also involved in oxidative stress response (Kawano et al., 2007; Barbagallo et al., 2011; Campilongo et al., 2014), was absent from strains 34827 and 34870. These differences suggest that some isolates may have slightly different reservoirs or ecological niches.

### Genotyping

#### MLST

The sequence type of the eight French strains was determined previously (Delannoy et al., 2015a). The sequence types of DEC9A, DEC9B, DEC9C, DEC9D, DEC9E, DEC10D, FHI4, FHI24, and FHI27 were determined from whole genome sequence data. DEC10D and FHI4, like 36084 and 36079, belonged to ST21 while all other strains belonged to ST29 (Table 1). Overall, all the strains belonged to two STs, both of which are from the same ST29 complex (they differ by one allele).

#### SNP typing of the isolates

**Figure 2:**
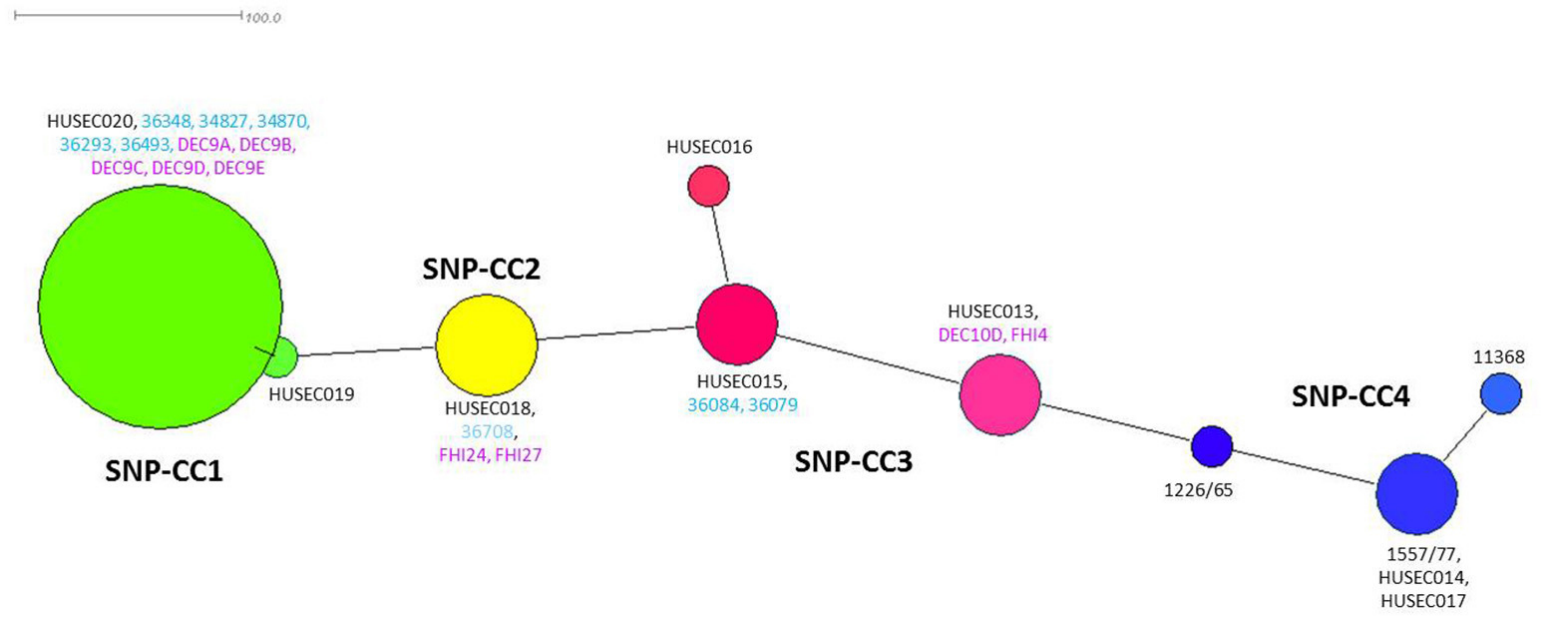
Minimum spanning tree based on 48 SNPs (Bletz et al., 2013). Each node represents a unique SNP profile. The node size is proportional to the number of isolates. The four clonal complexes (SNP-CCs) are represented by different colors: SNP-CC1 is shown in green, SNP-CC2 in yellow, SNP-CC3 in red and SNP-CC4 in blue. This figure was created with Splitstree (version 4.12.6). Representative strains for each SNP-CC and sequenced by Bletz et al. (2013) are indicated in black, French strains sequenced in this study are indicated in light blue and strains extracted from published WGS projects are indicated in purple.

Bletz et al. (2013) previously developed a set of 48 SNPs that could be used to classify EHEC O26 in clonal complexes representative of phylogenetically conserved groups. SNP genotyping of the French isolates using this set of 48 SNPs resulted in three unique profiles (Table 1). Their phylogenic relationships are displayed in a minimum spanning tree together with HUSEC isolates using data from Bletz et al. (2013) as well as data from the *stx*-negative DEC strains and the *stx2a*-positive human strains isolated in Norway and extracted from published WGS projects (Figure 2). Overall, the French ST29 strains spread between SNP-CC1 and SNP-CC2, with the *stx2a* and *stx2d* SP_O26-E-positive strains clustered in SNP-CC1 (“new French clone”), and the *stx2a* SP_O26-E-negative strains clustered in SNP-CC2 (“new European clone”). The ST21 strains clustered in SNP-CC3 (“classic” EHEC O26:H11). The *stx*-negative strains did not form separate clusters and the “new French clone” clusters with the non-STEC strains, confirming data obtained with American cattle and clinical isolates (Ison et al., 2015a). The same cluster organization appears when the SNPs were analyzed using the Neighbor-joining method (Supplementary Figure 3).

In a recent study Norman et al. (2015) identified another set of 43 SNPs that can be used to infer O26:H11 strains relationships. Contrary to Bletz et al. (2013), Norman et al. (2015) included *stx*-negative strains in their data set. When using this set of SNPs, the strains displayed a similar cluster organization, although the two SNP sets had only three common SNPs. It appeared that SNP-CC3 isolates clustered together, SNP-CC2 isolates clustered separately, and all SNP-CC1 isolates clustered along with the non-STEC O26:H11 strains (Supplementary Figure 4). Similarly, when looking at a set of three SNPs described by Norman et al. (2012) to differentiate STEC from non-STEC strains all the isolates from SNP-CC1 appeared classified as non-STEC (Supplementary Table 3).

#### Whole genome SNPs

**Figure 3:**
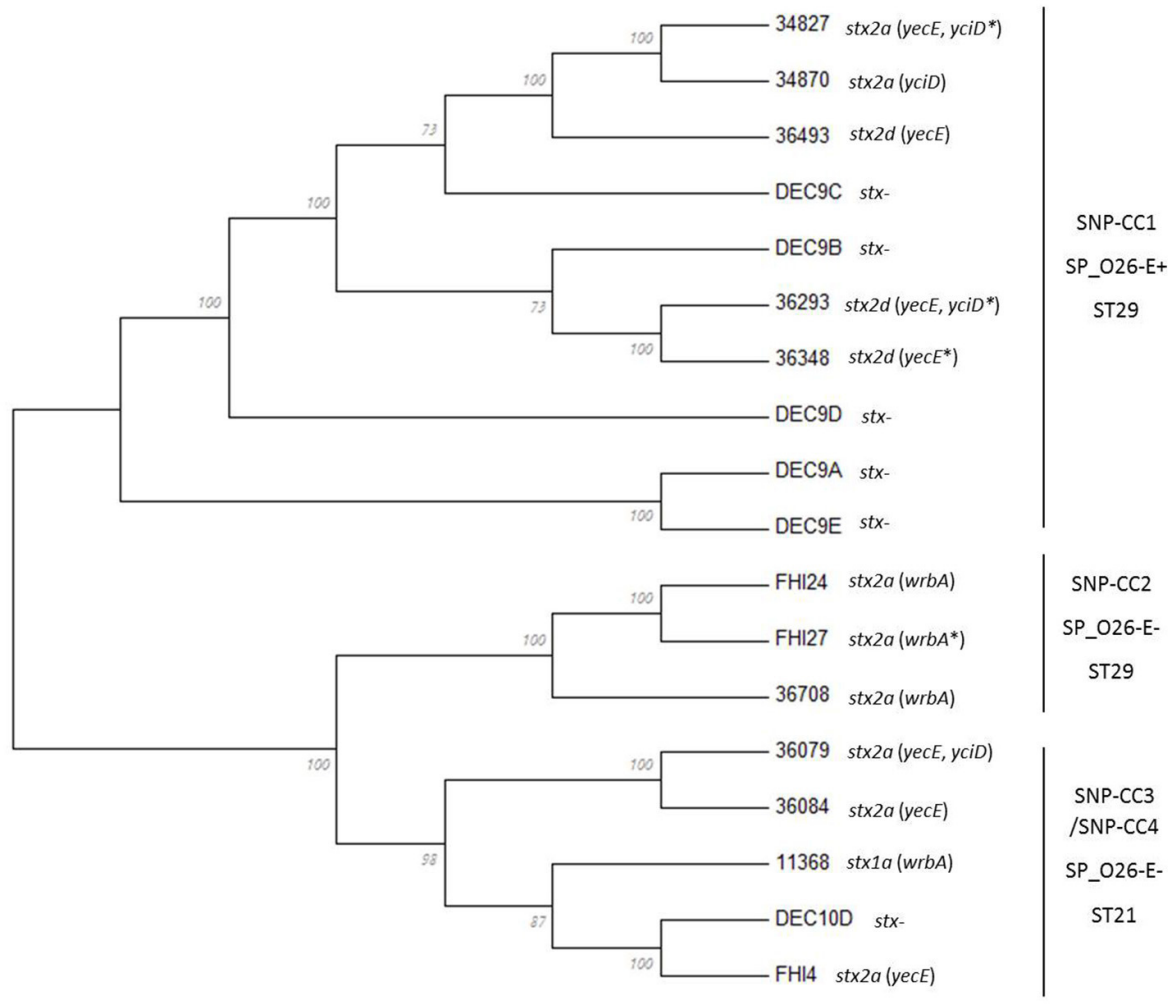
Phylogenetic relationships of O26:H11 strains. The phylogenetic relationships of the 18 strains were assessed by whole-genome SNPs (wgSNP) analysis using CSI Phylogeny 1.2 on the CGE server. The SNPs alignment generated was imported and analyzed in Mega6. The evolutionary history was inferred using the Maximum Likelihood method based on the Jukes-Cantor model. The bootstrap consensus tree inferred from 100 replicates is displayed. Branches corresponding to partitions reproduced in <50% bootstrap replicates are collapsed. The percentage of replicate trees in which the associated taxa clustered together in the bootstrap test (100 replicates) is shown next to the branches. Initial tree(s) for the heuristic search were obtained automatically by applying Neighbor-Join and BioNJ algorithms to a matrix of pairwise distances estimated using the Maximum Composite Likelihood (MCL) approach, and then selecting the topology with superior log likelihood value. All positions containing gaps and missing data were eliminated. There were a total of 4,419 positions in the final dataset. The stx subtype and insertion site(s) of the *stx*-phage are indicated next to the isolate name. ^*^Indicates that the insertion site was inferred.

In order to get a finer classification of the strains, we extracted the whole genome SNPs (wgSNPs) of the whole set of strains using the CSI Phylogeny application with the sequence of the O26:H11 strain 11368 as reference. When comparing the wgSNPs of the whole set of strains the strains clustered in two well-supported lineages separated by their CRISPR array (Figure 3, see below): one lineage contained the SP_O26-E-negative strains, which include the ST21 strains (*stx*-positive and *stx*-negative) and the ST29 “new European clone” (SNP-CC3 and SNP-CC2 respectively). The ST29 *stx2*-positive “new French clone” and the *stx*-negative ST29 strains from SNP-CC1, all SP_O26-E-positive, were on a separate lineage. The same clusters were obtained when O26:H1 reference strain 11368 or *E. coli* K12 laboratory strain were used as reference, as well as when O111 strain 11128 was included as an outgroup or when only the *stx*-positive strains were used to extract the SNPs and construct a phylogenetic tree (not shown). The CRISPR-based marker SP_O26-E (together with *stx*) thus appears as a good choice to identify the “new O26:H11 French clone.”

All these SNP typing methods confirm a close phylogenetic proximity of the “new French clone” to the non-STEC or AEEC O26:H11 strains, suggesting a direct evolution of this clone from an AEEC clone with uptake of *stx*-phages (Zhang et al., 2000; Leomil et al., 2005; Bugarel et al., 2011; Bielaszewska et al., 2013).

### CRISPR array and *Cas* genes comparison

The CRISPR-Cas system is thought to prevent infection by foreign DNA such as plasmids and bacteriophages (Richter et al., 2012; Kiro et al., 2013). As such, it can be speculated that the CRISPR-*Cas* system of a strain has evolved with its “mobilome” and reflects to some extent the prophage and plasmid repertoire of such strain (Vale and Little, 2010).

We previously characterized the CRISPR arrays of all the French isolates (Delannoy et al., 2015a). Based on the whole genome data we analyzed the CRISPR arrays of DEC9A, DEC9B, DEC9C, DEC9D, DEC9E, DEC10D, FHI4, FHI24, and FHI27 (Supplementary Table 4). Among this set of strains the CRISPR1 loci displayed six unique alleles (35% allele diversity) and the CRISPR2a loci displayed nine unique alleles (53% allele diversity) arranged in twelve CRISPR types. More importantly, the DEC9s strains (DEC9A to DEC9E) possess the large transposon (IS3 family), or a variation thereof, within their CRISPR2a array and are predicted to be positive for the CRISPR SP_O26-E assay. DEC10D, FHI4, FHI24, and FHI27 are all predicted to be negative for the CRISPR SP_O26-E assay. All of the strains, that possessed the large transposon and were positive (or predicted to be positive) for the CRISPR SP_O26-E (Table 1), clustered together in SNP-CC1. Hence, as demonstrated in previous studies (Touchon et al., 2011) there is a global congruence between the CRISPR array (presence or absence of the large transposon) and the phylogenetic background.

**Figure 4:**
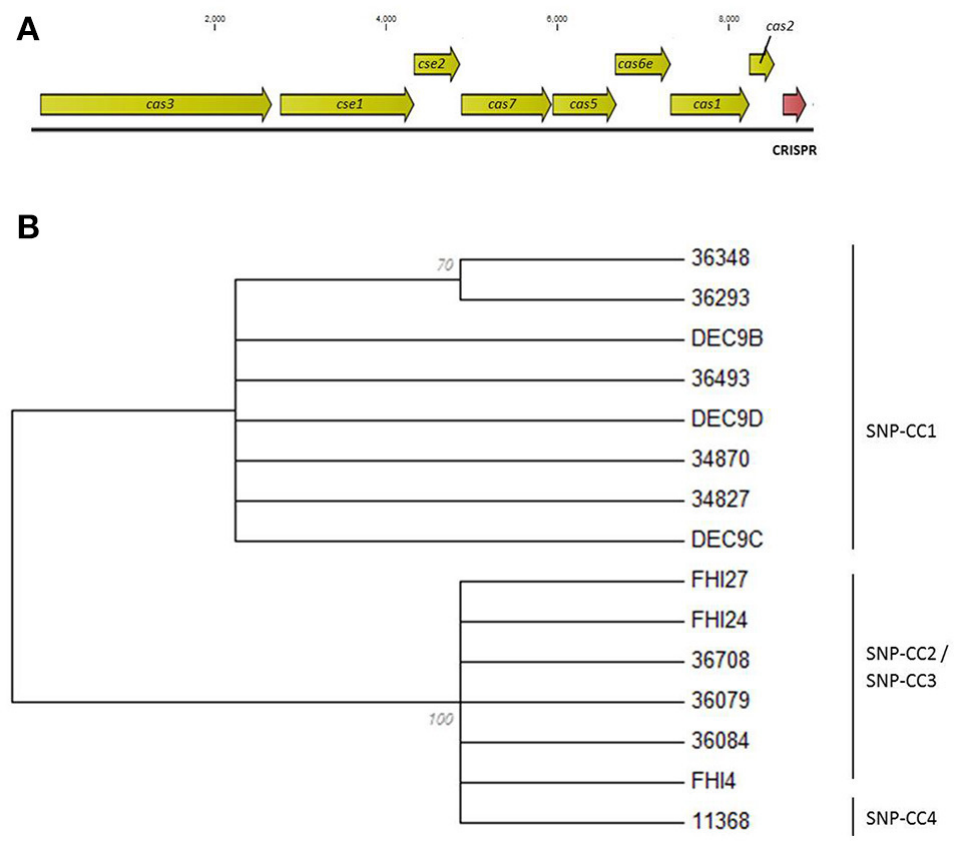
Evolutionary relationships of the O26:H11 strains based on the concatenated ORFs of the eight *cas* genes. **(A)** Schematic representation of the type I-E CRISPR-Cas system. The *cas* genes are drawn to scale. **(B)** Maximum Likelihood tree based on the concatenated ORFs of the eight *cas* genes. The evolutionary history was inferred by using the Maximum Likelihood method based on the Jukes-Cantor model. The bootstrap consensus tree inferred from 100 replicates is displayed. Branches corresponding to partitions reproduced in <50% bootstrap replicates are collapsed. The percentage of replicate trees in which the associated taxa clustered together in the bootstrap test (100 replicates) is shown next to the branches. Initial tree(s) for the heuristic search were obtained automatically by applying Neighbor-Join and BioNJ algorithms to a matrix of pairwise distances estimated using the Maximum Composite Likelihood (MCL) approach, and then selecting the topology with superior log likelihood value. All positions containing gaps and missing data were eliminated. There were a total of 8421 positions in the final dataset. Evolutionary analyses were conducted in MEGA6. Isolates DEC9A, DEC9E, and DEC10D were not included in the analysis due to the presence of frameshifts.

The CRISPR loci can confer immunity only in the presence of *cas* genes. The *cas* genes will thus most likely “evolve” with the CRISPR array and variation in the *cas* genes sequences should reflect variation in the CRISPR array. We therefore examined and compared the sequence of the *cas* genes of all isolates to evaluate if they segregated the isolates in the same clusters as the CRISPR arrays. All 18 strains possessed a type I-E *Cas* system with eight *cas* genes—*cas3, cse1, cse2, cse4*/*cas7, cas5e*/*cas5, cse3*/*cas6e, cas1, cas2* (Figure 4A). However, the *cas3* and *cse1* gene sequences of DEC9A both contain frameshifts generating premature stop codons. Similar frameshifts were observed in the *cas3* gene sequences of DEC9E and DEC10D. These frameshifts might be due to sequencing or assembly errors but this could not be bio-informatically determined. These were thus not included in the *cas* genes analysis. Such mutations would most likely cause the CRISPR-Cas system to be inactive. Based on a *cas* gene tree reconstructed from a concatenation of all eight *cas* genes, there are two general sequence profiles present across all strains (Figure 4B). Similar to what was observed with wgSNPs, one branch contains all the SNP-CC1, SP_O26-E-positive strains (34827, 36493, 34870, 36348, and 36293, as well as DEC9B, DEC9C, and DEC9D) while the other branch contains the SP_O26-E-negative strains from SNP-CC2, SNP-CC3 and SNP-CC4 (11368, 36708, 36084, 36079, FHI4, FHI24, and FHI27). The *cas* gene tree is congruent with respect to the CRISPR array organization (presence of the large transposon) and to some extent with the clonal complexes clustering or the wgSNP analysis. It is not however congruent with the MLST phylogeny (as ST29 is split between the two branches).

### *Stx2*-phage profiles

#### *Stx2* genes analysis

Analysis of the *stx2* gene sequences (Supplementary Figure 5) confirmed the subtype previously determined (Delannoy et al., 2015a). There are two different *stx2a* genes and a single *stx2d* gene amongst the strains (Supplementary Table 5). The *stx2a* genes from strains 36079 and 36084 (SNP-CC3, ST21) were identical to each other, but different from the *stx2a* gene of isolates 34870, 36708, and 34827. A blastn search of the *stx2a* sequence of 36084 indicates that it is a common *stx* gene among various serotypes including O26:H11, as well as O157:H7, O145:H28 and O111:H8 (Supplementary Table 6). The *stx2a* genes from strains 34870, and 34827 (SNP-CC1, ST29) were identical to each other and to the *stx2a* gene of strain 36708 (SNP-CC2, ST29). A blastn analysis of this *stx2a* gene sequence demonstrated that it is identical to that of O104:H4 strains that caused the 2011 STEC O104:H4 epidemic in Europe (Supplementary Table 6). It was also found in O157:H7 and O145:H28 strains. It was also identical to the *stx2a* gene found in the FHI4, FHI24, and FHI27 *stx2a*-positive O26:H11 Norwegian strains. It is particularly interesting that the same *stx2a* variant can be found in strains belonging to different lineages. The *stx2d* genes in strains 36293, 36348, and 36493 are identical to each other and to the O55:H7 strain 06-5231 (accession EF584538). A blastn analysis indicates that this gene sequence was rarely reported, with a single occurrence in the Genbank nr/nt database in O55:H7 strain 06-5231 (Supplementary Table 6). This particular O55:H7 strain was isolated together with a O177:NM strain from a child with HUS (Gilmour et al., 2007).

#### *Stx2*-converting prophages analysis

The long-read sequencing allowed us to obtain the full genome sequences of the Shiga toxin-converting prophages in strains 36708, 34870, 36493, as well as FHI24 (46–60 kb) and partial sequences ranging from 20 to 43 kb for the *stx*-prophages in the other isolates (Table 2). These partial sequences contained the parts of the prophages coding for the modules involved in infection and propagation, but were missing the structural proteins coding sequences. These data, including the partial sequences, were used to compare the structures of the *stx*-prophages between the various strains.

**Table 2:** Insertion sites and selected characteristics of the *stx2* prophages.

**Isolate**	**Contig**	**stx subtype**	**Insertion site**	**Completeness^§^**	**Size (kb)**
34827	Contig 2	*stx2a*	*yecE*	Incomplete (*attL*)	25
	Contig 17	*stx2a*	yciD^*^	Incomplete (*attL*)	34
34870	Contig 5	*stx2a*	*yciD*	Complete (*attL* + *attR*)	52
36079	Contig 1	*stx2a*	*yciD*	Incomplete (*attL*)	25
	Contig 4	*stx2a*	*yecE*	Incomplete (*attL*)	20
36084	Contig 14	*stx2a*	*yecE*	Incomplete (*attL*)	20
36293	Contig 10	*stx2d*	*yecE*	Incomplete (*attL*)	22
	Contig 13	*stx2d*	yciD^*^	Incomplete (*attL*)	22
36348	Contig 21	*stx2d*	yecE^*^	Incomplete^#^	43
36493	Contig 1	*stx2d*	*yecE*	Complete (*attL* + *attR*)	46
36708	Contig 6	*stx2a*	*wrbA*	Complete (*attL* + *attR*)	60
FHI24	Contig 37	*stx2a*	*wrbA*	Complete (*attL* + *attR*)	60
FHI27	Contig 8	*stx2a*	wrbA^*^	Incomplete	25
FHI4	Contig 38	*stx2a*	*yecE*	Incomplete (*attL*)	37

* *Indicates that the insertion site was inferred after scanning the known insertions sites yecE, wrbA, yehV, sbcB, Z2577, argW, prfC, and torST by in silico PCR*.

§ *When identified, the attL (integrase side) and attR (tail proteins side) sites are indicated between brackets. The sequence of the att sites at the yecE and yciD insertion sites was 5′-CAYGCAGTTAA-3′ and 5′-TTGAAACSAT-3′ at the wrbA insertion sites*.

# *Indicates that the prophage is presumed incomplete*.

The *stx*-phages generally insert their DNA into highly preferred single sites. Only a small number of integration sites have been described so far for *stx*-phages (Herold et al., 2004; Ogura et al., 2009; Steyert et al., 2012; Kruger and Lucchesi, 2015). It was previously suggested that the phage insertion site is specified by the phage integrase itself and not the host genome (Campbell et al., 2002; Steyert et al., 2012; Bobay et al., 2013). Hence, prophages with the same integrase should be inserted in the same insertion site, provided it is present and unoccupied in the host genome. We determined the *stx*-phage insertion sites for the different strains. When the genomic location could not be obtained with certainty, integrity of known phage insertion sites was scanned (Table 2).

##### SNP-CC1 Stx2a prophages

**Figure 5:**
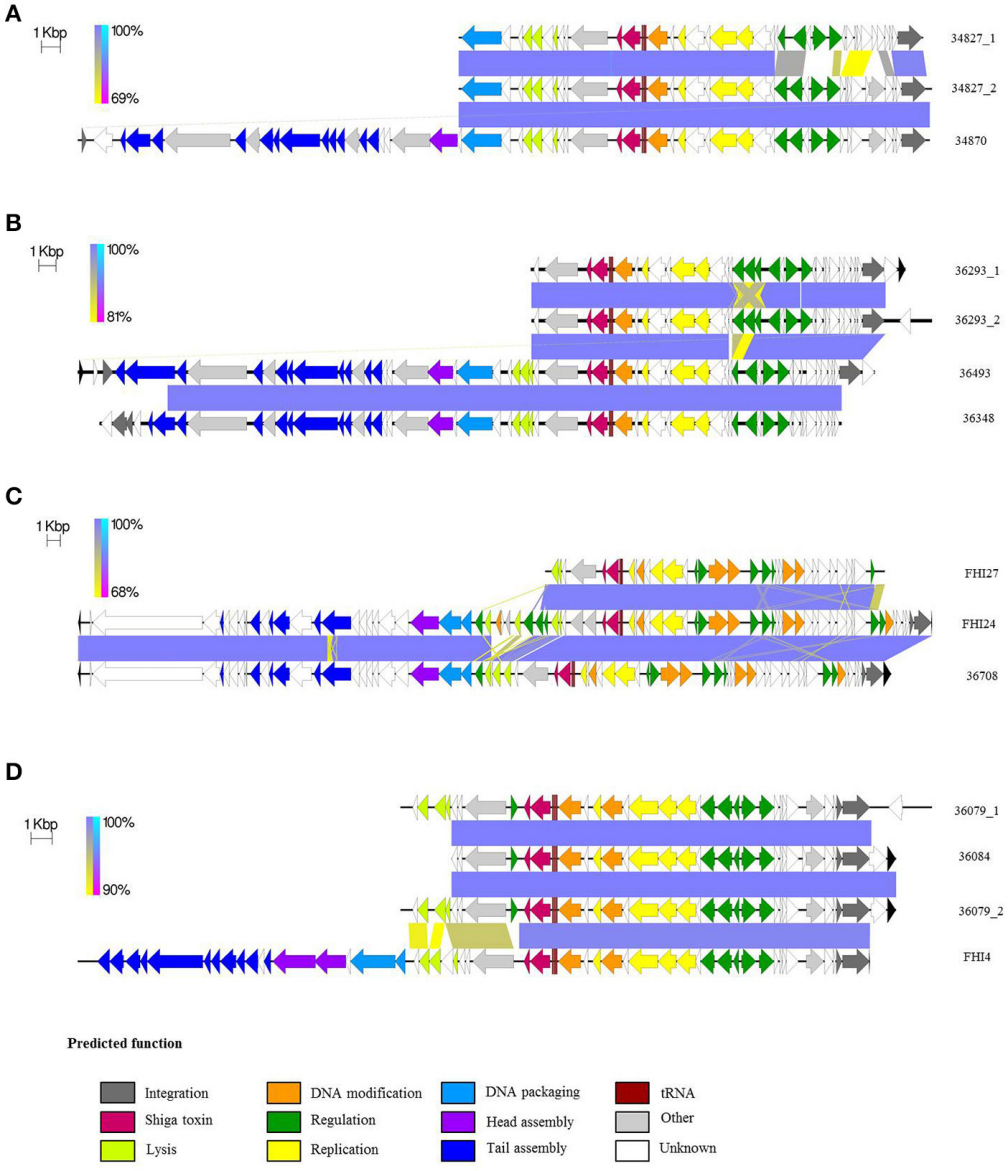
Comparative analysis of the various *stx*-prophages in the O26:H11/H- strains. **(A)** Comparison of SNP-CC1 *Stx2a*. **(B)** Comparison of SNP-CC1 *Stx2d* prophages. **(C)** Comparison of SNP-CC2 *Stx2a* prophages. **(D)** Comparison of SNP-CC3 *Stx2a* prophages. The sequences of the *stx*-prophages were compared with EasyFig. The homologous regions are connected. The color of the zone connecting the strains is related to the direct or reverse homology between the strains according to the scale present at the left of each comparison. The arrows depict ORFs identified with PROKKA. The direction of the arrow represents the transcription orientation. The ORFs are color-coded according to their predicted function. Black arrows indicate ORFs outside of the predicted prophages.

Among the *stx2a*-positive strains from SNP-CC1, strain 34870 was found to contain a single *stx*-phage, while strain 34827 was found to contain two *stx2a* prophages with identical *stx2a* genes. The structure of the full genome of the *stx2a* prophage in isolate 34870 and the partial genomes of *stx2a* prophages in isolate 34827 are shown in Figure 5A.

The same integrase was present in the *stx*-phage of isolates 34827 and 34870. The same integrase (3 SNPs) can be found in the EH297 prophage inserted in *yecE* in *E. coli* K12 strain (accession AJ431361). Thus, it could be hypothesized that these isolates with the same integrase have the *stx*-phage inserted in the *yecE* site. One of the *stx*-prophages of strain 34827 was indeed found to occupy the *yecE* site. For strain 34870, the *stx*-phage was however inserted in tandem with what appears to be an ancestor of prophage ECO26_P08 at the *yciD* site (Supplementary Figure 6). This could be the result of a recombination between the ECO26_P08 prophage ancestor and the *stx*-phage initially inserted at another site generating a chimeric structure. It could also represent a tandem integration of both phages. Such integration events have previously been observed with lambdoid phages, although not *stx*-phages (Ogura et al., 2009). Interestingly, while the ECO26_P08 prophage ancestor in strain 34870 appears fully functional with a complete set of genes, prophage ECO26_P08 lacks all virion structural proteins. Integration of *stx*-phages at the *yciD* site has rarely been described (Steyert et al., 2012), and never for O26:H11/H- strains. The second *stx*-prophage of strain 34827 also appears to be inserted with prophage P08 at the *yciD* site.

Although the *stx2a* genes are identical, the two partial *stx*-prophages in strain 34827 appear slightly different (86.1% nucleotide identity; Figure 5A and Supplementary Figure 7A). The prophage inserted at the *yciD* site presents 99.8% nucleotide identity to the corresponding part of the *stx*-prophage in isolate 34870 inserted at the same site (Supplementary Figure 7B). A blastn search of the homologous *stx2a*-prophage present in strains 34827 and 34870 returns a nearly exact match from O145 and O121 HUS strains (Supplementary Table 7). A blastn search of the second *stx2a*-prophage of strain 34827 in the nr/nt database, however, doesn't return any match (covering at least 80% of the query with 90% identity). The two partial *stx2a* prophages in strain 34827 share a common part and each has a divergent part. This divergence suggests mosaicism, i.e., recombination of one of the prophages with another prophage in the bacterial genome.

We could speculate that the original phage was inserted at the *yecE* site of the ancestor strain (as suggested by its integrase). This ancestor strain could have evolved with duplication of the prophage at the *yciD* site. Then, in one lineage, ancestor to strain 34827, the original prophage at the *yecE* location could have formed a mosaic structure with another prophage of the genome. In that new lineage the prophage at the *yecE* location has a unique structure and sequence, while the prophage at the *yciD* location remains similar to the original phage. In the other lineage, ancestor to strain 34870, the original prophage at the *yecE* location could have been lost and only the prophage at the *yciD* location would remain. The very fast phage pool turn-over in *E. coli* with frequent acquisition and loss (Bobay et al., 2013) supports the possibility of this evolution model.

##### SNP-CC1 Stx2d prophages

Among the *stx2d*-positive strains, strains 36493 and 36348 were found to contain a single *stx*-phage, while strain 36293 was found to contain two *stx2d* prophages with identical *stx2d* genes. The structure of the full genome of the *stx2d* prophage in isolate 36493 and the partial genomes of *stx2d* prophages in isolates 36348 and 36293 are shown in Figure 5B.

The same integrase, similar to that of SNP-CC1 *stx2a* prophages and *E. coli* K12 EH297 prophage (3 SNPs), is present in the *stx*-phages of all *stx2d* isolates. The *stx*-phage was found to occupy the *yecE* site in strains 36493. In isolate 36293 one of the *stx*-prophages was found to occupy the *yecE* site. The second *stx*-prophage appears to be inserted with prophage ECO26_P08 at the *yciD* site. The *wrbA, yehV*, sbcB, *Z2577, argW, prfC*, and *torST* sites were found intact in isolate 36348. The *yecE* site was found to be probably occupied. However, it is not clear from our data if the *stx*-prophage actually occupies this location.

The partial *stx2d* prophages in isolates 36293 and 36348 were very similar to the corresponding part in strain 36493 (Figure 5B). The two partial *stx*-prophages in isolate 36293 had 99.7% nucleotide identity over 20 kbp. The difference is due to a 60-nucleotide stretch of *N*s in one of the prophages (Supplementary Figure 8A). The *stx*-phages in strains 36348 and 36493, both inserted at the *yecE* site, present 99.9% nucleotide identity (31 SNPs difference) over 39 kbp (Supplementary Figure 8B), while the prophages in strain 36293 present 92.3–92.6% nucleotide identity with those in strains 36348 and 36493 respectively over 20 kbp (Supplementary Figure 8C). The difference between the prophages in strain 36293 and those in strains 36348 and 36493 is centered on a small region spanning 1–3 ORFs (Figure 5B). Blastn analyses of these *stx*-prophage sequences do not show any identical occurrence in the nr/nt database. The commonalities between the two prophages in isolate 36293 suggest a recent duplication of the phage (without accumulation of point mutations), rather than a double insertion, as the large size and diversity of the phage pool makes the probability of the double acquisition of the same phage very unlikely (Casjens, 2003).

##### SNP-CC2 Stx2a prophages

The three strains from SNP-CC2 (the “new European clone”) contained a single *stx2*-prophage each. The structure of the full genomes of the *stx2a* prophages in isolates 36708 and FHI24 as well as the partial genome of *stx2a* prophage in isolate FHI27 are shown in Figure 5C. The same (or highly similar) integrase was present in isolates 36708, FHI24, and FHI27. That integrase gene has 100% identity with the integrase from the O104:H4 epidemic strain (Accession HF572917 region: 1133565-1134875), in which the *stx*-phage is inserted in *wrbA*. Accordingly, the *stx*-prophage was found to occupy the *wrbA* site in strains 36708 and FHI24. For isolate FHI27, the insertion site also appeared to be *wrbA* as the *wrbA* site was found occupied while the *yehV, yecE, sbcB, Z2577, argW, prfC*, and *torST* sites were intact.

The *stx2a* prophages in strains 36708, FHI24, and FHI27 showed remarkable structure and sequence conservation across 20 kb (Figure 5C), and those in strains 36708 and FHI24 further showed conservation over the whole 60 kb length of the prophage, with some differences in the central lysis region. A blastn analysis of the *stx*-prophage in isolate 36708 showed that besides FHI24 and FHI27, it had 99% nucleotide sequence identity to the *stx*-prophage of O104:H4 strains from the 2011 epidemic (accession CP003289, HF572917). It is only distantly related to other *stx*-phages for example from O157:H7. Accordingly, a 99.7% nucleotide identity was found between the *stx*-prophage of strain 36708 and prophage P13374 (Accession HE664024) from an O104:H4 strain of the 2011 epidemic (Supplementary Figure 9; Beutin et al., 2012). The different central region of the *stx*-phage in isolate FHI24 could result from integration of phage P11374 in the chromosome followed by a subsequent mosaicism with other prophages integrated elsewhere within the bacterial genome.

##### SNP-CC3 Stx2a prophages

Among the *stx2a*-positive strains from SNP-CC3, 34084 and FHI4 were found to contain a single *stx*-phage while 36079 was found to contain two *stx2a* prophages with identical *stx2a* genes. Only partial genomes were recovered for these *stx*-prophages. The structure of the partial genome of *stx2a* prophages in isolates 36084, 36079, and FHI4 are shown in Figure 5D. The same (or highly similar) integrase is present in the *stx*-phage of isolates 36084, 36079, and FHI4 (1 SNP between 36084 and FHI4) and in phage EH297. The *stx*-phage was indeed found to occupy the *yecE* site in strains 36084 and FHI4. One of the *stx*-prophages of isolate 36079 was found to occupy the *yecE* site. The second *stx*-prophage appeared to be inserted with prophage ECO26_P08 at the *yciD* site. The two partial *stx*-prophages in strain 36079 appeared identical (99.9% nucleotide identity with only 1 SNP over 22 kb), most likely resulting from prophage duplication (Figure 5D and Supplementary Figure 10A). Similarly, the genetic structure and sequence of the partial *stx2a* prophages from strains 36084 and FHI4 were all highly similar to that of 36079 (Figure 5D and Supplementary Figure 10B). A blastn analysis of 36084 *stx*-prophage showed that it is highly similar to *stx2* phages of various O111:H- and O145:H28 strains (Supplementary Table 8).

#### Stx2-converting prophages overview

Overall, there are three types of *stx* bacteriophages in the strains studied. The modular genetic structures in the various strains are illustrated in Figure 5. Sequence comparisons demonstrate high sequence similarity of the predicted prophage regions among strains of the same SNP-CC. Indeed, high sequence similarity was found among all *stx2d* prophages, among *stx2a* prophages from SNP-CC1, among *stx2a* prophages from SNP-CC2, and among *stx2a* prophages from SNP-CC3, while *stx2a* and *stx2d* prophages from SNP-CC1 are more distantly related (Supplementary Figure 11). Although the sequence of the *stx2a* gene was identical between SNP-CC2 and SNP-CC1 the genetic architecture and nucleotide sequence of the predicted prophage of SNP-CC2 was significantly different from SNP-CC1 (Supplementary Figure 12) and also from SNP-CC3 (Supplementary Figure 13), the only similarity being the *stx* gene. The predicted *stx2a* prophage sequences from SNP-CC1 and SNP-CC3 have however identical genetic structures and high sequence identity (Supplementary Figure 14). This is particularly striking as SNP-CC1 and SNP-CC3 strains belong to different lineages. The different *stx2* prophages between the SNP-CCs strongly suggest separate acquisition events. The mixture of phage structures and *stx* genes between the strains of different phylogenetic background (or CCs) suggest a complex history of recombination. But overall, the *stx*-phage insertion site appears consistent with the integrase sequence. There were two different integrases in the strains studied. One of them appeared linked to the *wrbA* site. The second integrase appears preferentially linked with the *yecE* site, with *yciD* as a secondary insertion site or for double insertion events and/or duplication events.

It is particularly interesting to note that the “new European clone,” which was shown to be a virulent clone with a high HUS rate (Bielaszewska et al., 2013; Bletz et al., 2013), has the same *stx*-phage (including *stx2a* gene) as the O104:H4 strain responsible of the largest European *E. coli* outbreak in 2011. The O104:H4 outbreak strain has shown a very high level of toxin production upon induction (Laing et al., 2012). As the level of toxin production appears to be linked with the genetic polymorphism of the *stx*-phage (Lejeune et al., 2004; Eppinger et al., 2011; Smith et al., 2012), this might explain the very high HUS rate of the “new European clone.”

It should be noted that *Stx*-phage duplication was not detected with the Illumina assemblies. In all cases a single *stx*-containing contig was obtained. Only coverage analysis could detect gene duplication but insertion site(s) information was lost, along with any evolutionary context.

### Other prophages

*E. coli* strains usually contain a large number of different prophages (Hayashi et al., 2001; Ohnishi et al., 2001; Bobay et al., 2013). The O26:H11 reference strain 11368 contains 21 prophages or prophage-like elements, representing 810 kbp, in addition to nine integrative elements, representing 292 kbp. Overall these mobile genetic elements represent ~20% of the genome of the O26:H11 reference strain 11368. Although several prophages present in strain 11368 appear to be missing from the various isolates, the high non-specific mapping density on the phage structural protein genes indicates that a large number of related phages are nonetheless present in the genome of the French isolates (Supplementary Figure 2). The presence of so many phages, most of them related to the phage lambda, poses a technical challenge. Indeed the repeated presence of related structural components of the phage (head, tail, and capsid proteins coding sequences) prevents assembly with short read sequencing technology. As a result the “phageome” is dispersed on multiple short contigs that cannot be located in their chromosomal environment. In order to circumvent this problem and investigate the “phageome” of these strains we combined the Illumina short read and PacBio long read technologies. These assemblies generated fewer contigs and allowed a better resolution of the “phageome” in its chromosomal environment.

**Figure 6:**
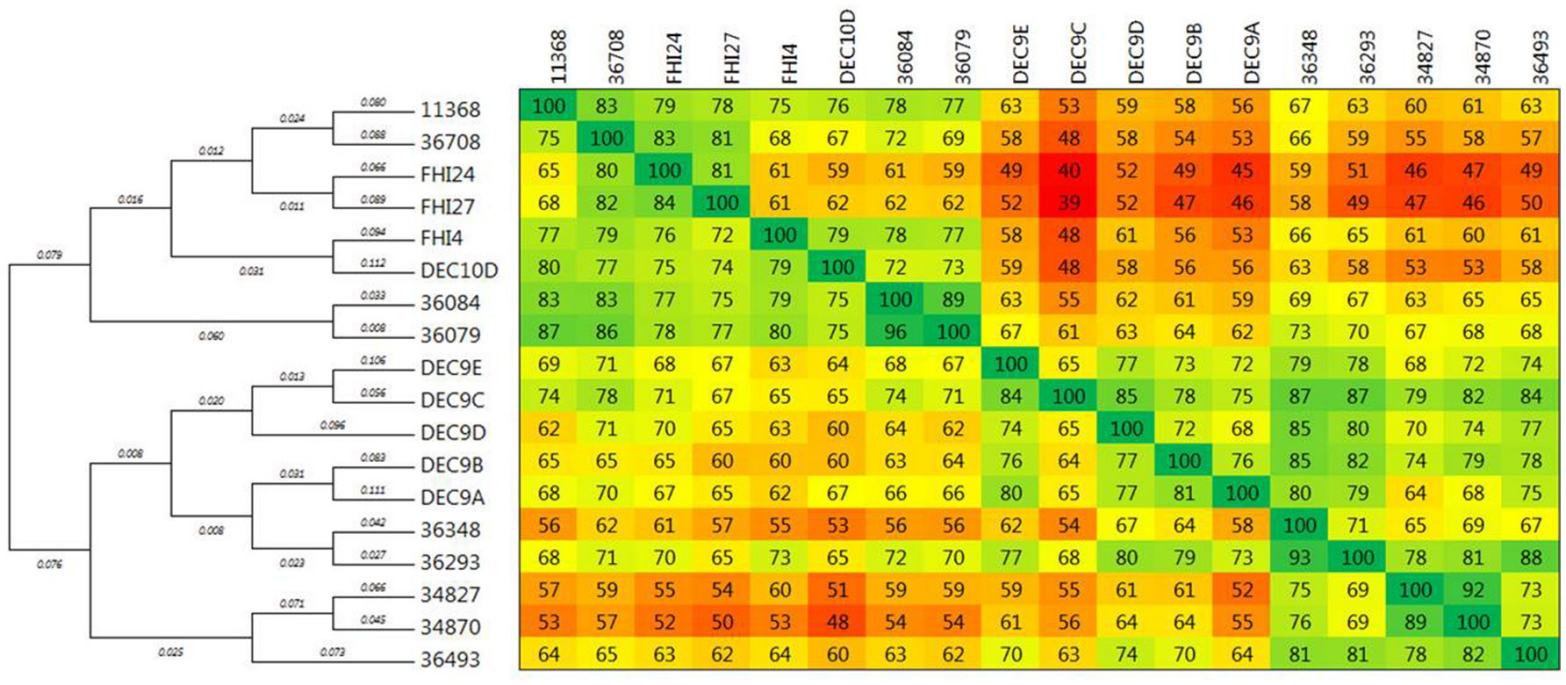
Phylogenetic relationships of the phageome of O26:H11/H- strains. The phage regions of the isolates as determined with PHASTER were compared using Gegenees 2.1 and Mega 6.06. The heat-plot comparison matrix is based on a fragmented alignment using BLASTN (200/100). The average scores of all fragment comparisons are indicated in the comparison matrix. The distance matrix was exported and used to produce a dendrogram in Mega using the Neighbor-joining method. The optimal tree with the sum of branch length = 1.78224617 is shown. The topology only of the tree is displayed with the branch length indicated next to the branches.

Using the PHASTER server, we found a large number of putative prophages and prophage-like elements in all isolates, representing between 8.68 and 16.74% of the total genome size (Table 3). Even though some of the prophages appeared incomplete, inter-prophages interactions in the prophage pool, even between different phage types, could complement defective prophages' activity (Asadulghani et al., 2009) and all prophage regions were thus included in the subsequent analysis. Annotation of the prophage regions gave a large majority of hypothetical and uncharacterized genes. This genetic “dark matter” plays a pivotal role in virulence, as the Shiga toxin gene or type III effectors genes, for example, are carried by prophages, but it can also be responsible for important phenotypic changes such as biofilm formation or adhesion and might contribute directly to host–pathogen interaction (Tobe et al., 2006; Wang et al., 2010; Mai-Prochnow et al., 2015). Comparison of the phage pool indicates that there is surprisingly little conservation between even the closely related isolates (Figure 6). The number and nature of the predicted prophages varies across the isolates (Supplementary Figure 15). While some prophages appear to be common to most isolates, no single phage is fully conserved in all isolates. On the contrary, some prophages are unique to some of the isolates (Supplementary Figure 15). For example, isolates 34827 and 34870 (*stx2a*, SNP-CC1) both harbor a prophage with high sequence similarity (Supplementary Figure 15; prophage 14 and 17, respectively) that is absent from the other isolates. Blastp analysis of this prophage indicates that it most resembles phage TL-2011b (NC_019445) isolated from a O103:H25 outbreak strain (Supplementary Data sheet 1; L'Abée-Lund et al., 2012). Similarly, a prophage most resembling *Salmonella* phage SEN34 (NC_028699) was found only in isolates 36348, 36708, DEC9D, FHI24, and FHI27. This considerable diversity in the predicted prophages illustrates the very dynamic nature of the phage genomes and how they can shape the bacterial chromosome. Surprisingly, trees generated from the comparison of the “phageome” alone or from the core genome analysis by SNP genotyping and wgSNPs all clustered the strains or their respective phage regions in similar groups (Figure 6). Interestingly, the prophages appear comparably distributed along the chromosome in all isolates compared to the O26:H11 reference strain 11368 (Supplementary Figure 16). This suggests that although the phage flow is important, phages probably always use a limited number of integration sites or hot spots.

**Table 3:** Size of the mobilome (plasmids and phages) of the O26:H11/H- isolates.

**Isolate**	**Total Genome size (bp)**	**Chromosome size (bp)**	**Number of large plasmids**	**Total plamid size (bp)**	**Number of phages**	**Size of “phageome” (bp)**
36,084	5,849,490	5,757,624	1	91,866	22	904,600
36,079	5,735,083	5,658,663	1	76,420	21	859,100
36,708	5,894,762	5,770,096	1	124,666	24	987,000
34,870	5,798,318	5,598,857	2	199,461	23	897,200
34,827	5,933,672	5,639,586	4	294,086	18	840,500
36,293	5,790,328	5,544,723	2	245,605	20	757,200
36,493	5,758,521	5,537,274	2	221,247	20	720,700
36,348	5,978,287	5,732,373	2	245,914	22	939,100
FHI4	5,486,605	5,293,109	2	193,496	18	638,800
FHI24	5,612,104	5,504,369	1	107,735	23	831,300
FHI27	5,588,374	5,460,140	1	128,234	23	802,700
DEC9A	5,408,446	5,408,446	–	–	17	564,900
DEC9B	5,361,604	5,361,604	–	–	16	630,100
DEC9C	5,194,722	5,194,722	–	–	13	450,700
DEC9D	5,485,621	5,422,169	1	63,452	19	648,400
DEC9E	5,430,771	5,430,771	–	–	18	647,700
DEC10D	5,404,073	5,295,503	2	108,570	17	660,200

### Plasmids

Different putative, large plasmids carrying various sets of adhesion and accessory virulence-associated genes were found in the strains (Table 4). Some plasmids also carry antibiotic resistance genes in addition to virulence genes (*IncQ1, IncI1* plasmid in strain FHI4 and *IncFII* plasmid in strain 36493). Most plasmids appear to contain numerous IS elements as well as putative phage-related sequences. *ColE1-like* replicons were found using the Illumina reads indicating the presence of small plasmids, but these were absent from the PacBio assembly due to size selection of fragments at 10 kb during library construction. Because of the fragmentation of the Illumina data the small plasmids were not investigated here.

**Table 4:** Distribution of large plasmids in the O26:H11/H- isolates.

**SNP-CC**	**Strain**	Plasmid size (bp)^*^	**Plasmid replication types**	**Plasmid-encoded traits**	**Similar known sequenced plasmid (Accession number)—Size (bp)**
SNP-CC3	36084	91,866^*^^#^	*IncB/O/K/Z, IncFIB*	*ehxA, toxB, katP, espP, colM*	pO26-1 (NC_013369)—85,167
	36079	76,420^*^^#^	*IncB/O/K/Z, IncFIB*	*ehxA, toxB, katP, espP, colM*	pO26-1 (NC_013369)—85,167
	FHI4	80,100^#^	*IncB/O/K/Z, IncFIB*	*ehxA, toxB, espP, colM*	pO26-1 (NC_013369)—85,167
		113,396^#^	*IncQ1, IncI1*	Large complex resistance locus (*strA, strB, aadA1, blaTEM1B, sul1, sul2, tetA, dfrA1, qacE*), type II secretion system, colicin	pE17-16 (NC_024975)—101,321
	DEC10D	85,715^#^	*IncB/O/K/Z, IncFIB*	*ehxA, toxB, espP*	pO26-1 (NC_013369)—85,167
		22,855^*^	*IncFII*	Type IV conjugative transfer system (incomplete)	pO26-2 (NC_013362)—63,365
SNP-CC2	36708	124,666^*^^#^	*IncFIB, IncFII*	*ehxA, etpD, colB, colM, stcE*, type IV conjugative transfer system	pO145-12761 (NZ_CP007135)—98,067
	FHI24	107,735^#^	*IncFIB, IncFII*	*ehxA, etpD, colB, colM, stcE*	pO145-12761 (NZ_CP007135)—98,067
	FHI27	128,234^#^	*IncFIB, IncFII*	*ehxA, etpD, colB, colM*	pO145-12761 (NZ_CP007135)—98,067
	34870	161,837^*^^#^	*IncI1*	*hlyA, AidAI, lda*	pHUSEC2011-1 (HE610900)—88,546
SNP-CC1	34827	37,624^*^	*IncX1*	Type IV secretion system conjugation apparatus	pOLA52 (EU370913)—51,602
		161,706^#^	*IncI1*	*hlyA, AidAI, lda*	pHUSEC2011-1 (HE610900)—88,546
		35,343^*^	*IncX1*	Type IV secretion system conjugation apparatus	pOLA52 (EU370913)—51,602
		31,084^*^	*IncX4*		pCROD2 (NC_013718)—39,265
		65,953^*^	*IncI2*		pRM12761 (NZ_CP007134)—58,666
	36293	162,963^#^	*IncI1*	*hlyA, AidAI, lda*	pHUSEC2011-1 (HE610900)—88,546
		82,642	*IncB/O/K/Z*	Type IV transfer system	pO113 (NC_007365)—165,548
	36348	153,502^#^	*IncI1*	*hlyA, AidAI, lda*	pHUSEC2011-1 (HE610900)—88,546
		92,412^*^^#^	*IncB/O/K/Z*	*epeA*, type IV transfer system	pO113 (NC_007365)—165,548
	36493	132,780^#^	*IncI1*		pHUSEC2011-1 (HE610900)—88,546
		88,467	*IncFII*	*colB, colM, aadA1, strA, strB, blaOXA-1, mph(A), sul1*	pEC-B24 (GU371926)—73,801
	DEC9D	63,452	*IncFII*	Type IV conjugative transfer system	pEC-B24 (GU371926)—73,801

* *Indicates plasmids present on a single contig*.

# *Indicates plasmids where putative phage-related sequences were identified by the PHASTER server*.

While large plasmids were found in most strains, no large plasmid could be found in the WGS data of strains DEC9A, DEC9B, DEC9C, and DEC9E. It is impossible for us to determine if these strains are actually devoid of large plasmids or if this is due to plasmid loss during DNA extraction, library preparation, sequencing artifacts, or assembly artifacts.

All strains exhibited variable plasmid contents and can be classified in three groups according to their plasmid content corresponding to the clonal complexes. SNP-CC3 strains (36084, 36079, FHI4, and DEC10D) contain an *IncB/O/K/Z, IncFIB* pO26-1-like plasmid with or without other plasmids. SNP-CC2 strains (36708, FHI24, FHI27) contain an *IncFIB, IncFII* plasmid; the closest known previously sequenced plasmid was pO145-12761 from the O145:H28 strain RM12761 (isolated from ice cream during a 2007 ice cream-associated outbreak in Belgium; Cooper et al., 2014a). SNP-CC1 (36293, 36493, 34870, and 34827) strains carry an *IncI1* pHUSEC2011-1-like plasmid (from the O104:H4 strain that caused the 2011 epidemic) with or without additional plasmids. This pHUSEC2011-1-like plasmid notably contains the alpha-hemolysin operon, the *AidAI* autotransporter gene (an adhesin involved in diffuse adherence), and the locus for diffuse adherence (*lda*) originally described as a chromosomal locus in EPEC O26 strains in Brazil (Scaletsky et al., 2005). A previous study involving strain 36493 has demonstrated that this plasmid was transferable (Jost et al., 2016). Additionally, when related plasmids were present in more than one isolate, they showed evidence of gene variation. The observed variability in plasmid profiles once again highlights the genomic plasticity that exists even among closely related isolates and these clearly contribute to the intra-serotype diversity.

## Conclusion

This study aimed at determining the phylogenetic relationships and comparing the genetic structure of *stx2*-positive *E. coli* O26:H11 circulating in France. The STEC O26:H11 isolates compared in this study were obtained from patients with HUS in France and in Norway. *Stx*-negative and *stx1*-positive O26:H11 strains isolated from patients with diarrhea were also included (Table 1).

Although the French strains had been previously sequenced (Delannoy et al., 2015b), we greatly improved the quality of the sequences by using PacBio sequencing (Supplementary Table 1). The alignment of high quality Illumina short reads with a reduced number of informative PacBio long reads generated high quality, low contig number Illumina/PacBio hybrid sequences. This allowed us to perform a detailed analysis of the genomic heterogeneity of the strains. We have explored the relationships between the strains by investigating both the core genome using whole genome comparison, defined sets of SNPs and wgSNPs, and the “mobilome” by looking at phages and plasmids.

Our data indicated that several clones of EHEC O26:H11 are co-circulating in France, as strains from distinct clonal complexes (SNP-CC1, -CC2, and -CC3, Figure 2) were found in this set of strains isolated within a one-year span (Delannoy et al., 2015a). A phylogenic analysis distributed the strains in two lineages (Figure 3). Both the ST21 strains (SNP-CC3 and SNP-CC4) and the ST29 “new European clone” (SNP-CC2) were found to belong to the same lineage. The “new French clone” (SNP-CC1) was found to belong to a distinct lineage that appears genetically more closely related to AEEC strains. American bovine AEEC strains can also be found in this clonal lineage (Gonzalez-Escalona et al., 2016). Interestingly, the wgSNP phylogeny is summarized in the *cas* gene phylogeny (Figure 4), and a simple qPCR assay targeting the CRISPR array specific to SNP-CC1 (SP_O26-E) can distinguish between the two main lineages. The simultaneous presence of strains with various pathogenic potential in the same cluster suggests that the clonal lineage of the strains is a poor predictor of pathogenicity. As previously demonstrated for O157 and various non-O157 strains (Ogura et al., 2009; Cooper et al., 2014b; Rusconi et al., 2016), the genetic repertoire of the mobilome will most likely affect the potential virulence and host specificity of the strains.

Despite the remarkable similarity of their chromosomal backbone (Figure 1 and Supplementary Figure 1), the isolates display, on the contrary, a surprisingly diverse mobilome with a large number of prophages and plasmids (Table 3). The genomic heterogeneity of the mobilome makes it a major contributor to O26:H11 intra-serotype diversity. Specifically, the complex *stx*-phage pattern suggests the parallel emergence of several clones with acquisition of distinct *stx*-phages alongside other phages. The presence of the same *stx*-prophage in strains belonging to the different lineages (SNP-CC1 and SNP-CC3) suggests that its insertion in the genome predates the divergence of the “French clone” as a separate lineage from the “progenitor” (Bletz et al., 2013). The presence of a different *stx*-converting prophage at a different location in the “European clone” (SNP-CC2) suggests a posterior divergence with separate loss and acquisition events. Interestingly, all ST29 strains as well as the Norwegian ST21 strain have a *stx2a* gene identical to that of the O104:H4 strain that caused the 2011 epidemic. It is noteworthy that the same *stx2a* allele is present in prophages with divergent genetic architecture and chromosomal insertion sites, thus suggests different origins (Ogura et al., 2009). As a major contributor of EHEC pathogenesis, the Shiga toxin is of special interest. The *stx* subtypes and gene dosage, as well as the *stx*-converting phage environment and insertion sites, are all elements that may play a direct role in toxin production level and disease severity (Friedrich et al., 2002; Lejeune et al., 2004; Eppinger et al., 2011; Laing et al., 2012; Smith et al., 2012; Rusconi et al., 2016; Ishijima et al., 2017); however, each of their exact roles remain to be fully elucidated.

The clustering of the strains using the core genome, phage genome, or plasmid complement all divide the strains in similar groups (Figures 3, 6 and Table 4). Plasmids and prophages are a cornucopia of new genes that will modify the behavior of the bacteria. Antimicrobial resistance genes, genes modifying the colonization and adhesion properties, toxins, and other virulence genes, for example, were all found in the various isolates on self-transmissible elements. It is thus possible that the mobilome drives the evolution of the core genome by subtle—or not so subtle—changes in host-pathogen interactions, adaptation to new ecological niches, increased virulence, etc. As apparition of a new clonal lineage can be linked to selective pressure during transfer of the population in a new ecological niche, it is possible that the various clonal lineages have different reservoirs. Indeed, while strains from the lineage encompassing SNP-CC2, SNP-CC3 and SNP-CC4 can be found in humans and cattle, *stx*-positive strains from the SNP-CC1 lineage have only been isolated from human, rarely from other sources (Zhang et al., 2000; Bielaszewska et al., 2013; Zweifel et al., 2013; Douëllou et al., 2016, 2017; Germinario et al., 2016; Gonzalez-Escalona et al., 2016; Ishijima et al., 2017). Only *stx*-negative strains (AEEC) from the SNP-CC1 lineage have been isolated from cattle and dairy products until now (Zweifel et al., 2013; Douëllou et al., 2016, 2017; Gonzalez-Escalona et al., 2016), although it is not clear if this is due to a sampling or isolation bias or a different reservoir.

Overall, the MGEs appear to play a major role in O26:H11 intra-serotype clonal diversification.

## Author contributions

Conceived and designed the experiments: SD, PF. Performed the experiments: SD, PM, SB. Analyzed the data: SD, PM, SB, PF. Contributed reagents/materials/analysis tools: SD, PM, SB, PF. Wrote the paper: SD, PF. Critical revision of the paper for important intellectual content: SD, PM, HW, SB, PF.

### Conflict of interest statement

The authors declare that the research was conducted in the absence of any commercial or financial relationships that could be construed as a potential conflict of interest.
