# Supplementary figures and images for: The Mobilome; A Major Contributor to Escherichia coli stx2-Positive O26:H11 Strains Intra-Serotype Diversity
Source: Front Microbiol. 2017 Sep 6;8:1625. doi: 10.3389/fmicb.2017.01625 (PMC5592225; doi:10.3389/fmicb.2017.01625)

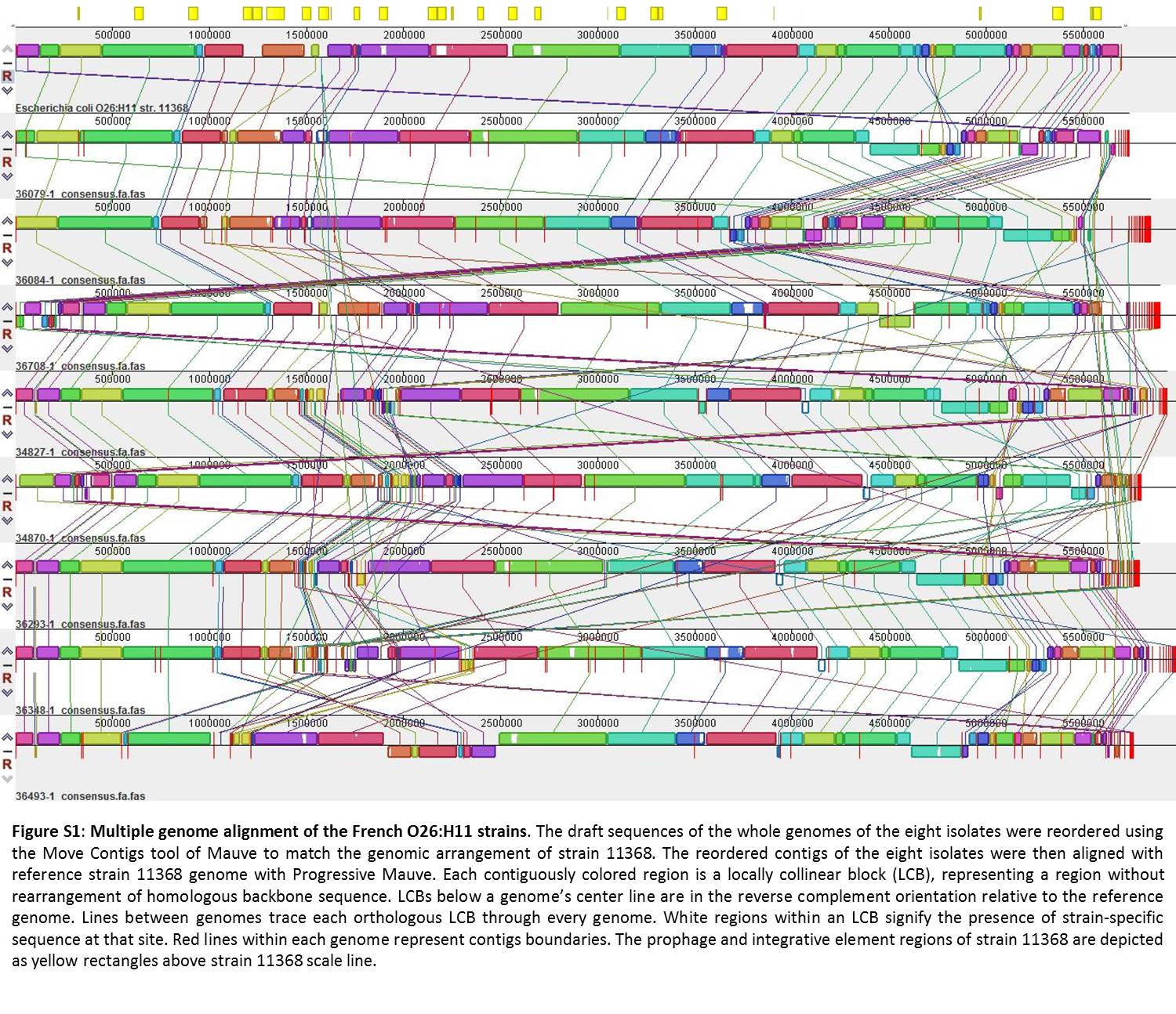

Supplement: Supplementary file 10 [file Image1.JPEG]

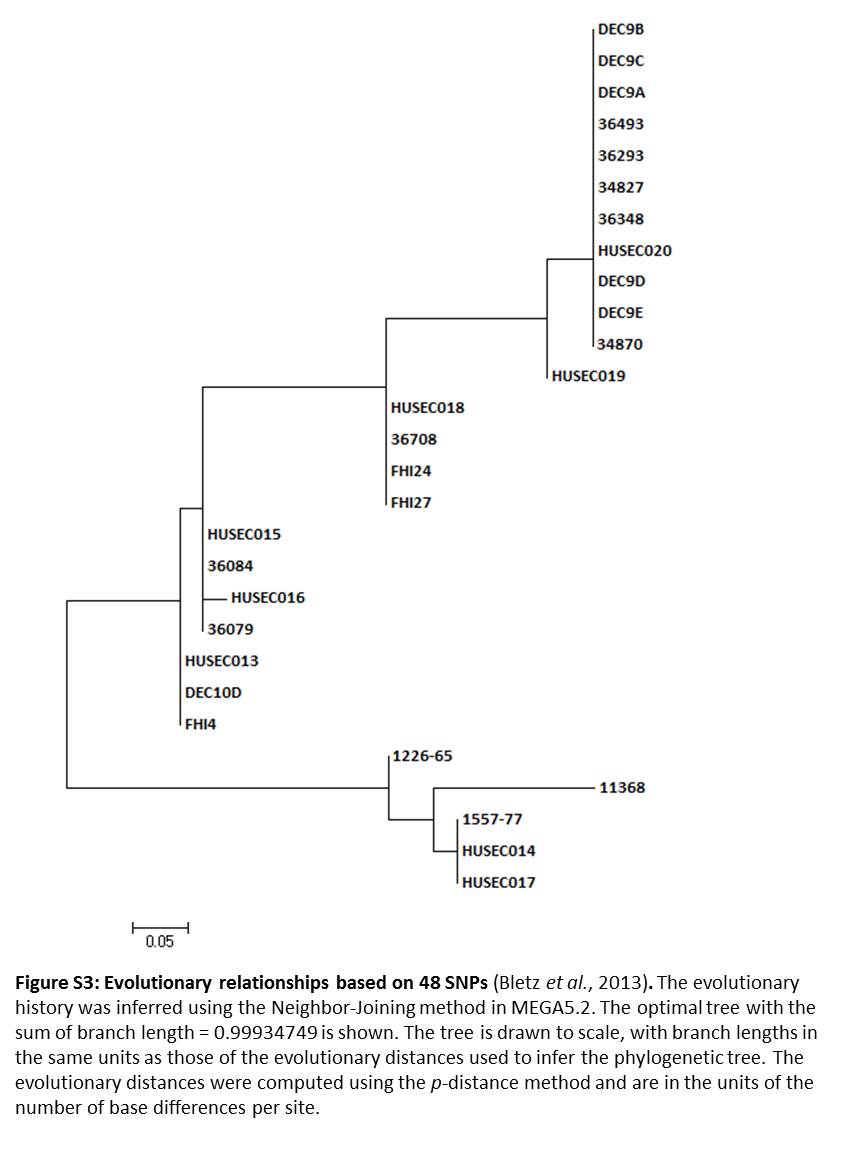

Supplement: Supplementary file 12 [file Image3.JPEG]

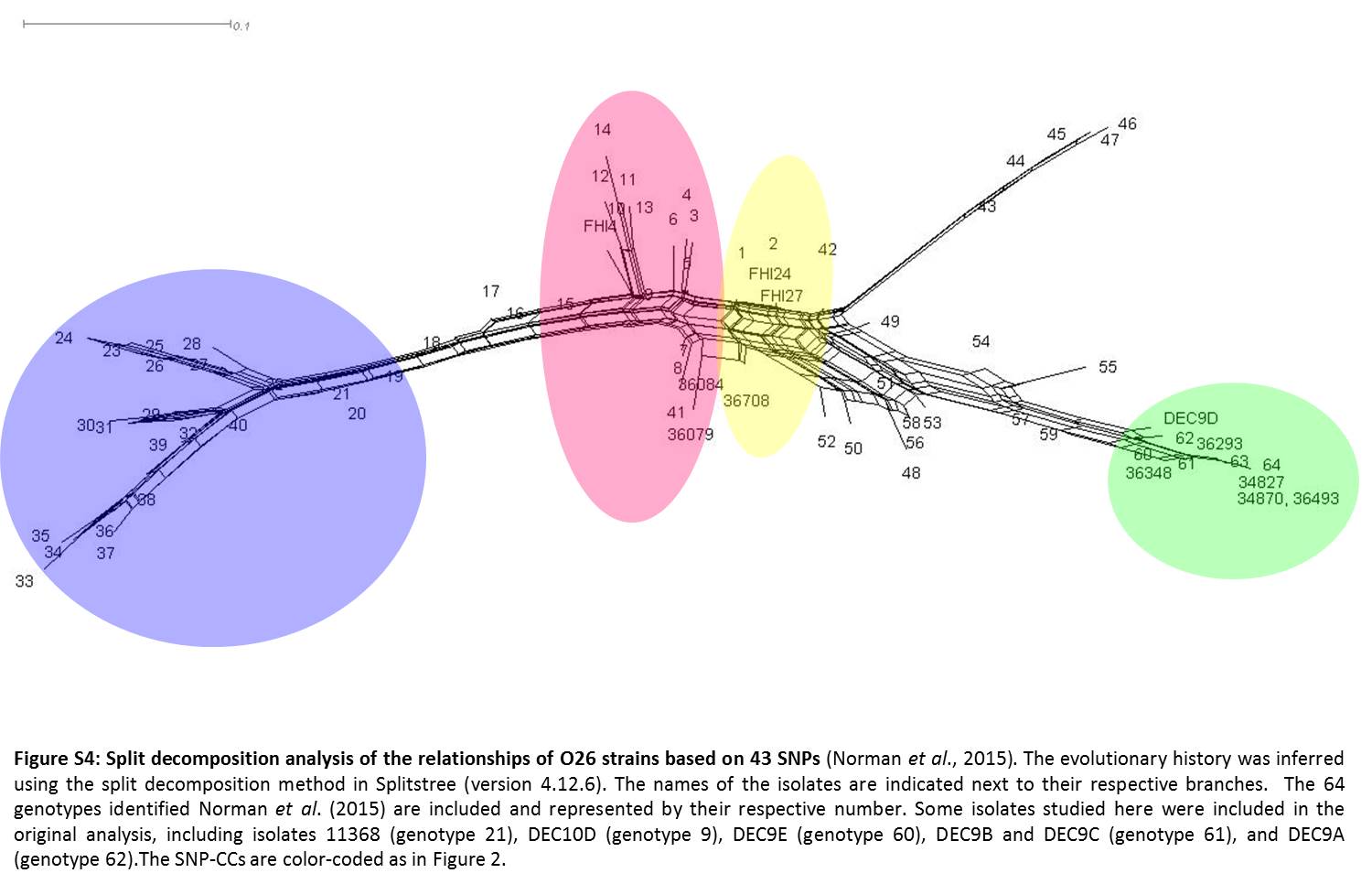

Supplement: Supplementary file 13 [file Image4.JPEG]

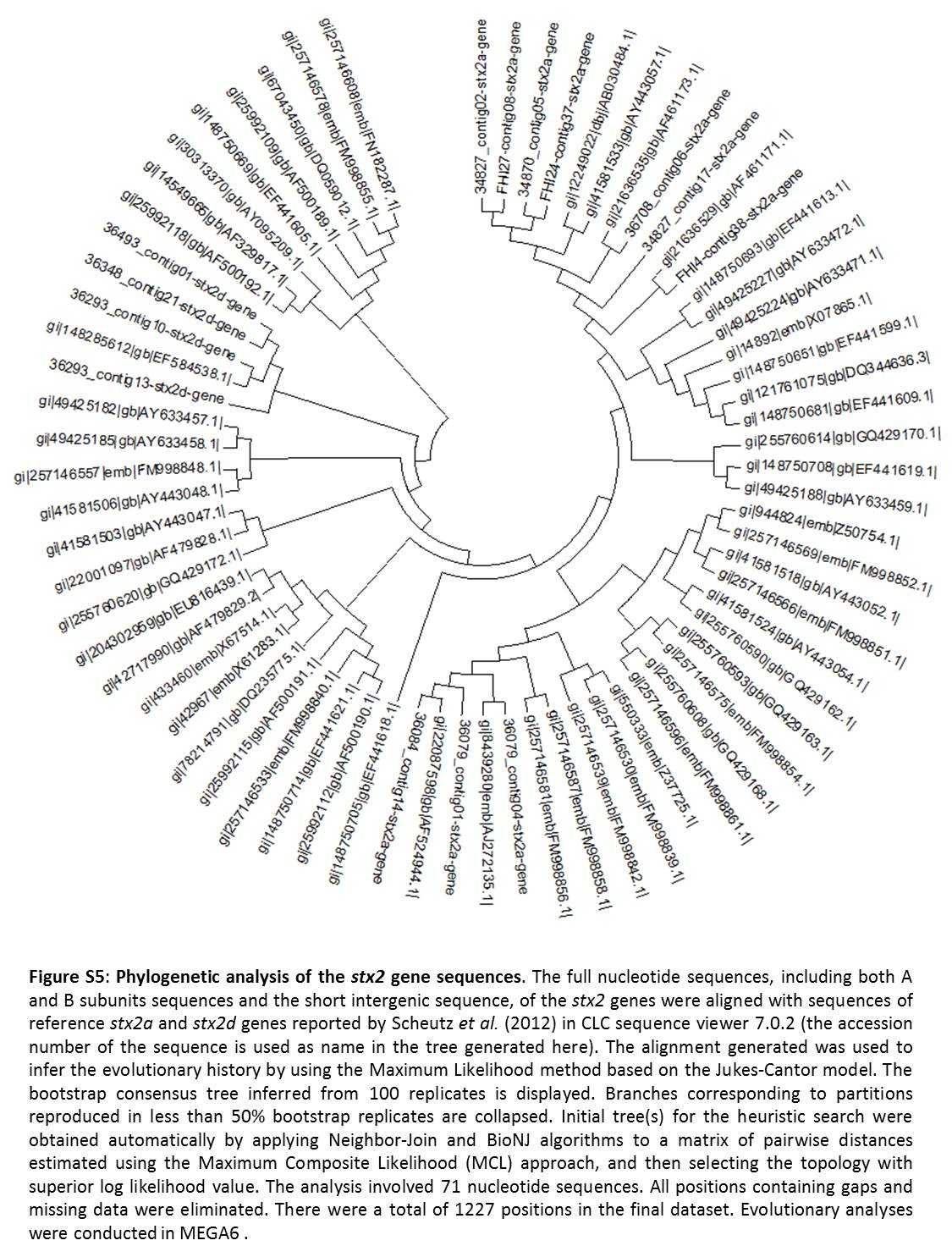

Supplement: Supplementary file 14 [file Image5.JPEG]

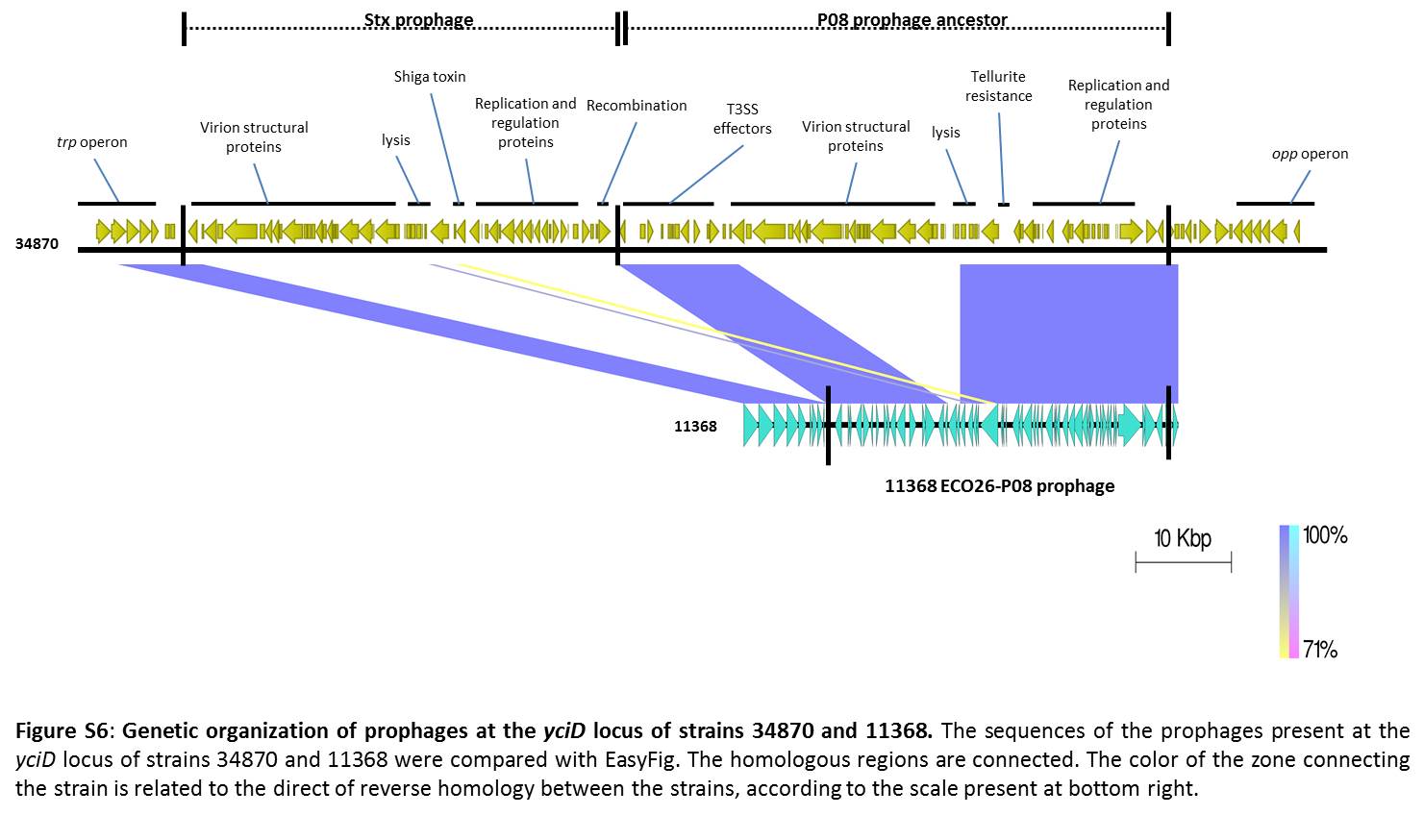

Supplement: Supplementary file 15 [file Image6.JPEG]

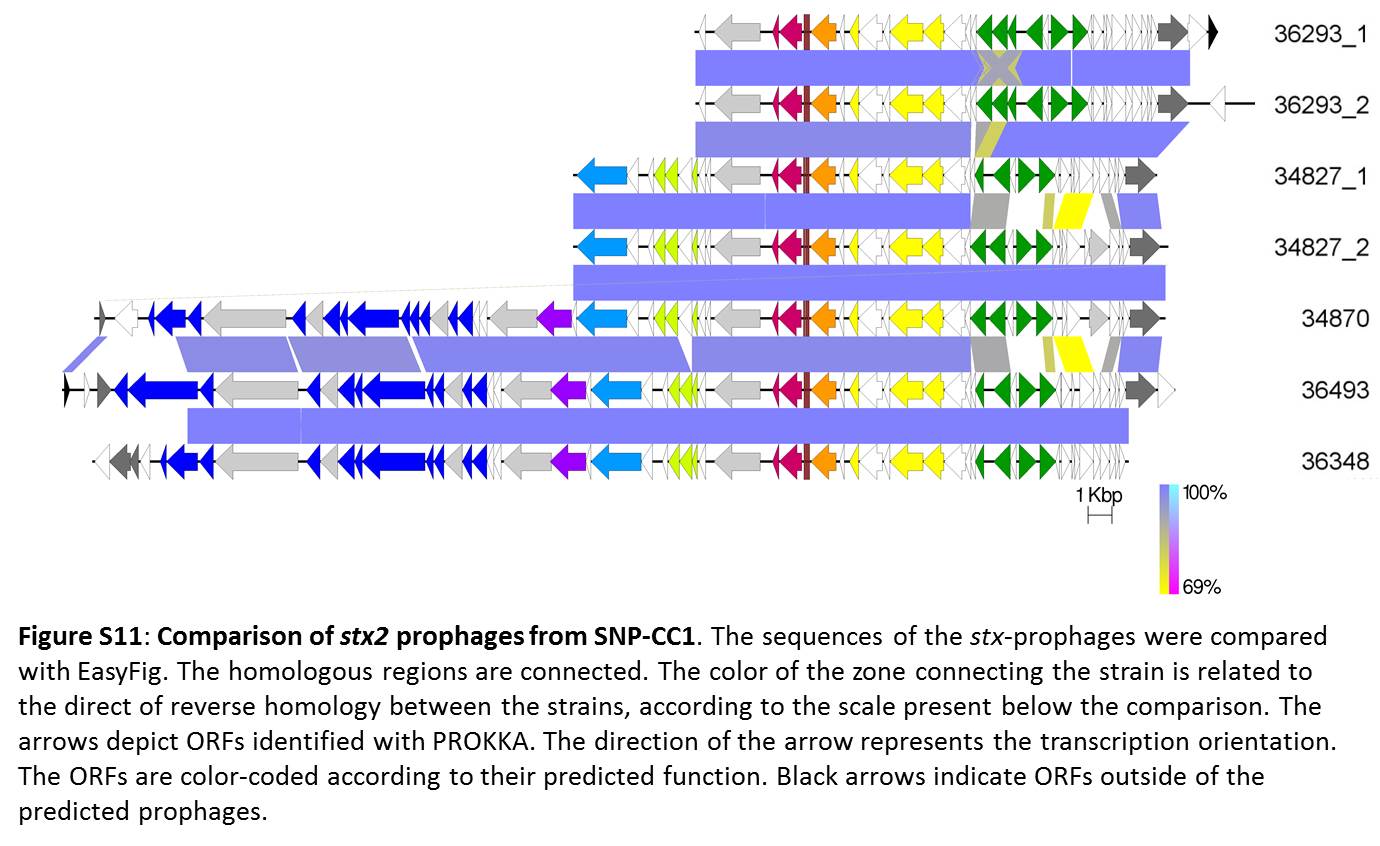

Supplement: Supplementary file 20 [file Image11.JPEG]

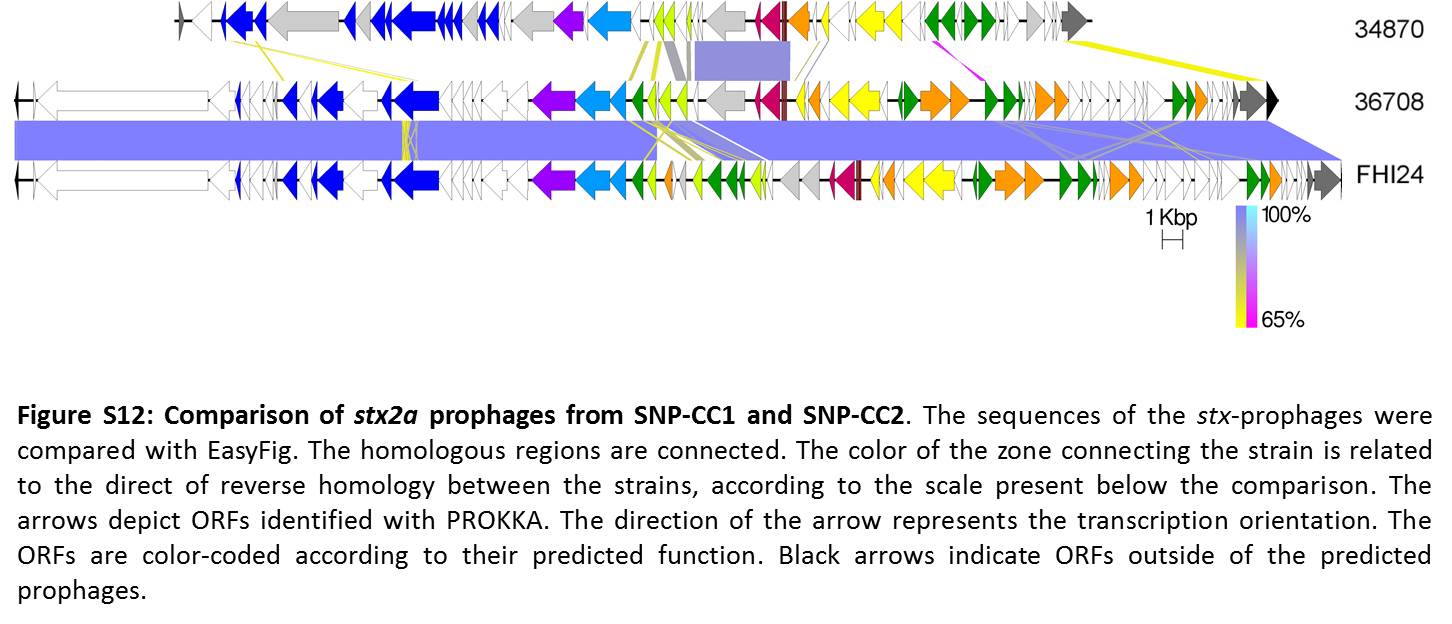

Supplement: Supplementary file 21 [file Image12.JPEG]

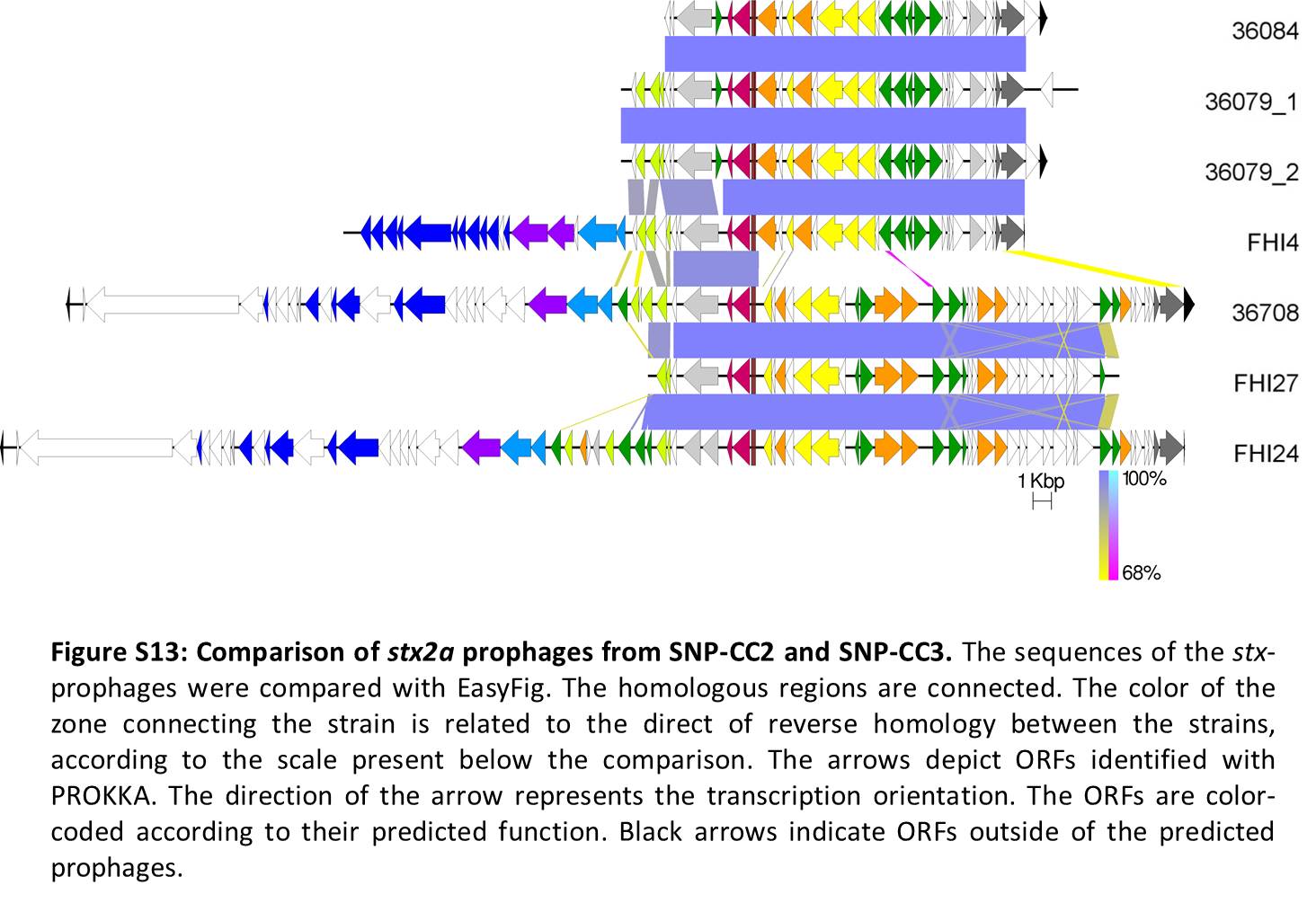

Supplement: Supplementary file 22 [file Image13.JPEG]

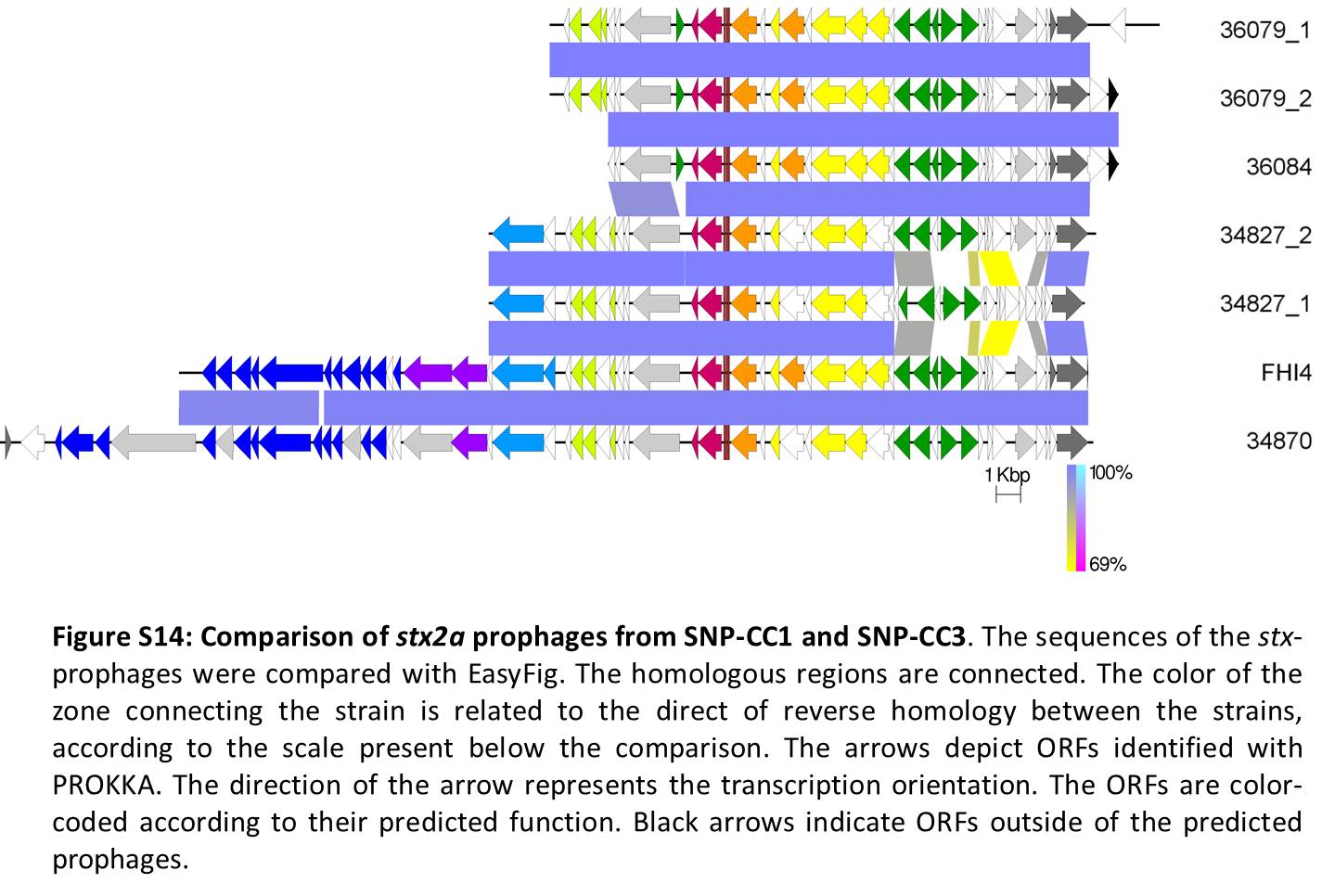

Supplement: Supplementary file 23 [file Image14.JPEG]

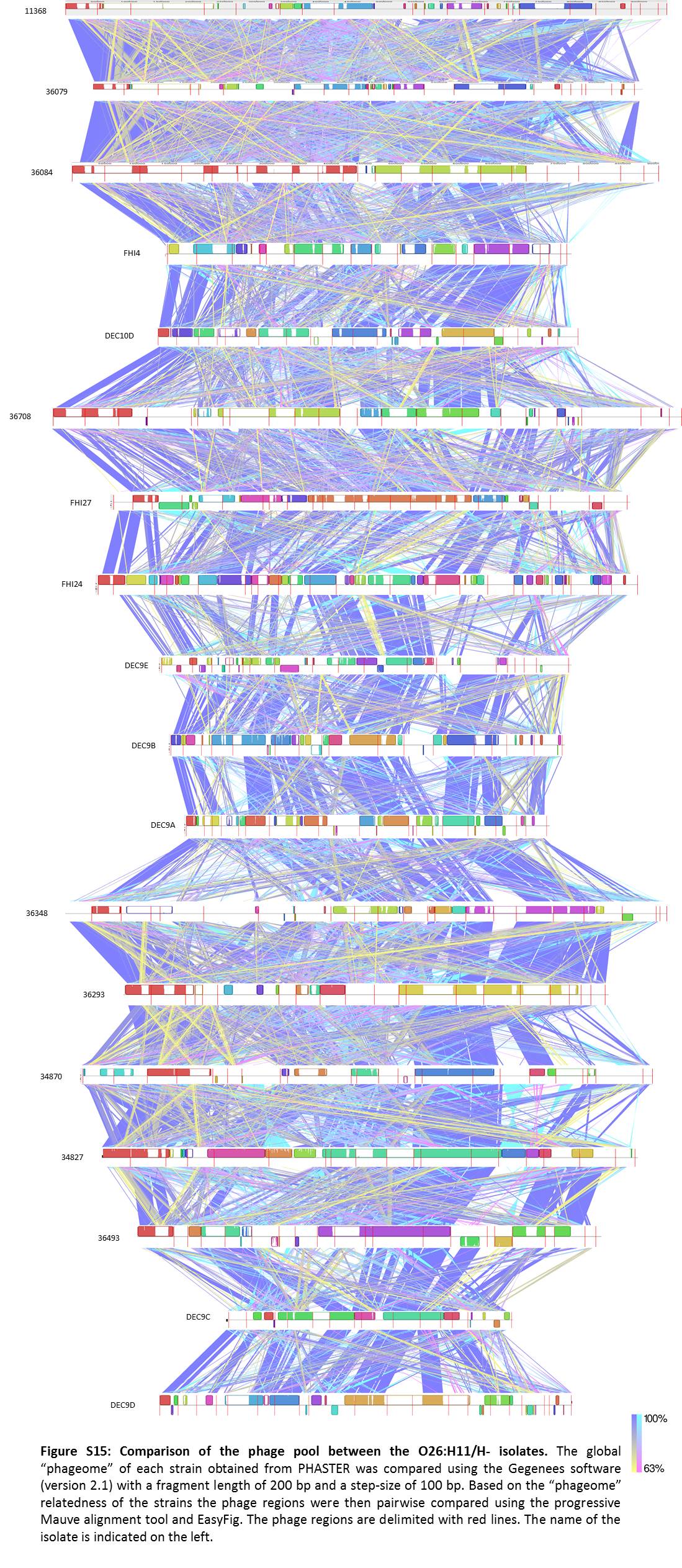

Supplement: Supplementary file 24 [file Image15.JPEG]
