# Supplementary material for: The Mobilome; A Major Contributor to Escherichia coli stx2-Positive O26:H11 Strains Intra-Serotype Diversity
Source: Front Microbiol. 2017 Sep 6;8:1625. doi: 10.3389/fmicb.2017.01625 (PMC5592225; doi:10.3389/fmicb.2017.01625)
Supplement: Supplementary file 11 [file Image2.PDF]

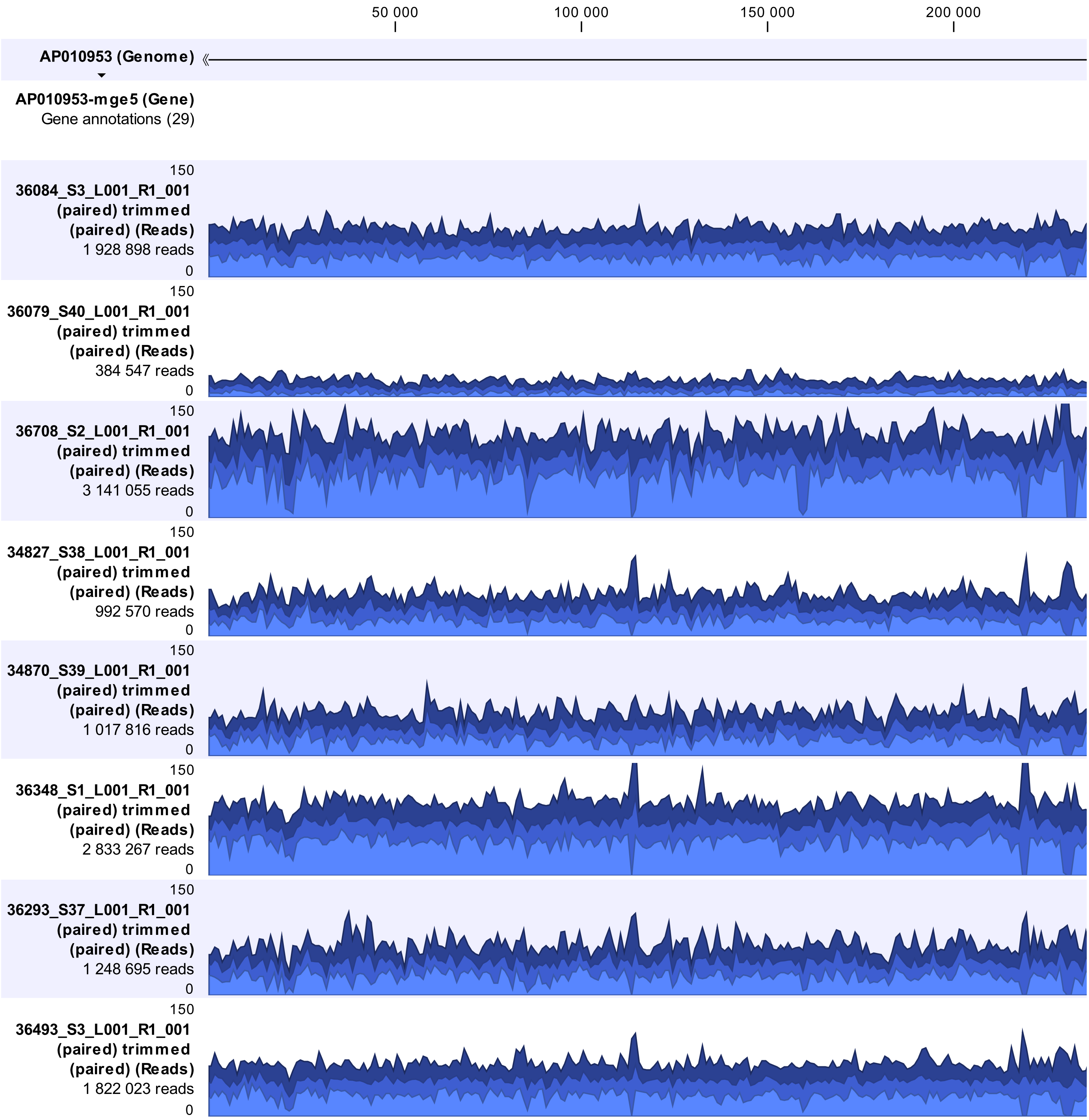
50 000
100 000
150 000
200 000
AP010953 (Genome)
AP010953-mge5 (Gene)
Gene annotations (29)
150
36084_S3_L001_R1_001 (paired) trimmed (paired) (Reads)
1 928 898 reads
0
150
36079_S40_L001_R1_001 (paired) trimmed (paired) (Reads)
384 547 reads
0
150
36708_S2_L001_R1_001 (paired) trimmed (paired) (Reads)
3 141 055 reads
0
150
34827_S38_L001_R1_001 (paired) trimmed (paired) (Reads)
992 570 reads
0
150
34870_S39_L001_R1_001 (paired) trimmed (paired) (Reads)
1 017 816 reads
0
150
36348_S1_L001_R1_001 (paired) trimmed (paired) (Reads)
2 833 267 reads
0
150
36293_S37_L001_R1_001 (paired) trimmed (paired) (Reads)
1 248 695 reads
0
150
36493_S3_L001_R1_001 (paired) trimmed (paired) (Reads)
1 822 023 reads
0

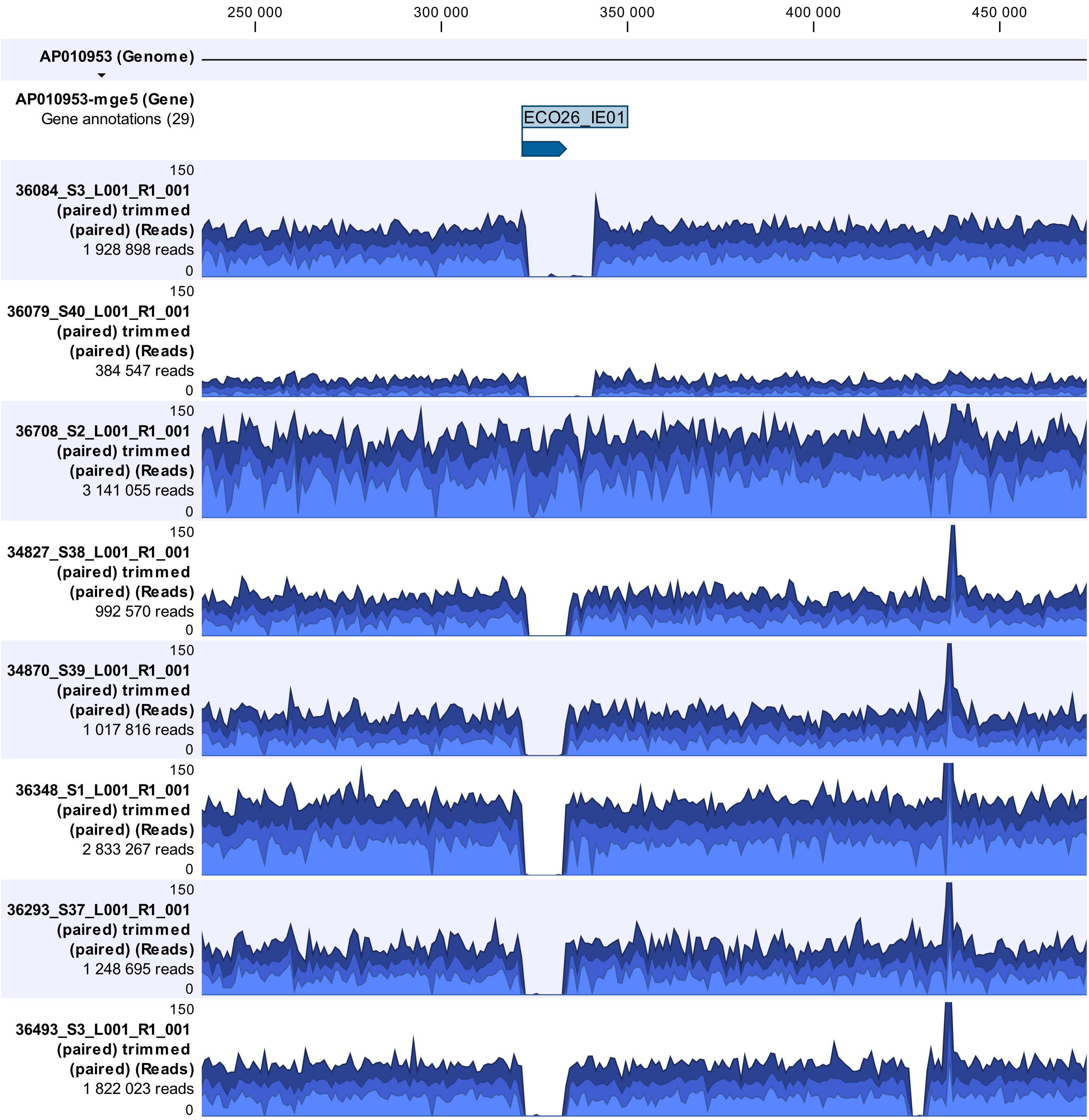
250 000
300 000
350 000
400 000
450 000
AP010953 (Genome)
AP010953-mge5 (Gene)
Gene annotations (29)
ECO26_IE01
150
36084_S3_L001_R1_001 (paired) trimmed (paired) (Reads)
1 928 898 reads
0
150
36079_S40_L001_R1_001 (paired) trimmed (paired) (Reads)
384 547 reads
0
150
36708_S2_L001_R1_001 (paired) trimmed (paired) (Reads)
3 141 055 reads
0
150
34827_S38_L001_R1_001 (paired) trimmed (paired) (Reads)
992 570 reads
0
150
34870_S39_L001_R1_001 (paired) trimmed (paired) (Reads)
1 017 816 reads
0
150
36348_S1_L001_R1_001 (paired) trimmed (paired) (Reads)
2 833 267 reads
0
150
36293_S37_L001_R1_001 (paired) trimmed (paired) (Reads)
1 248 695 reads
0
150
36493_S3_L001_R1_001 (paired) trimmed (paired) (Reads)
1 822 023 reads
0

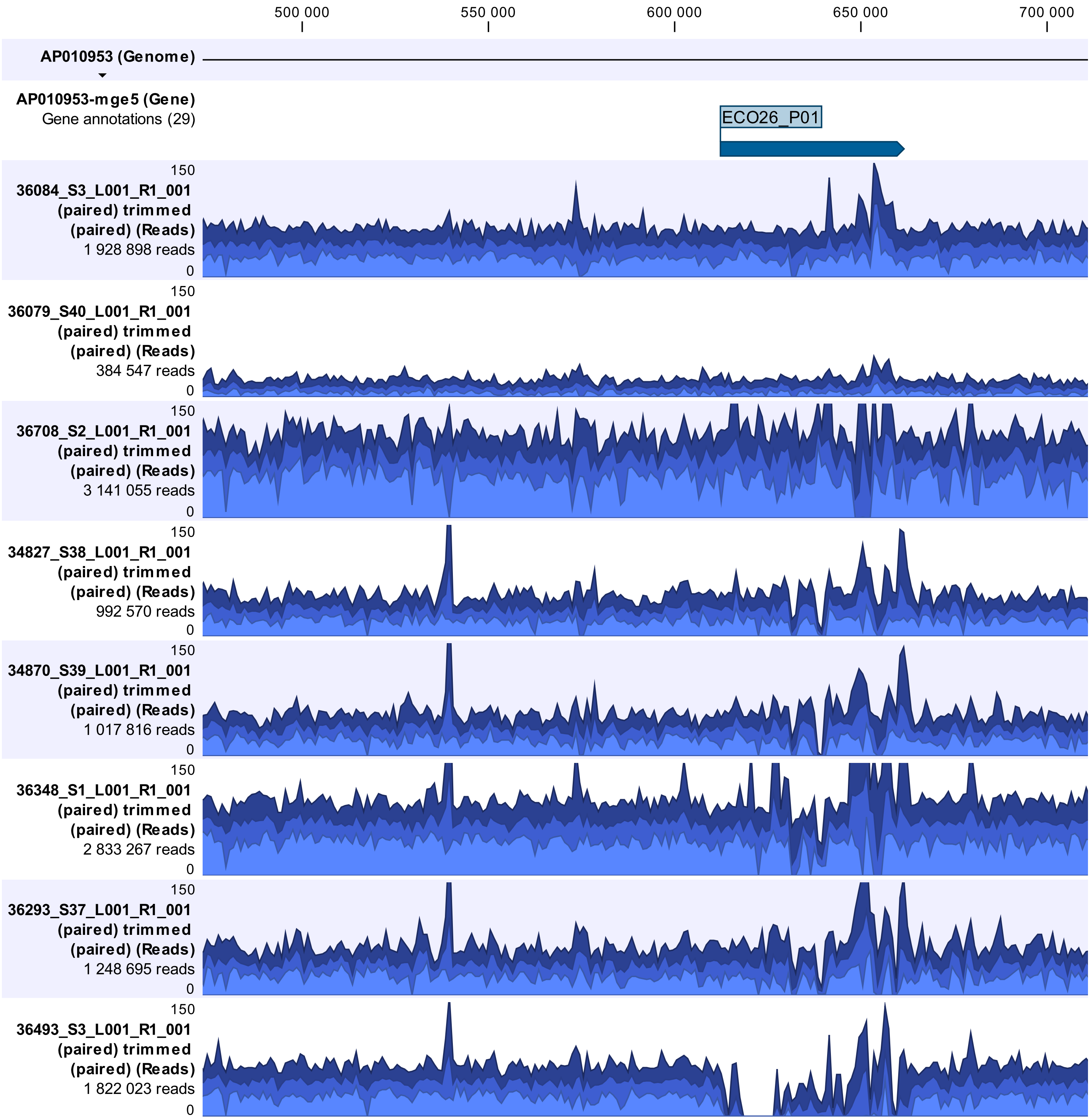
500 000
550 000
600 000
650 000
700 000
AP010953 (Genome)
AP010953-mge5 (Gene)
Gene annotations (29)
ECO26_P01
150
36084_S3_L001_R1_001 (paired) trimmed (paired) (Reads)
1 928 898 reads
0
150
36079_S40_L001_R1_001 (paired) trimmed (paired) (Reads)
384 547 reads
0
150
36708_S2_L001_R1_001 (paired) trimmed (paired) (Reads)
3 141 055 reads
0
150
34827_S38_L001_R1_001 (paired) trimmed (paired) (Reads)
992 570 reads
0
150
34870_S39_L001_R1_001 (paired) trimmed (paired) (Reads)
1 017 816 reads
0
150
36348_S1_L001_R1_001 (paired) trimmed (paired) (Reads)
2 833 267 reads
0
150
36293_S37_L001_R1_001 (paired) trimmed (paired) (Reads)
1 248 695 reads
0
150
36493_S3_L001_R1_001 (paired) trimmed (paired) (Reads)
1 822 023 reads
0

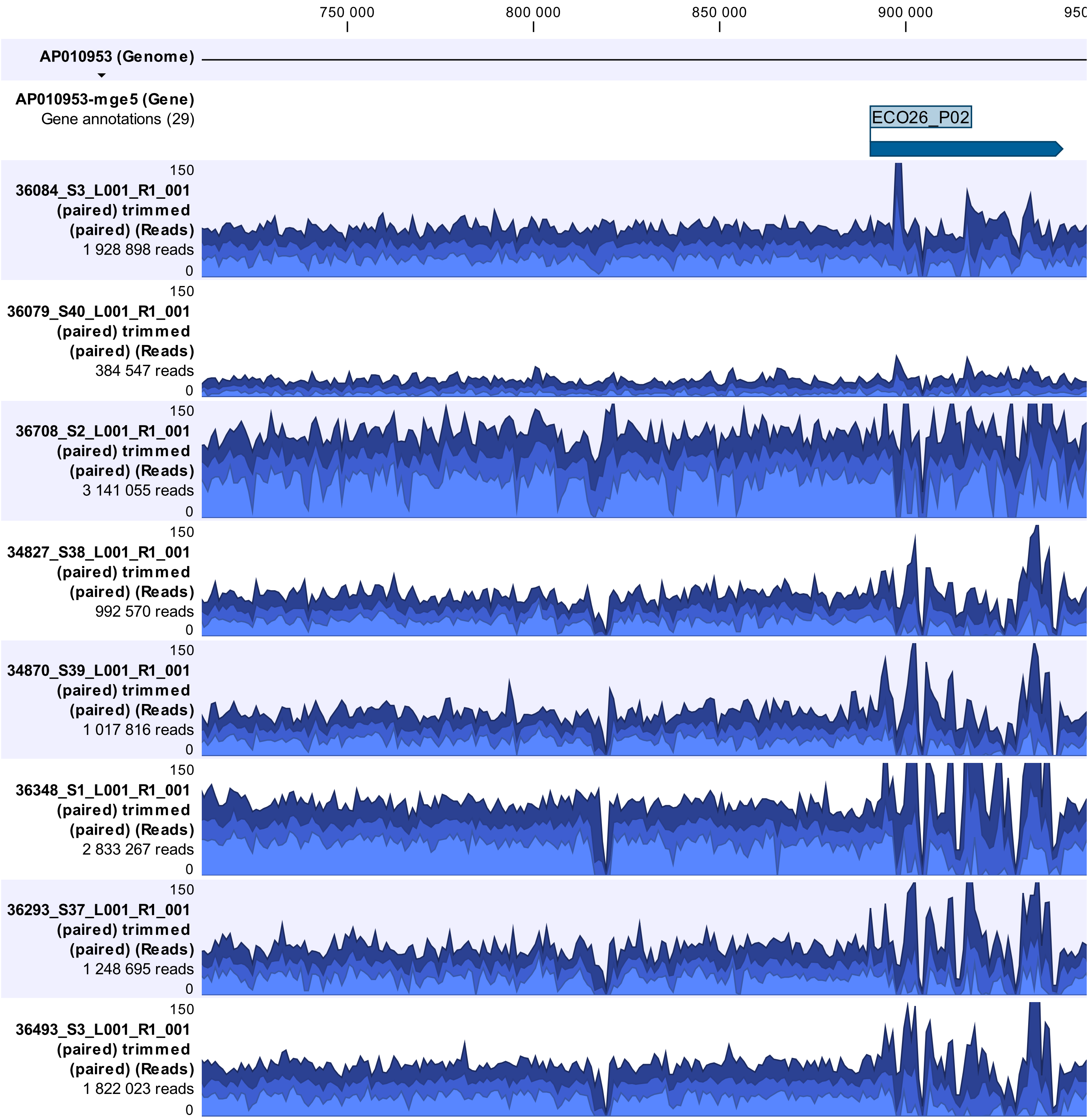
750 000
800 000
850 000
900 000
95(
AP010953 (Genome)
AP010953-mge5 (Gene)
Gene annotations (29)
ECO26_P02
150
36084_S3_L001_R1_001 (paired) trimmed (paired) (Reads)
1 928 898 reads
0
150
36079_S40_L001_R1_001 (paired) trimmed (paired) (Reads)
384 547 reads
0
150
36708_S2_L001_R1_001 (paired) trimmed (paired) (Reads)
3 141 055 reads
0
150
34827_S38_L001_R1_001 (paired) trimmed (paired) (Reads)
992 570 reads
0
150
34870_S39_L001_R1_001 (paired) trimmed (paired) (Reads)
1 017 816 reads
0
150
36348_S1_L001_R1_001 (paired) trimmed (paired) (Reads)
2 833 267 reads
0
150
36293_S37_L001_R1_001 (paired) trimmed (paired) (Reads)
1 248 695 reads
0
150
36493_S3_L001_R1_001 (paired) trimmed (paired) (Reads)
1 822 023 reads
0

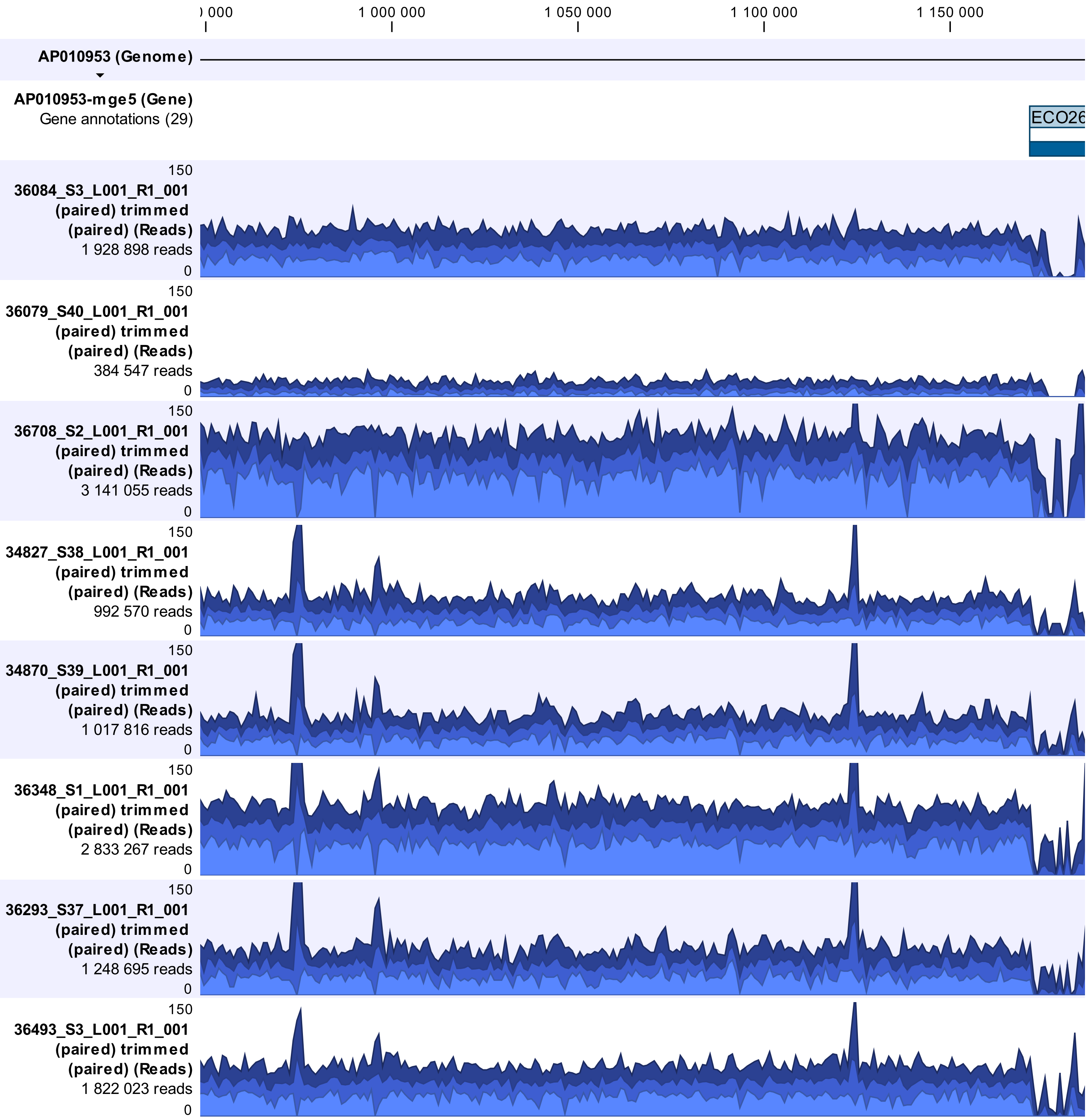

) 000
1 000 000
1 050 000
1 100 000
1 150 000
AP010953 (Genome)
AP010953-mge5 (Gene)
Gene annotations (29)
ECO26
150
36084_S3_L001_R1_001 (paired) trimmed (paired) (Reads)
1 928 898 reads
0
150
36079_S40_L001_R1_001 (paired) trimmed (paired) (Reads)
384 547 reads
0
150
36708_S2_L001_R1_001 (paired) trimmed (paired) (Reads)
3 141 055 reads
0
150
34827_S38_L001_R1_001 (paired) trimmed (paired) (Reads)
992 570 reads
0
150
34870_S39_L001_R1_001 (paired) trimmed (paired) (Reads)
1 017 816 reads
0
150
36348_S1_L001_R1_001 (paired) trimmed (paired) (Reads)
2 833 267 reads
0
150
36293_S37_L001_R1_001 (paired) trimmed (paired) (Reads)
1 248 695 reads
0
150
36493_S3_L001_R1_001 (paired) trimmed (paired) (Reads)
1 822 023 reads
0

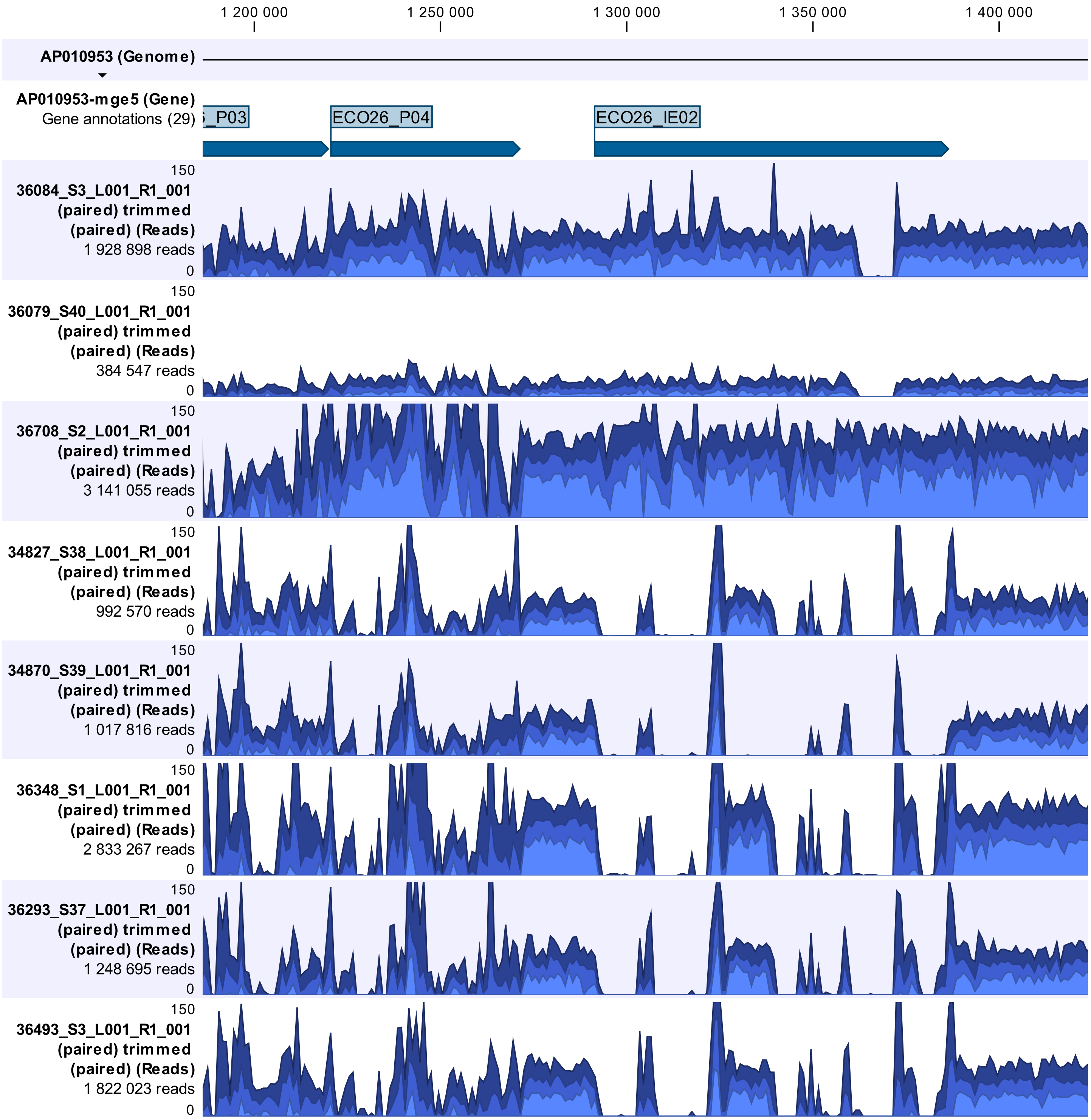

1 200 000
1 250 000
1 300 000
1 350 000
1 400 000
AP010953 (Genome)
AP010953-mge5 (Gene)
Gene annotations (29)
_P03
ECO26_P04
ECO26_IE02
150
36084_S3_L001_R1_001 (paired) trimmed (paired) (Reads)
1 928 898 reads
0
150
36079_S40_L001_R1_001 (paired) trimmed (paired) (Reads)
384 547 reads
0
150
36708_S2_L001_R1_001 (paired) trimmed (paired) (Reads)
3 141 055 reads
0
150
34827_S38_L001_R1_001 (paired) trimmed (paired) (Reads)
992 570 reads
0
150
34870_S39_L001_R1_001 (paired) trimmed (paired) (Reads)
1 017 816 reads
0
150
36348_S1_L001_R1_001 (paired) trimmed (paired) (Reads)
2 833 267 reads
0
150
36293_S37_L001_R1_001 (paired) trimmed (paired) (Reads)
1 248 695 reads
0
150
36493_S3_L001_R1_001 (paired) trimmed (paired) (Reads)
1 822 023 reads
0

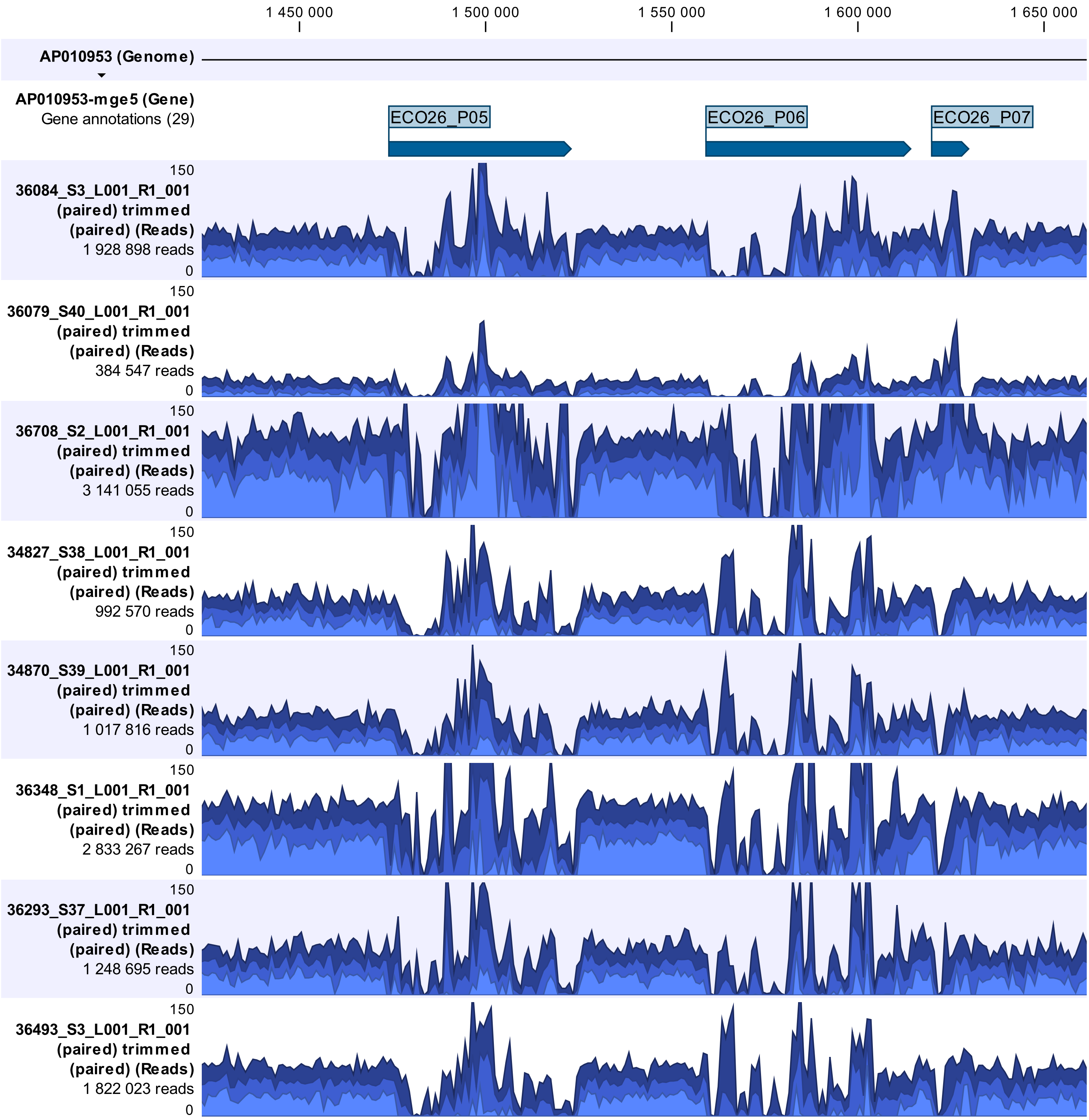
1 450 000
1 500 000
1 550 000
1 600 000
1 650 000
AP010953 (Genome)
AP010953-mge5 (Gene)
Gene annotations (29)
ECO26_P05
ECO26_P06
ECO26_P07
150
36084_S3_L001_R1_001 (paired) trimmed (paired) (Reads)
1 928 898 reads
0
150
36079_S40_L001_R1_001 (paired) trimmed (paired) (Reads)
384 547 reads
0
150
36708_S2_L001_R1_001 (paired) trimmed (paired) (Reads)
3 141 055 reads
0
150
34827_S38_L001_R1_001 (paired) trimmed (paired) (Reads)
992 570 reads
0
150
34870_S39_L001_R1_001 (paired) trimmed (paired) (Reads)
1 017 816 reads
0
150
36348_S1_L001_R1_001 (paired) trimmed (paired) (Reads)
2 833 267 reads
0
150
36293_S37_L001_R1_001 (paired) trimmed (paired) (Reads)
1 248 695 reads
0
150
36493_S3_L001_R1_001 (paired) trimmed (paired) (Reads)
1 822 023 reads
0

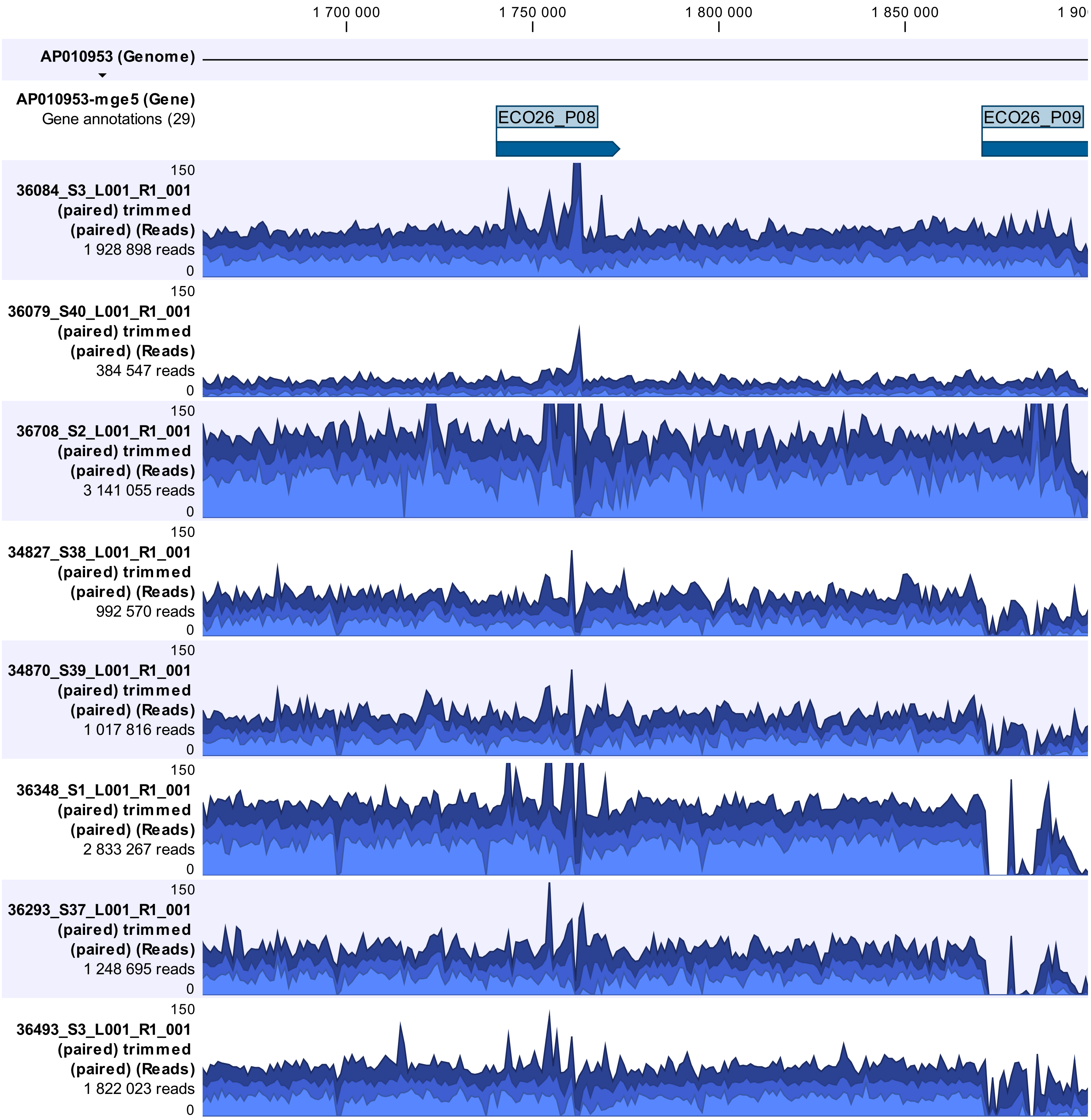
1 700 000
1 750 000
1 800 000
1 850 000
AP010953 (Genome)
AP010953-mge5 (Gene)
Gene annotations (29)
ECO26_P08
ECO26_P09
150
36084_S3_L001_R1_001 (paired) trimmed (paired) (Reads)
1 928 898 reads
0
150
36079_S40_L001_R1_001 (paired) trimmed (paired) (Reads)
384 547 reads
0
150
36708_S2_L001_R1_001 (paired) trimmed (paired) (Reads)
3 141 055 reads
0
150
34827_S38_L001_R1_001 (paired) trimmed (paired) (Reads)
992 570 reads
0
150
34870_S39_L001_R1_001 (paired) trimmed (paired) (Reads)
1 017 816 reads
0
150
36348_S1_L001_R1_001 (paired) trimmed (paired) (Reads)
2 833 267 reads
0
150
36293_S37_L001_R1_001 (paired) trimmed (paired) (Reads)
1 248 695 reads
0
150
36493_S3_L001_R1_001 (paired) trimmed (paired) (Reads)
1 822 023 reads
0

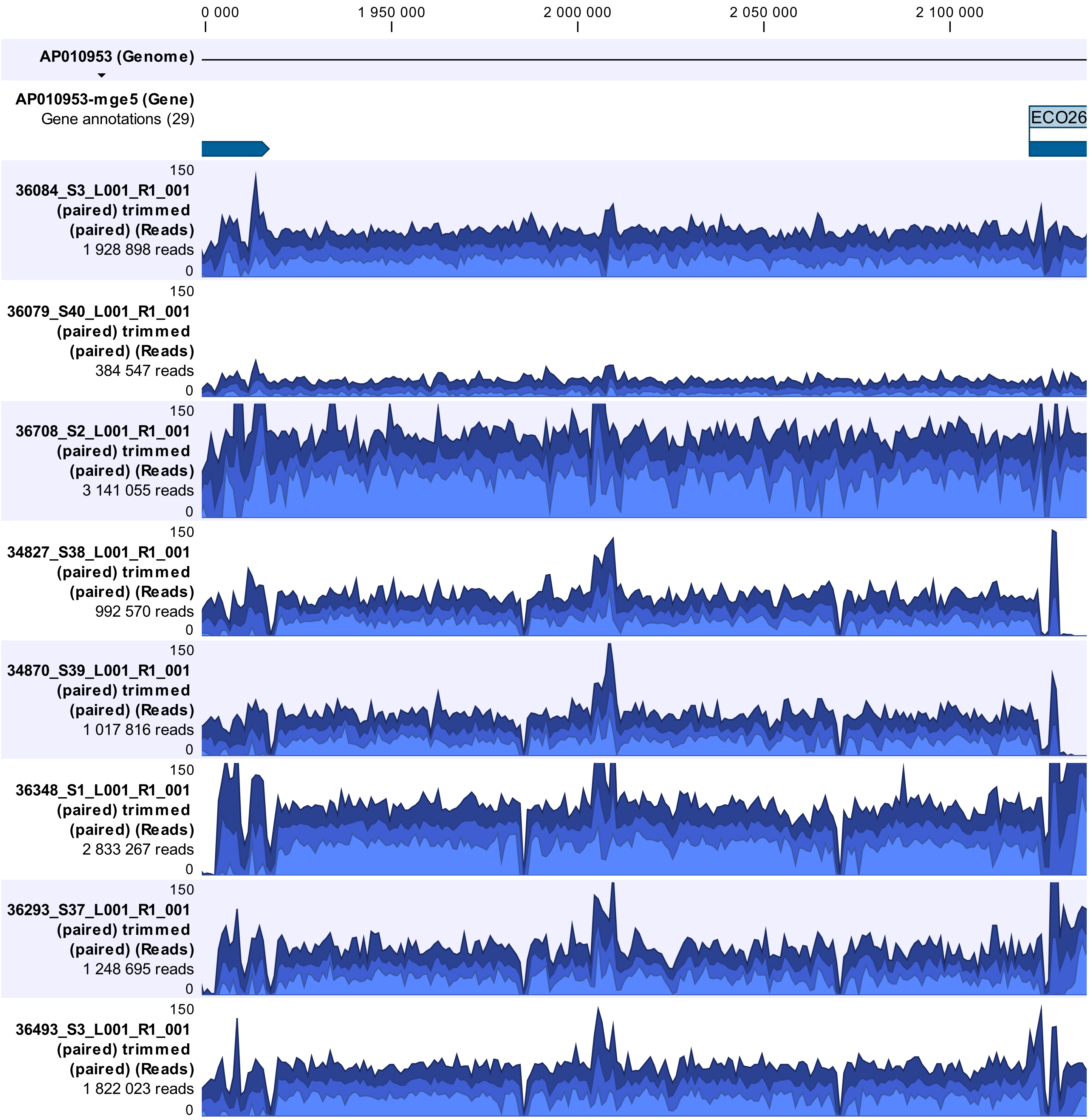

0 000
1 950 000
2 000 000
2 050 000
2 100 000
AP010953 (Genome)
AP010953-mge5 (Gene)
Gene annotations (29)
ECO26
150
36084_S3_L001_R1_001 (paired) trimmed (paired) (Reads)
1 928 898 reads
0
150
36079_S40_L001_R1_001 (paired) trimmed (paired) (Reads)
384 547 reads
0
150
36708_S2_L001_R1_001 (paired) trimmed (paired) (Reads)
3 141 055 reads
0
150
34827_S38_L001_R1_001 (paired) trimmed (paired) (Reads)
992 570 reads
0
150
34870_S39_L001_R1_001 (paired) trimmed (paired) (Reads)
1 017 816 reads
0
150
36348_S1_L001_R1_001 (paired) trimmed (paired) (Reads)
2 833 267 reads
0
150
36293_S37_L001_R1_001 (paired) trimmed (paired) (Reads)
1 248 695 reads
0
150
36493_S3_L001_R1_001 (paired) trimmed (paired) (Reads)
1 822 023 reads
0

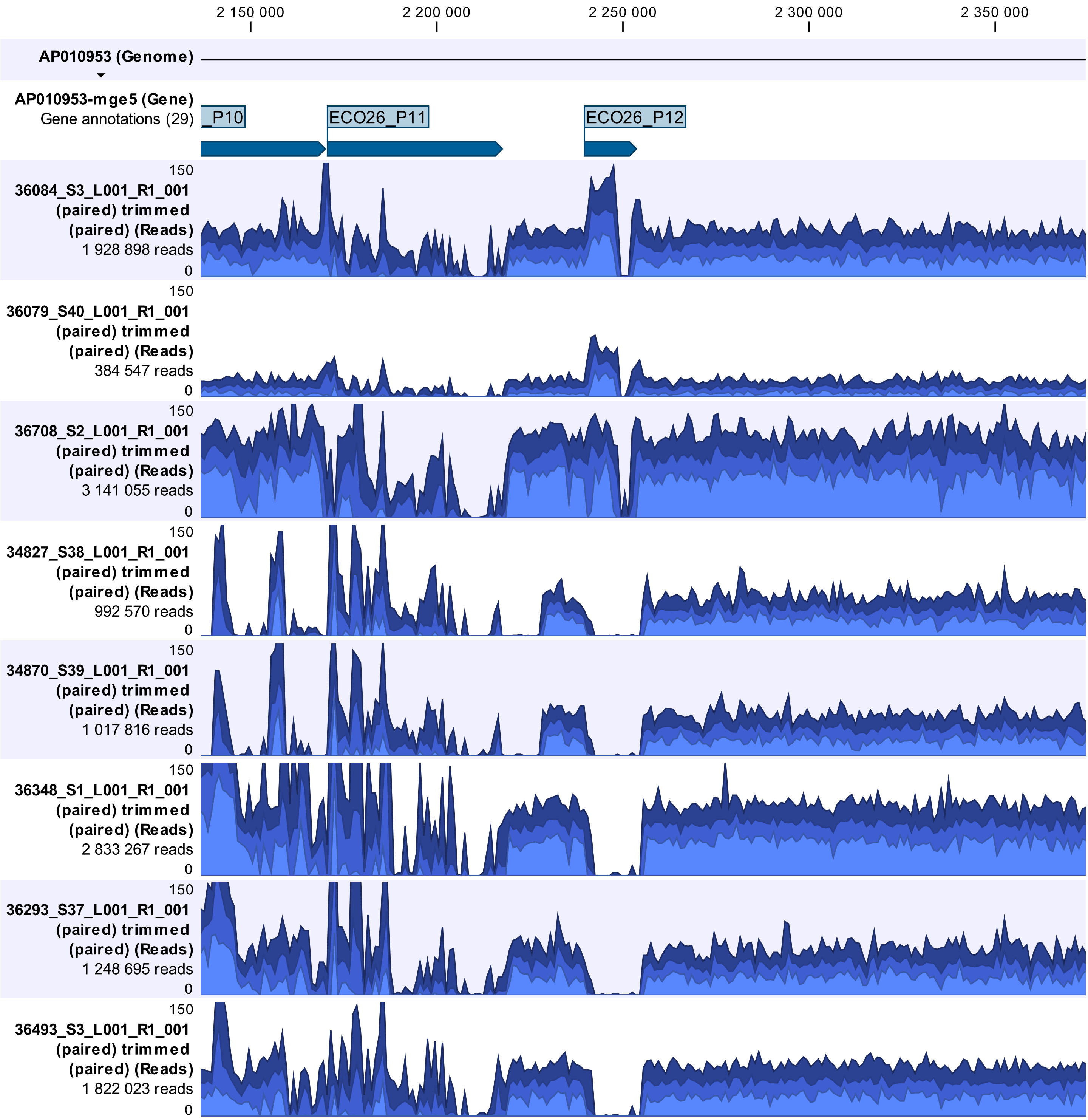

2 150 000
2 200 000
2 250 000
2 300 000
2 350 000
AP010953 (Genome)
AP010953-mge5 (Gene)
Gene annotations (29)
_P10
ECO26_P11
ECO26_P12
150
36084_S3_L001_R1_001 (paired) trimmed (paired) (Reads)
1 928 898 reads
0
150
36079_S40_L001_R1_001 (paired) trimmed (paired) (Reads)
384 547 reads
0
150
36708_S2_L001_R1_001 (paired) trimmed (paired) (Reads)
3 141 055 reads
0
150
34827_S38_L001_R1_001 (paired) trimmed (paired) (Reads)
992 570 reads
0
150
34870_S39_L001_R1_001 (paired) trimmed (paired) (Reads)
1 017 816 reads
0
150
36348_S1_L001_R1_001 (paired) trimmed (paired) (Reads)
2 833 267 reads
0
150
36293_S37_L001_R1_001 (paired) trimmed (paired) (Reads)
1 248 695 reads
0
150
36493_S3_L001_R1_001 (paired) trimmed (paired) (Reads)
1 822 023 reads
0

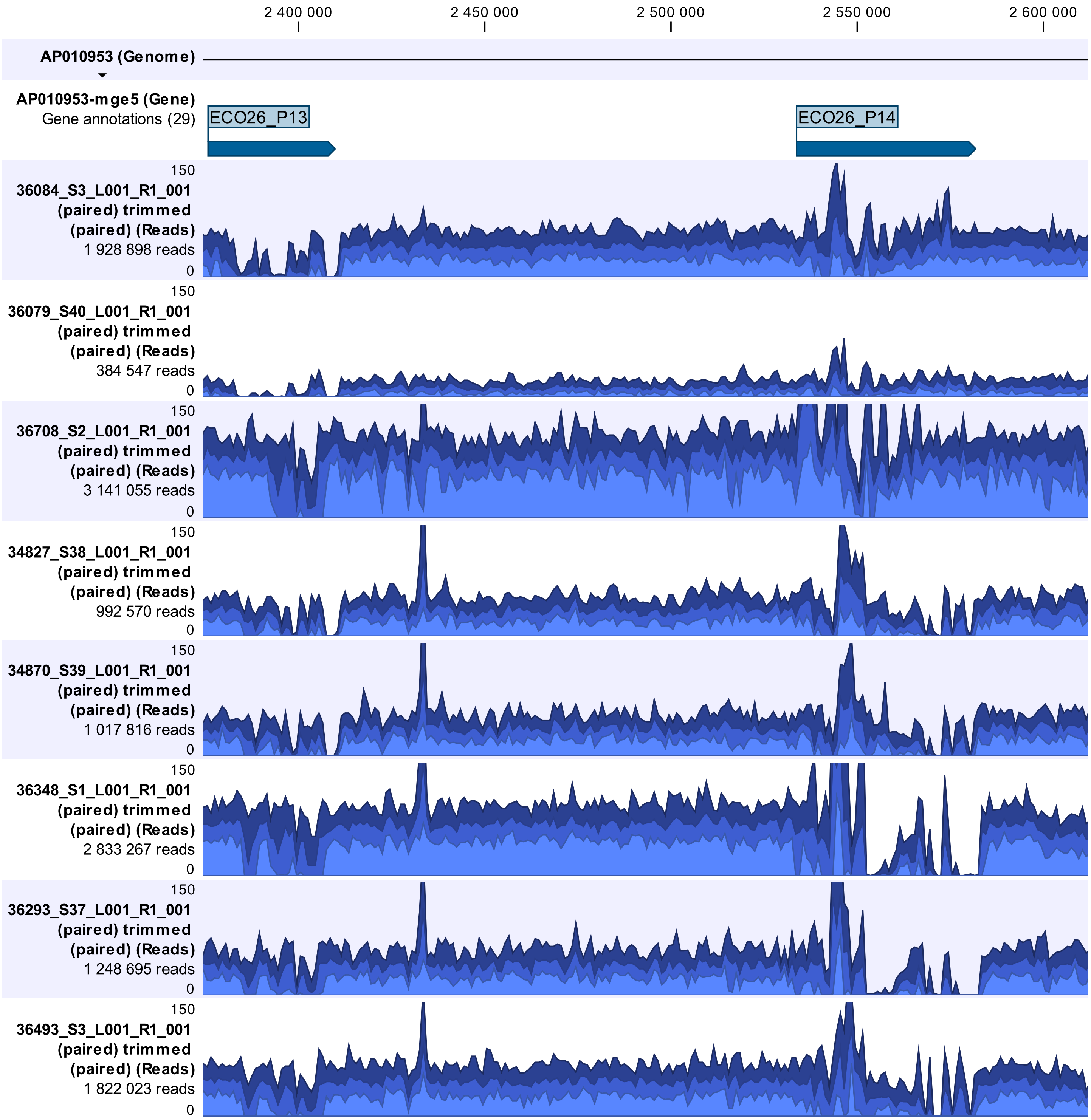
2 400 000
2 450 000
2 500 000
2 550 000
2 600 000
AP010953 (Genome)
AP010953-mge5 (Gene)
Gene annotations (29)
ECO26_P13
ECO26_P14
150
36084_S3_L001_R1_001 (paired) trimmed (paired) (Reads)
1 928 898 reads
0
150
36079_S40_L001_R1_001 (paired) trimmed (paired) (Reads)
384 547 reads
0
150
36708_S2_L001_R1_001 (paired) trimmed (paired) (Reads)
3 141 055 reads
0
150
34827_S38_L001_R1_001 (paired) trimmed (paired) (Reads)
992 570 reads
0
150
34870_S39_L001_R1_001 (paired) trimmed (paired) (Reads)
1 017 816 reads
0
150
36348_S1_L001_R1_001 (paired) trimmed (paired) (Reads)
2 833 267 reads
0
150
36293_S37_L001_R1_001 (paired) trimmed (paired) (Reads)
1 248 695 reads
0
150
36493_S3_L001_R1_001 (paired) trimmed (paired) (Reads)
1 822 023 reads
0

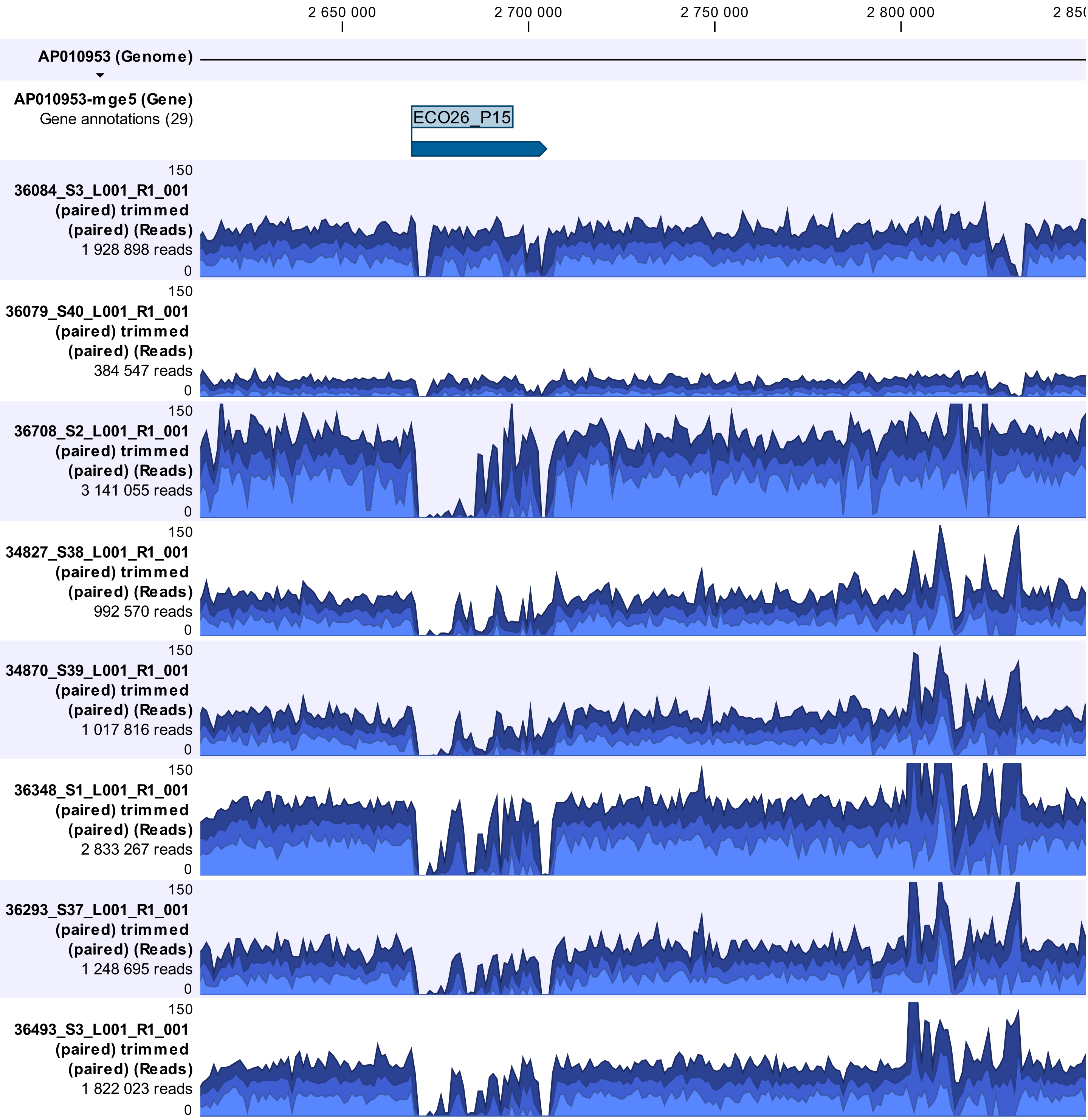

2 650 000
2 700 000
2 750 000
2 800 000
AP010953 (Genome)
AP010953-mge5 (Gene)
Gene annotations (29)
ECO26_P15
150
36084_S3_L001_R1_001 (paired) trimmed (paired) (Reads)
1 928 898 reads
0
150
36079_S40_L001_R1_001 (paired) trimmed (paired) (Reads)
384 547 reads
0
150
36708_S2_L001_R1_001 (paired) trimmed (paired) (Reads)
3 141 055 reads
0
150
34827_S38_L001_R1_001 (paired) trimmed (paired) (Reads)
992 570 reads
0
150
34870_S39_L001_R1_001 (paired) trimmed (paired) (Reads)
1 017 816 reads
0
150
36348_S1_L001_R1_001 (paired) trimmed (paired) (Reads)
2 833 267 reads
0
150
36293_S37_L001_R1_001 (paired) trimmed (paired) (Reads)
1 248 695 reads
0
150
36493_S3_L001_R1_001 (paired) trimmed (paired) (Reads)
1 822 023 reads
0

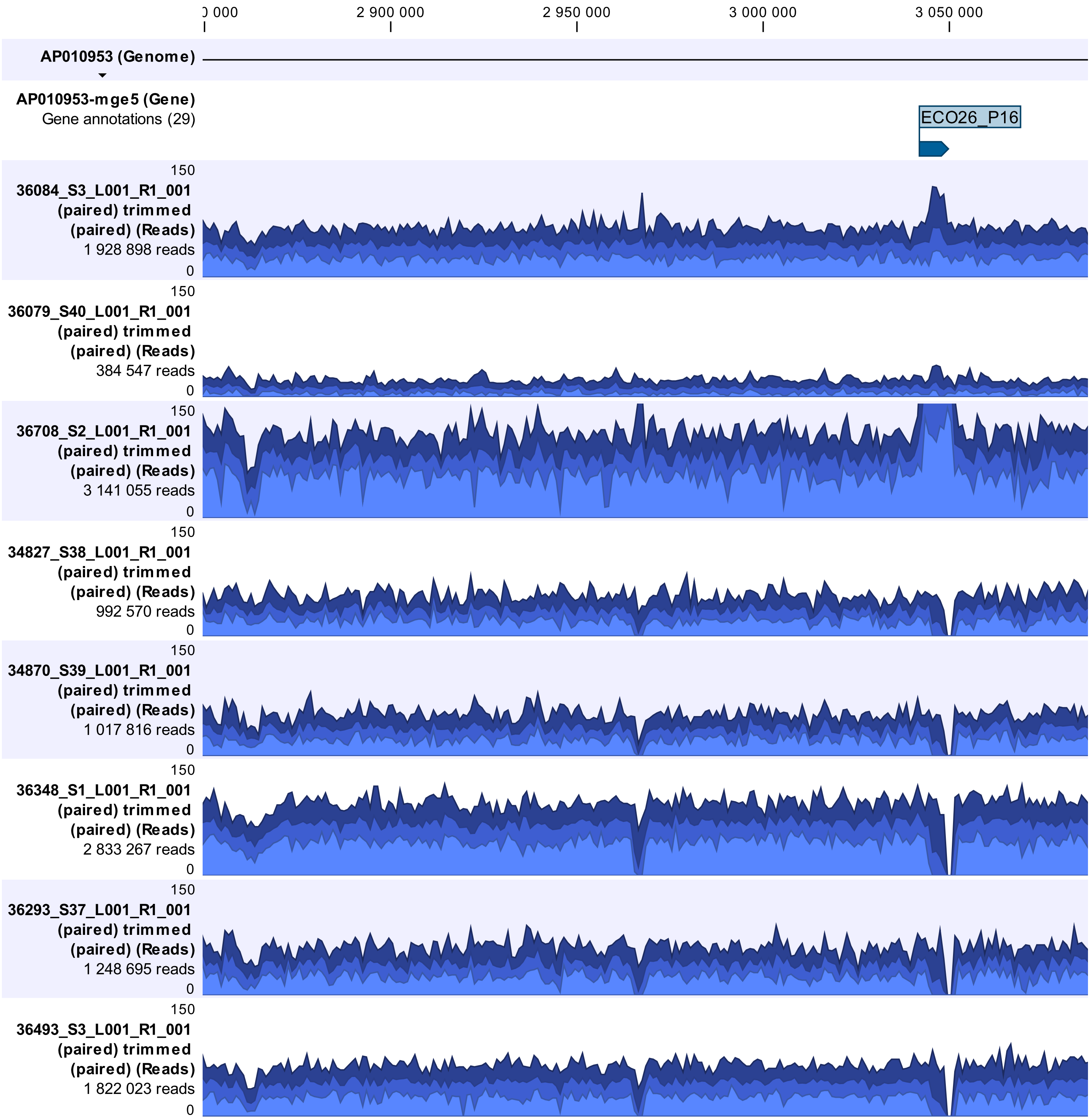
0 000
2 900 000
2 950 000
3 000 000
3 050 000
AP010953 (Genome)
AP010953-mge5 (Gene)
Gene annotations (29)
ECO26_P16
150
36084_S3_L001_R1_001 (paired) trimmed (paired) (Reads)
1 928 898 reads
0
150
36079_S40_L001_R1_001 (paired) trimmed (paired) (Reads)
384 547 reads
0
150
36708_S2_L001_R1_001 (paired) trimmed (paired) (Reads)
3 141 055 reads
0
150
34827_S38_L001_R1_001 (paired) trimmed (paired) (Reads)
992 570 reads
0
150
34870_S39_L001_R1_001 (paired) trimmed (paired) (Reads)
1 017 816 reads
0
150
36348_S1_L001_R1_001 (paired) trimmed (paired) (Reads)
2 833 267 reads
0
150
36293_S37_L001_R1_001 (paired) trimmed (paired) (Reads)
1 248 695 reads
0
150
36493_S3_L001_R1_001 (paired) trimmed (paired) (Reads)
1 822 023 reads
0

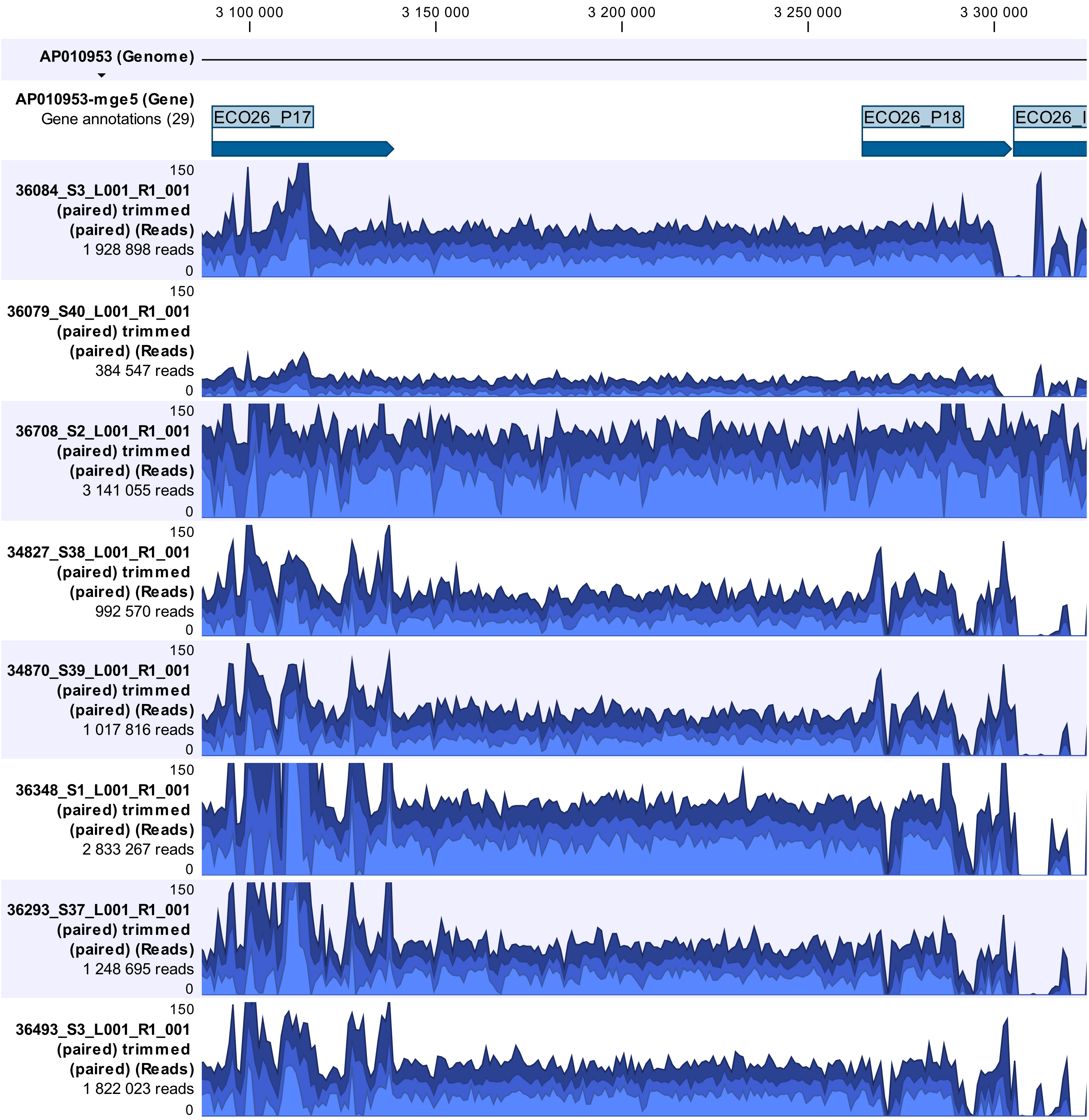

3 100 000
3 150 000
3 200 000
3 250 000
3 300 000
AP010953 (Genome)
AP010953-mge5 (Gene)
Gene annotations (29)
ECO26_P17
ECO26_P18
ECO26_I
150
36084_S3_L001_R1_001 (paired) trimmed (paired) (Reads)
1 928 898 reads
0
150
36079_S40_L001_R1_001 (paired) trimmed (paired) (Reads)
384 547 reads
0
150
36708_S2_L001_R1_001 (paired) trimmed (paired) (Reads)
3 141 055 reads
0
150
34827_S38_L001_R1_001 (paired) trimmed (paired) (Reads)
992 570 reads
0
150
34870_S39_L001_R1_001 (paired) trimmed (paired) (Reads)
1 017 816 reads
0
150
36348_S1_L001_R1_001 (paired) trimmed (paired) (Reads)
2 833 267 reads
0
150
36293_S37_L001_R1_001 (paired) trimmed (paired) (Reads)
1 248 695 reads
0
150
36493_S3_L001_R1_001 (paired) trimmed (paired) (Reads)
1 822 023 reads
0

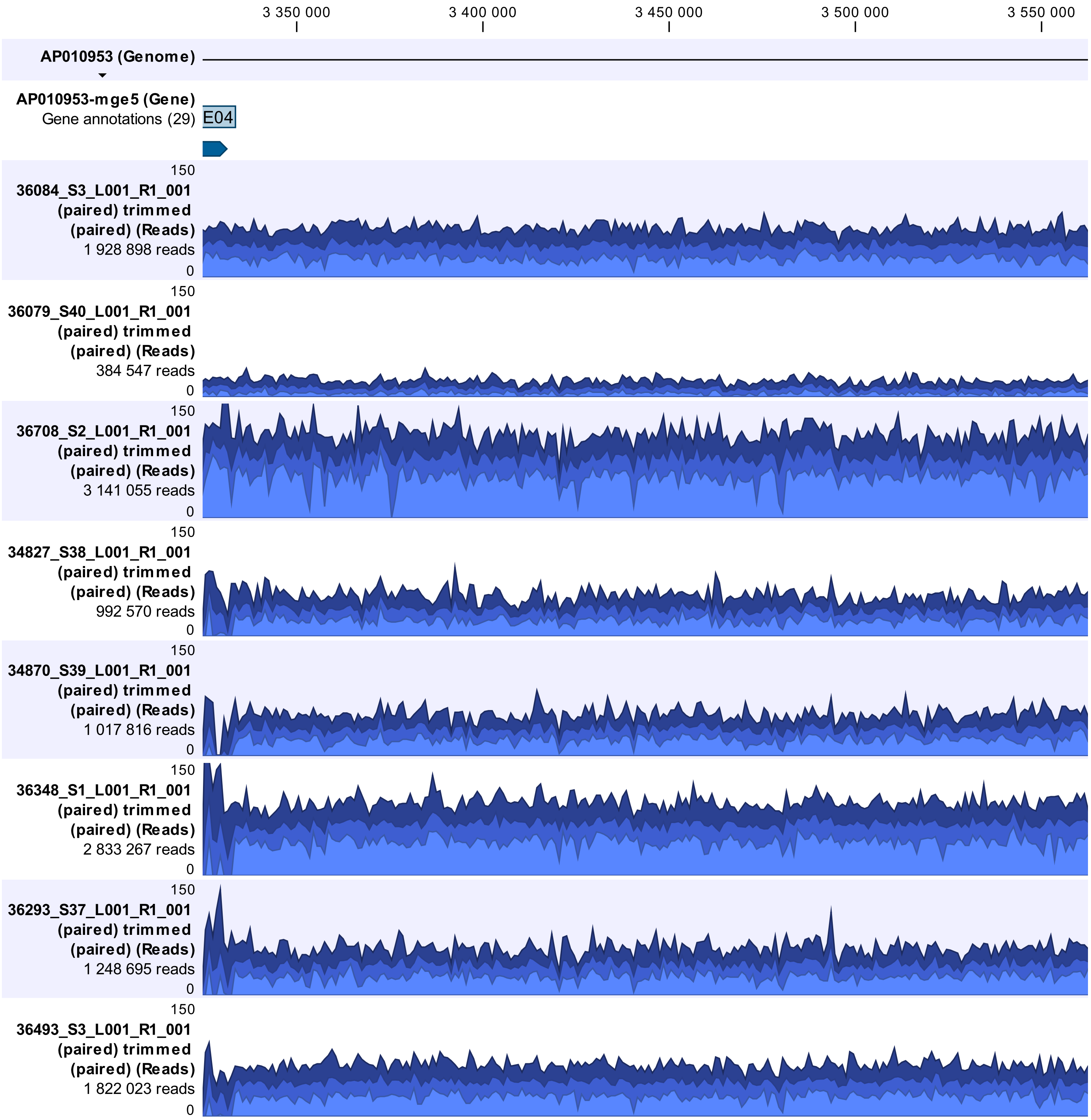
3 350 000
3 400 000
3 450 000
3 500 000
3 550 000
AP010953 (Genome)
AP010953-mge5 (Gene)
Gene annotations (29)
E04
150
36084_S3_L001_R1_001 (paired) trimmed (paired) (Reads)
1 928 898 reads
0
150
36079_S40_L001_R1_001 (paired) trimmed (paired) (Reads)
384 547 reads
0
150
36708_S2_L001_R1_001 (paired) trimmed (paired) (Reads)
3 141 055 reads
0
150
34827_S38_L001_R1_001 (paired) trimmed (paired) (Reads)
992 570 reads
0
150
34870_S39_L001_R1_001 (paired) trimmed (paired) (Reads)
1 017 816 reads
0
150
36348_S1_L001_R1_001 (paired) trimmed (paired) (Reads)
2 833 267 reads
0
150
36293_S37_L001_R1_001 (paired) trimmed (paired) (Reads)
1 248 695 reads
0
150
36493_S3_L001_R1_001 (paired) trimmed (paired) (Reads)
1 822 023 reads
0

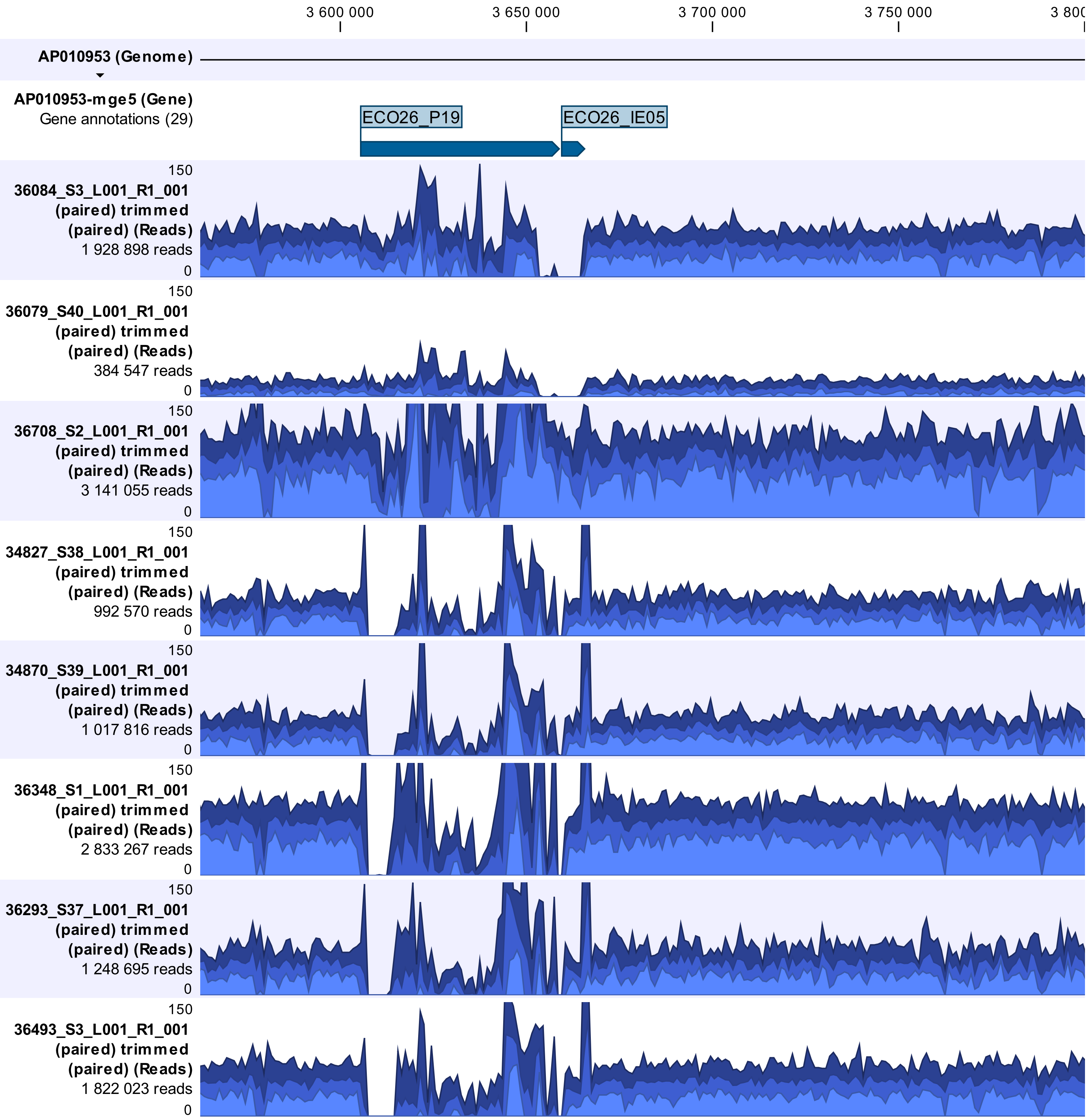
3 600 000
3 650 000
3 700 000
3 750 000
AP010953 (Genome)
AP010953-mge5 (Gene)
Gene annotations (29)
ECO26_P19
ECO26_IE05
36084_S3_L001_R1_001 (paired) trimmed (paired) (Reads)
1 928 898 reads
36079_S40_L001_R1_001 (paired) trimmed (paired) (Reads)
384 547 reads
36708_S2_L001_R1_001 (paired) trimmed (paired) (Reads)
3 141 055 reads
34827_S38_L001_R1_001 (paired) trimmed (paired) (Reads)
992 570 reads
34870_S39_L001_R1_001 (paired) trimmed (paired) (Reads)
1 017 816 reads
36348_S1_L001_R1_001 (paired) trimmed (paired) (Reads)
2 833 267 reads
36293_S37_L001_R1_001 (paired) trimmed (paired) (Reads)
1 248 695 reads
36493_S3_L001_R1_001 (paired) trimmed (paired) (Reads)
1 822 023 reads
150
0

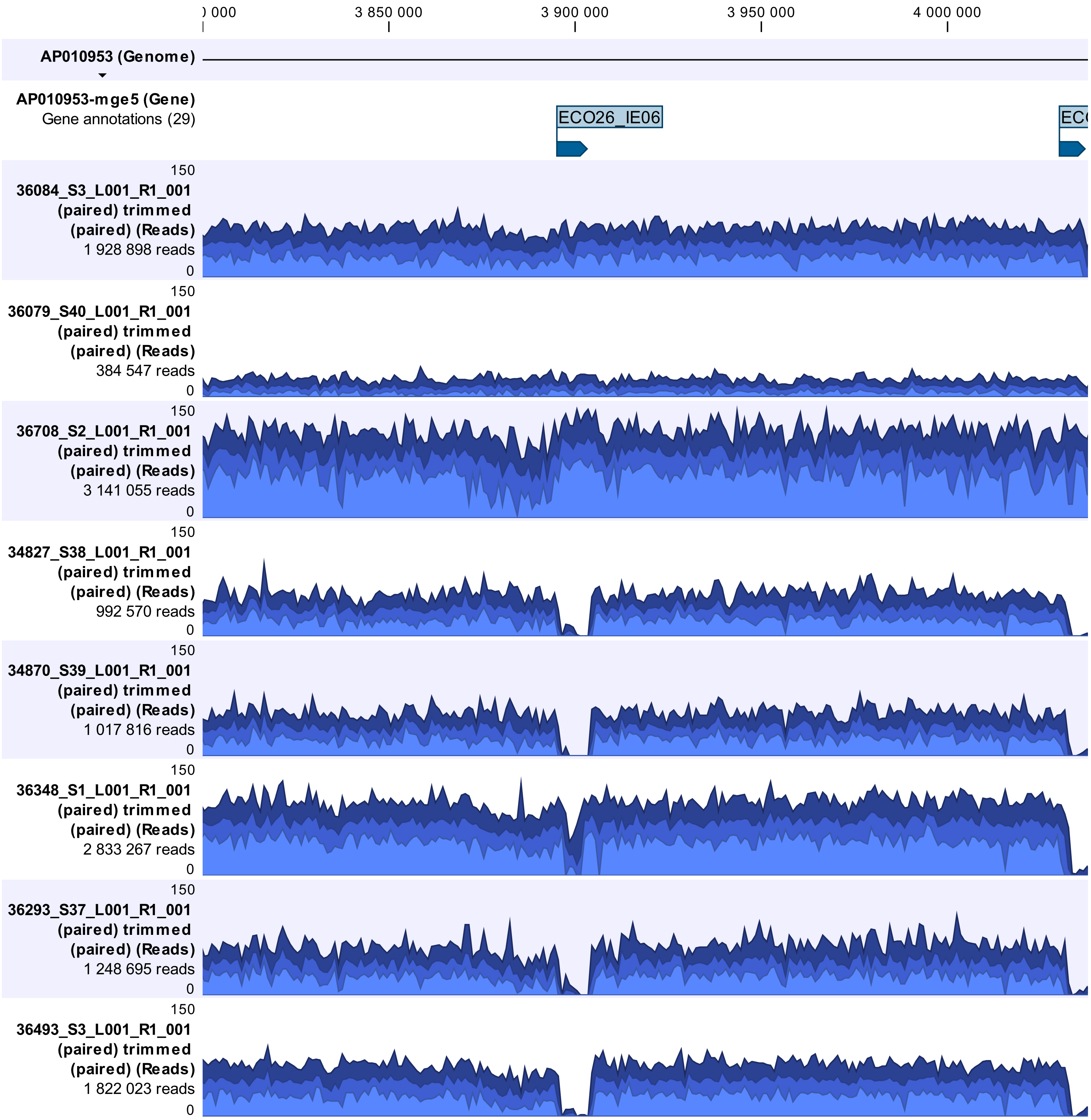

) 000
3 850 000
3 900 000
3 950 000
4 000 000
AP010953 (Genome)
AP010953-mge5 (Gene)
Gene annotations (29)
ECO26_IE06
EC
150
36084_S3_L001_R1_001 (paired) trimmed (paired) (Reads)
1 928 898 reads
0
150
36079_S40_L001_R1_001 (paired) trimmed (paired) (Reads)
384 547 reads
0
150
36708_S2_L001_R1_001 (paired) trimmed (paired) (Reads)
3 141 055 reads
0
150
34827_S38_L001_R1_001 (paired) trimmed (paired) (Reads)
992 570 reads
0
150
34870_S39_L001_R1_001 (paired) trimmed (paired) (Reads)
1 017 816 reads
0
150
36348_S1_L001_R1_001 (paired) trimmed (paired) (Reads)
2 833 267 reads
0
150
36293_S37_L001_R1_001 (paired) trimmed (paired) (Reads)
1 248 695 reads
0
150
36493_S3_L001_R1_001 (paired) trimmed (paired) (Reads)
1 822 023 reads
0

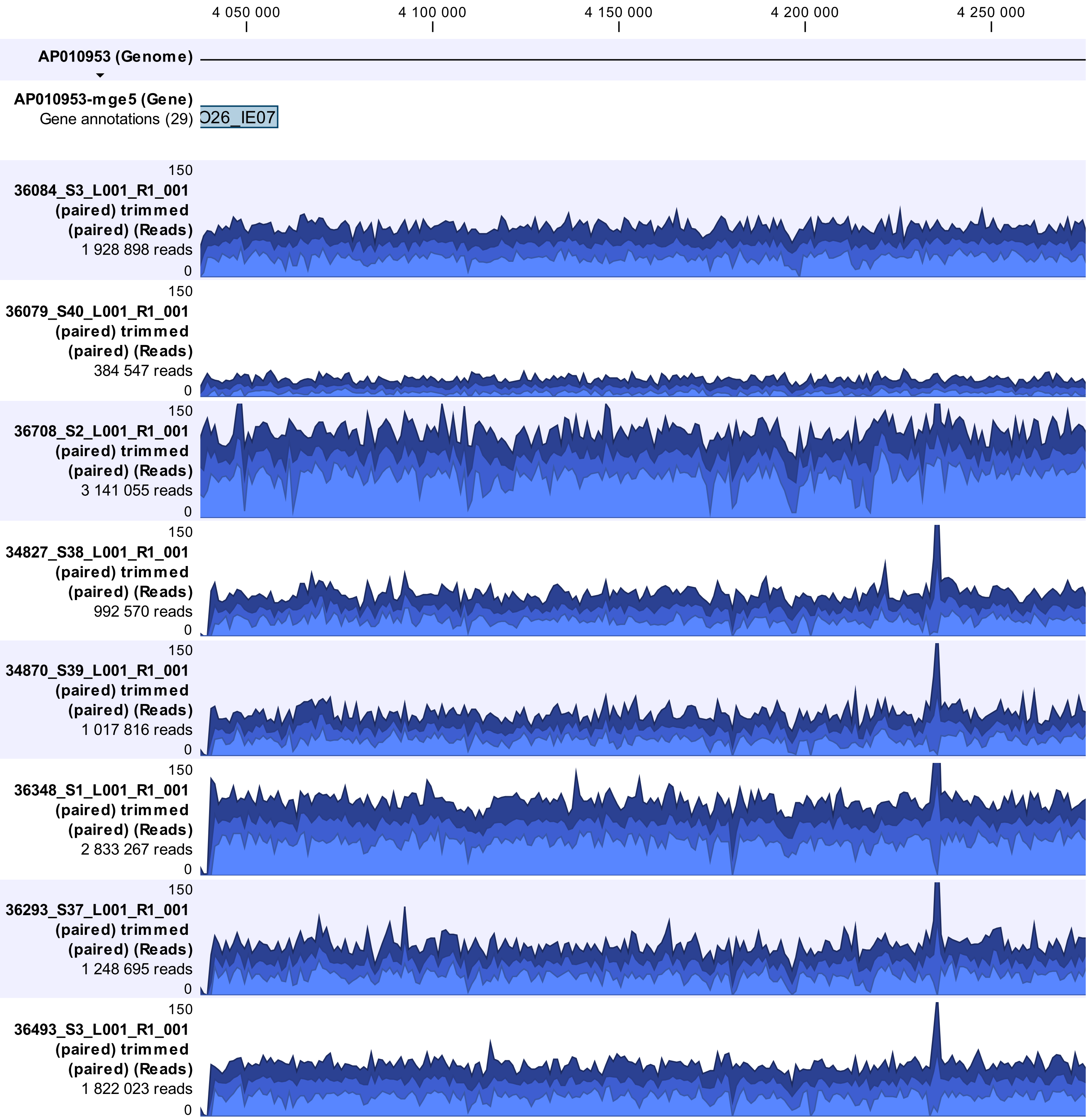
4 050 000
4 100 000
4 150 000
4 200 000
4 250 000
AP010953 (Genome)
AP010953-mge5 (Gene)
Gene annotations (29)
O26_IE07
150
36084_S3_L001_R1_001 (paired) trimmed (paired) (Reads)
1 928 898 reads
0
150
36079_S40_L001_R1_001 (paired) trimmed (paired) (Reads)
384 547 reads
0
150
36708_S2_L001_R1_001 (paired) trimmed (paired) (Reads)
3 141 055 reads
0
150
34827_S38_L001_R1_001 (paired) trimmed (paired) (Reads)
992 570 reads
0
150
34870_S39_L001_R1_001 (paired) trimmed (paired) (Reads)
1 017 816 reads
0
150
36348_S1_L001_R1_001 (paired) trimmed (paired) (Reads)
2 833 267 reads
0
150
36293_S37_L001_R1_001 (paired) trimmed (paired) (Reads)
1 248 695 reads
0
150
36493_S3_L001_R1_001 (paired) trimmed (paired) (Reads)
1 822 023 reads
0

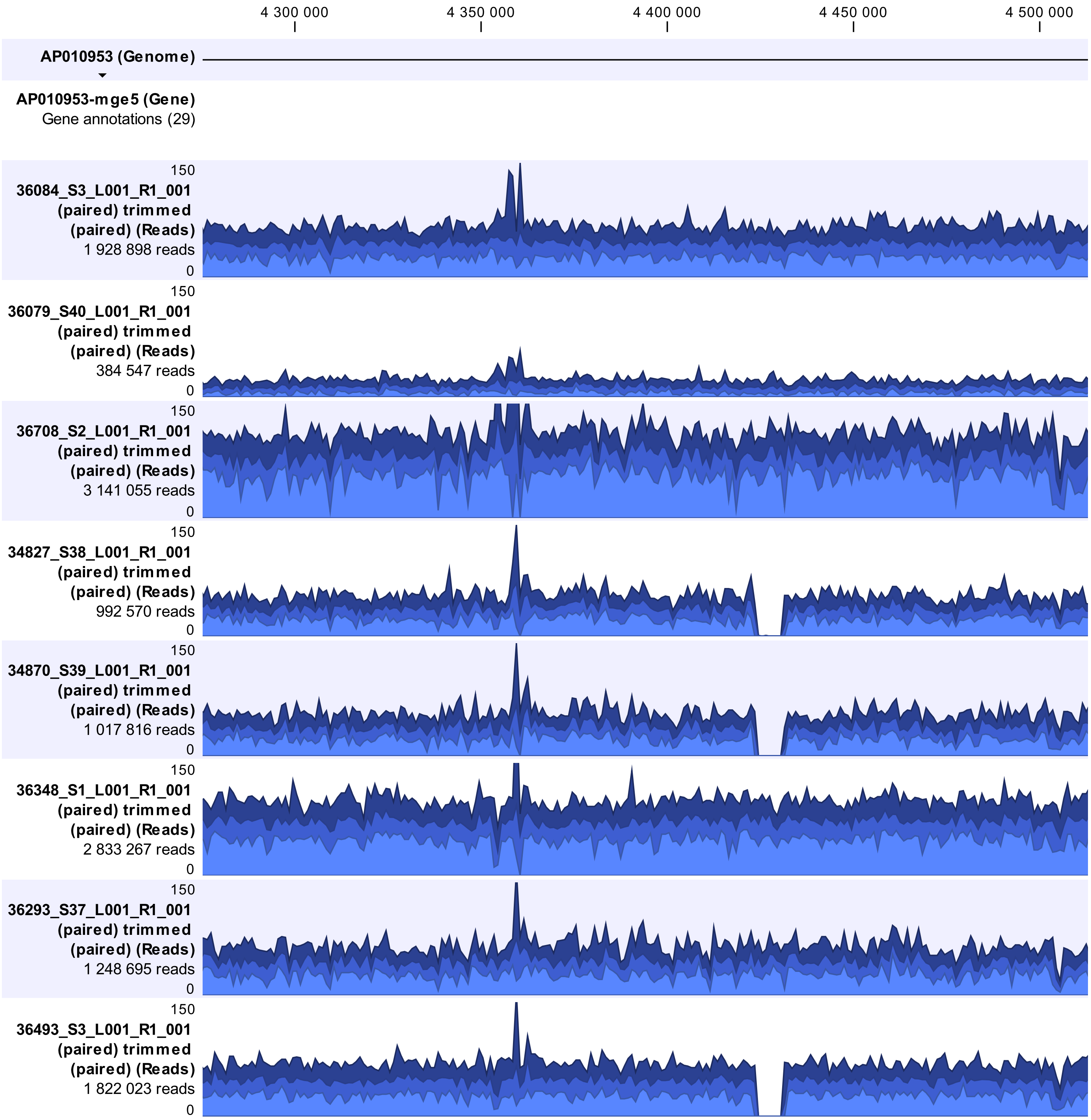
4 300 000
4 350 000
4 400 000
4 450 000
4 500 000
AP010953 (Genome)
AP010953-mge5 (Gene)
Gene annotations (29)
150
36084_S3_L001_R1_001 (paired) trimmed (paired) (Reads)
1 928 898 reads
0
150
36079_S40_L001_R1_001 (paired) trimmed (paired) (Reads)
384 547 reads
0
150
36708_S2_L001_R1_001 (paired) trimmed (paired) (Reads)
3 141 055 reads
0
150
34827_S38_L001_R1_001 (paired) trimmed (paired) (Reads)
992 570 reads
0
150
34870_S39_L001_R1_001 (paired) trimmed (paired) (Reads)
1 017 816 reads
0
150
36348_S1_L001_R1_001 (paired) trimmed (paired) (Reads)
2 833 267 reads
0
150
36293_S37_L001_R1_001 (paired) trimmed (paired) (Reads)
1 248 695 reads
0
150
36493_S3_L001_R1_001 (paired) trimmed (paired) (Reads)
1 822 023 reads
0

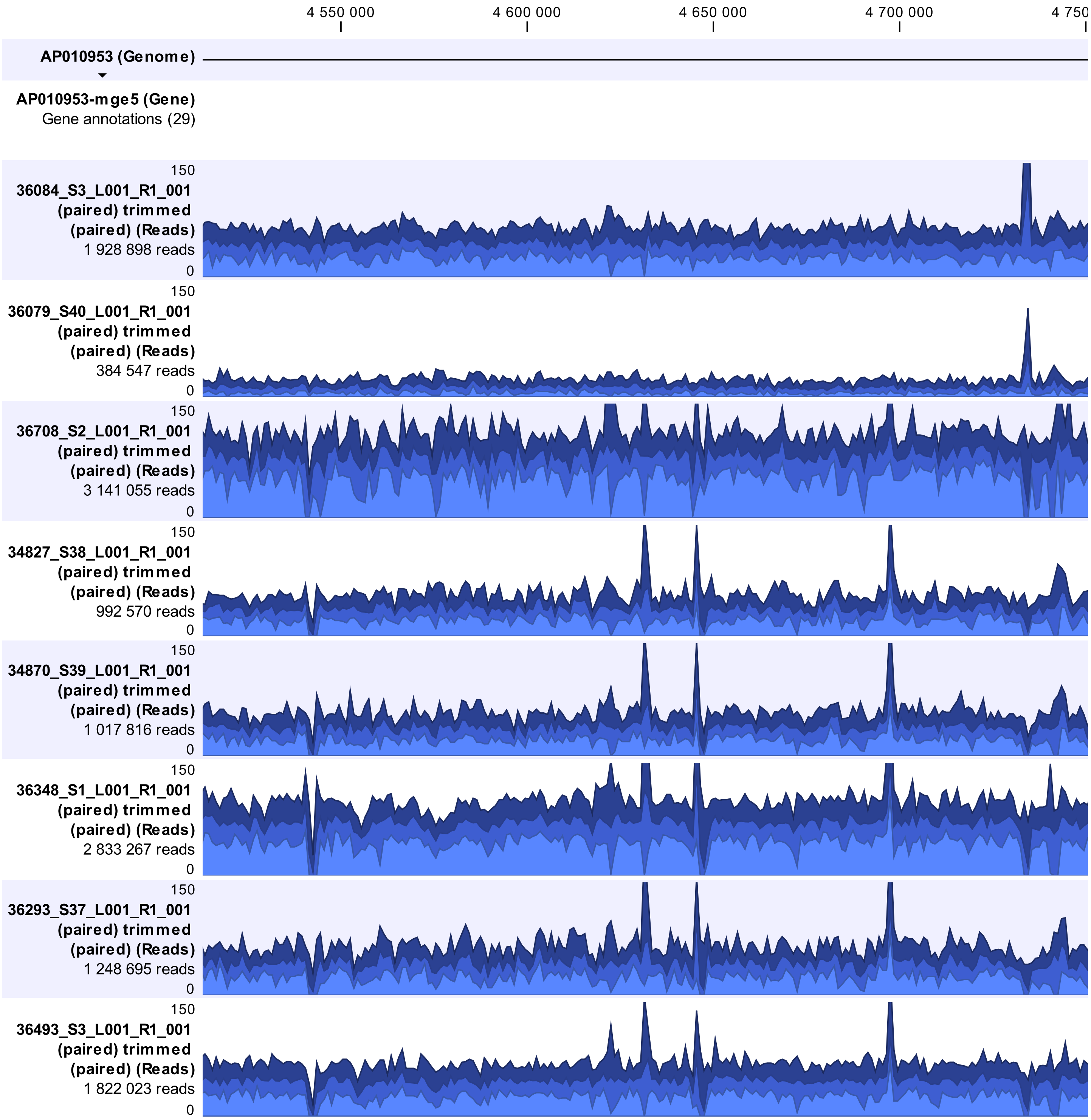
4 550 000
4 600 000
4 650 000
4 700 000
4 750
AP010953 (Genome)
AP010953-mge5 (Gene)
Gene annotations (29)
150
36084_S3_L001_R1_001 (paired) trimmed (paired) (Reads)
1 928 898 reads
0
150
36079_S40_L001_R1_001 (paired) trimmed (paired) (Reads)
384 547 reads
0
150
36708_S2_L001_R1_001 (paired) trimmed (paired) (Reads)
3 141 055 reads
0
150
34827_S38_L001_R1_001 (paired) trimmed (paired) (Reads)
992 570 reads
0
150
34870_S39_L001_R1_001 (paired) trimmed (paired) (Reads)
1 017 816 reads
0
150
36348_S1_L001_R1_001 (paired) trimmed (paired) (Reads)
2 833 267 reads
0
150
36293_S37_L001_R1_001 (paired) trimmed (paired) (Reads)
1 248 695 reads
0
150
36493_S3_L001_R1_001 (paired) trimmed (paired) (Reads)
1 822 023 reads
0

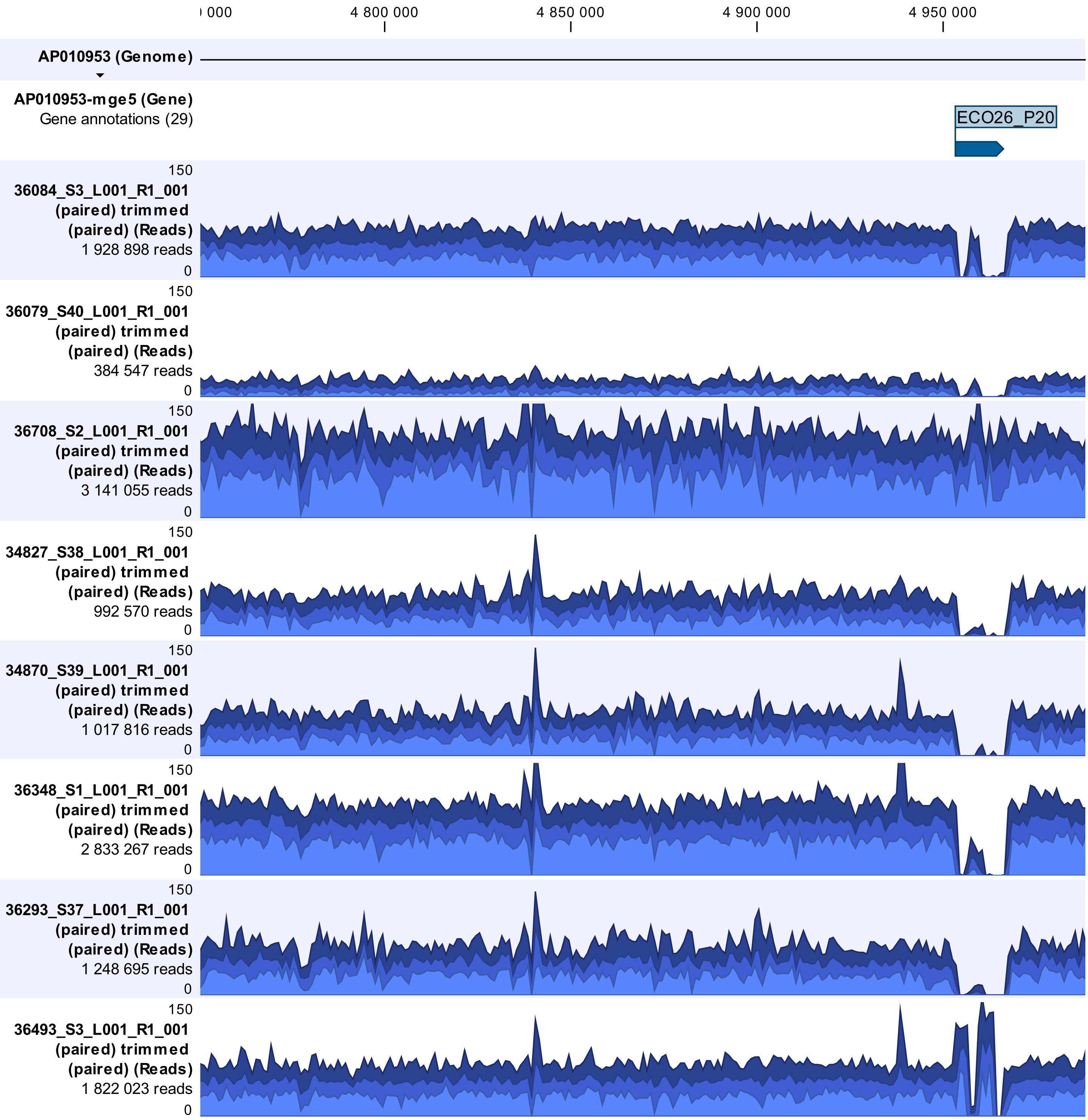

000
4 800 000
4 850 000
4 900 000
4 950 000
AP010953 (Genome)
AP010953-mge5 (Gene)
Gene annotations (29)
ECO26_P20
150
36084_S3_L001_R1_001 (paired) trimmed (paired) (Reads)
1 928 898 reads
0
150
36079_S40_L001_R1_001 (paired) trimmed (paired) (Reads)
384 547 reads
0
150
36708_S2_L001_R1_001 (paired) trimmed (paired) (Reads)
3 141 055 reads
0
150
34827_S38_L001_R1_001 (paired) trimmed (paired) (Reads)
992 570 reads
0
150
34870_S39_L001_R1_001 (paired) trimmed (paired) (Reads)
1 017 816 reads
0
150
36348_S1_L001_R1_001 (paired) trimmed (paired) (Reads)
2 833 267 reads
0
150
36293_S37_L001_R1_001 (paired) trimmed (paired) (Reads)
1 248 695 reads
0
150
36493_S3_L001_R1_001 (paired) trimmed (paired) (Reads)
1 822 023 reads
0

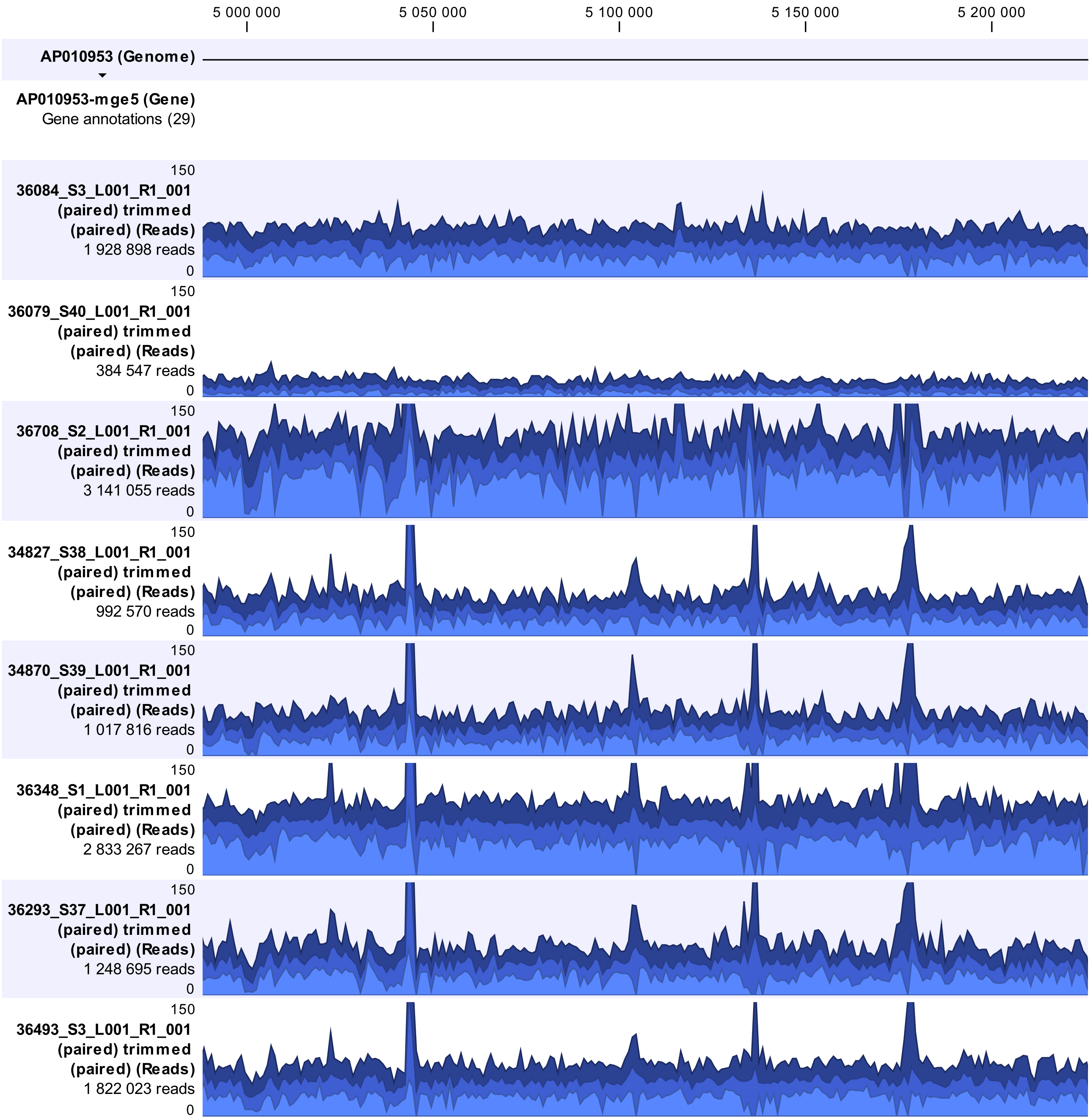
5 000 000
5 050 000
5 100 000
5 150 000
5 200 000
AP010953 (Genome)
AP010953-mge5 (Gene)
Gene annotations (29)
36084_S3_L001_R1_001 (paired) trimmed (paired) (Reads)
1 928 898 reads
36079_S40_L001_R1_001 (paired) trimmed (paired) (Reads)
384 547 reads
36708_S2_L001_R1_001 (paired) trimmed (paired) (Reads)
3 141 055 reads
34827_S38_L001_R1_001 (paired) trimmed (paired) (Reads)
992 570 reads
34870_S39_L001_R1_001 (paired) trimmed (paired) (Reads)
1 017 816 reads
36348_S1_L001_R1_001 (paired) trimmed (paired) (Reads)
2 833 267 reads
36293_S37_L001_R1_001 (paired) trimmed (paired) (Reads)
1 248 695 reads
36493_S3_L001_R1_001 (paired) trimmed (paired) (Reads)
1 822 023 reads
150
0

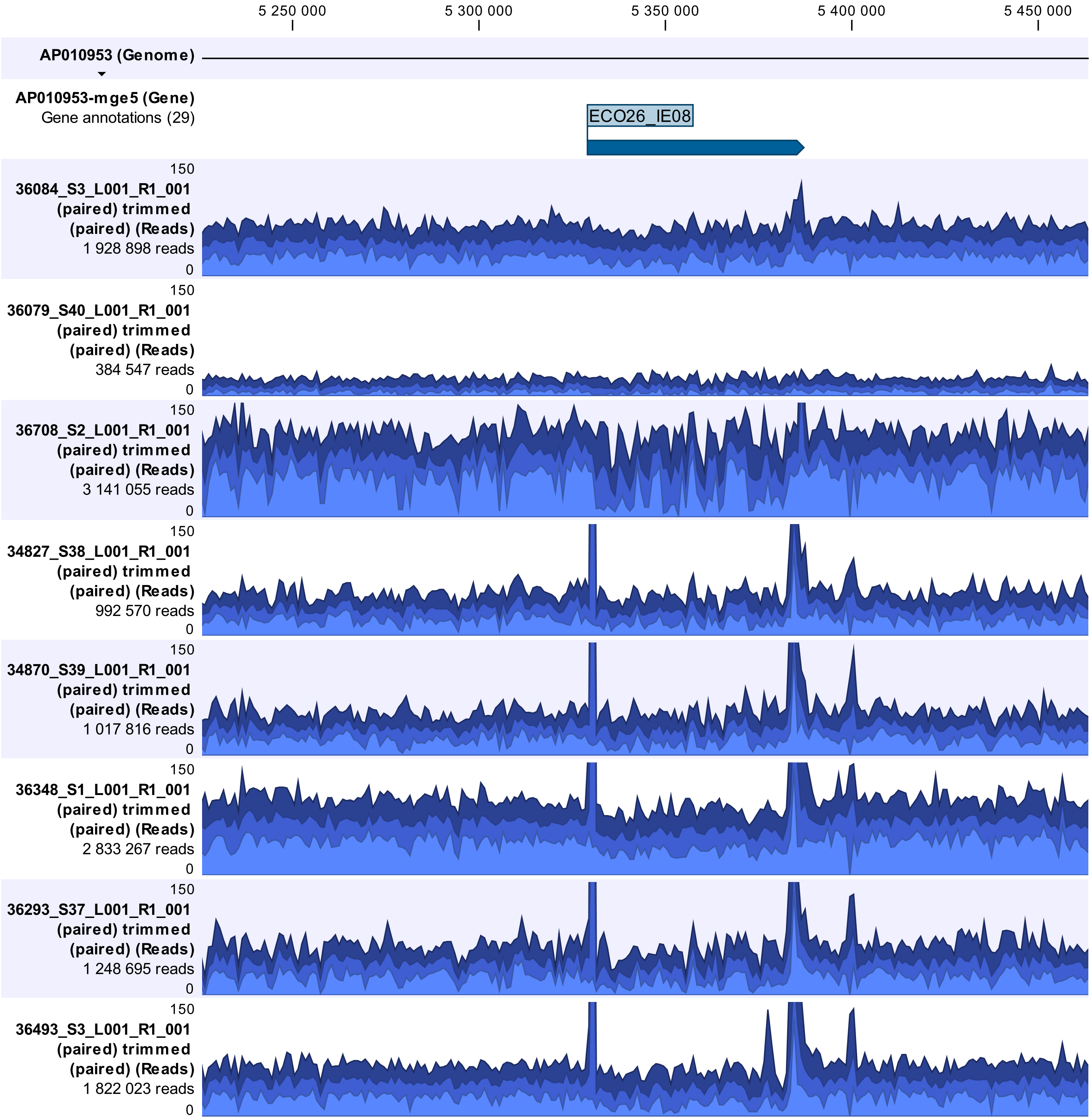
5 250 000
5 300 000
5 350 000
5 400 000
5 450 000
AP010953 (Genome)
AP010953-mge5 (Gene)
Gene annotations (29)
ECO26_IE08
150
36084_S3_L001_R1_001 (paired) trimmed (paired) (Reads)
1 928 898 reads
0
150
36079_S40_L001_R1_001 (paired) trimmed (paired) (Reads)
384 547 reads
0
150
36708_S2_L001_R1_001 (paired) trimmed (paired) (Reads)
3 141 055 reads
0
150
34827_S38_L001_R1_001 (paired) trimmed (paired) (Reads)
992 570 reads
0
150
34870_S39_L001_R1_001 (paired) trimmed (paired) (Reads)
1 017 816 reads
0
150
36348_S1_L001_R1_001 (paired) trimmed (paired) (Reads)
2 833 267 reads
0
150
36293_S37_L001_R1_001 (paired) trimmed (paired) (Reads)
1 248 695 reads
0
150
36493_S3_L001_R1_001 (paired) trimmed (paired) (Reads)
1 822 023 reads
0

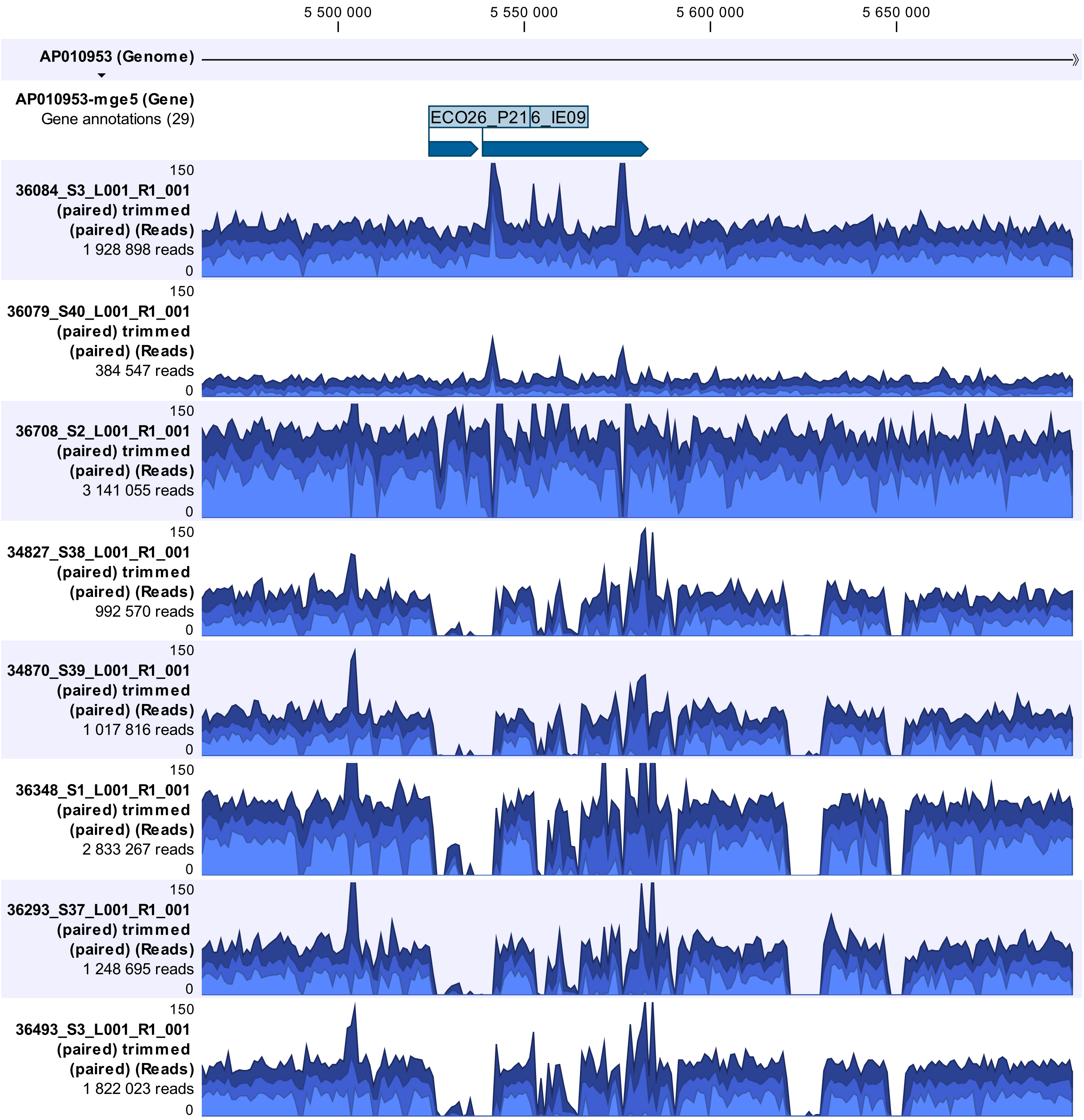

Figure S2: Graphical representation of Illumina short reads of the French isolates mapping on reference strain 11368 genome (AP010953). Mapping was performed with the CLC Genomics Workbench version 7.5.1 with the following parameters: References = AP010953, Masking mode = No masking, Mismatch cost = 2, Insertion cost = 3, Deletion cost = 3, Length fraction = 0,5, Similarity fraction = 0,8, Global alignment = No, Auto-detect paired distances = Yes, Non-specific match handling = Map randomly. The location of strain 11368 chromosomal mobile genetic elements (prophages and integrated elements) is indicated in the second row. The height of the graph is proportional to the mapping density. The scale for each isolate is indicated on the left side.
