# Supplementary material for: The Mobilome; A Major Contributor to Escherichia coli stx2-Positive O26:H11 Strains Intra-Serotype Diversity
Source: Front Microbiol. 2017 Sep 6;8:1625. doi: 10.3389/fmicb.2017.01625 (PMC5592225; doi:10.3389/fmicb.2017.01625)
Supplement: Supplementary file 16 [file Image7.PDF]

A

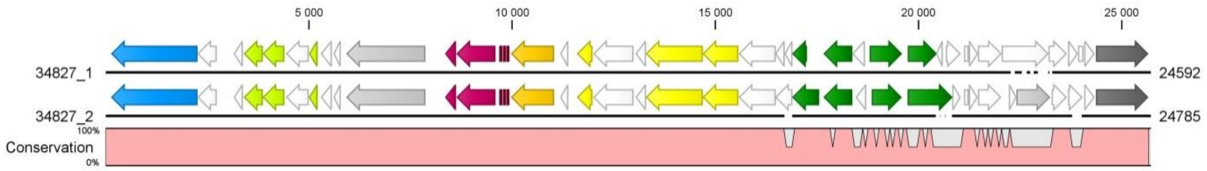

Sequence Identity Matrix

Input Alignment File: 34827_phage1-34827_phage2 alignment.fa

Seq->	34827_phage1-yecE	34827_phage2
34827_phage1-yecE	ID	0.861
34827_phage2	0.861	ID

Sequence Difference Count Matrix

Input Alignment File: 34827_phage1-34827_phage2 alignment.fa

Seq->	34827_phage1-yecE	34827_phage2
34827_phage1-yecE	ID	3570
34827_phage2	3570	ID

B

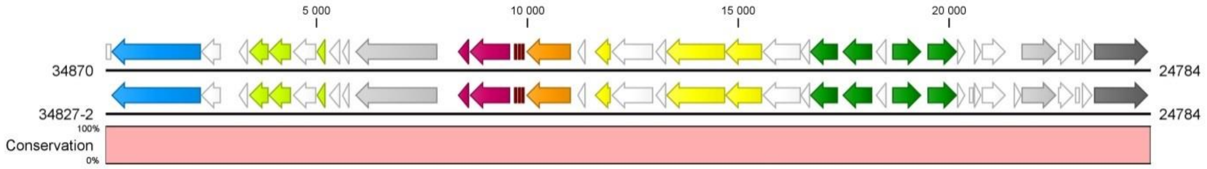

Sequence Identity Matrix

Input Alignment File: 34870-34827 alignment-short.fa

Seq->	34870	34827-2
34870	ID	0.998
34827-2	0.998	ID

Sequence Difference Count Matrix

Input Alignment File: 34870-34827 alignment-short.fa

Seq->	34870	34827-2
34870	ID	32
34827-2	32	ID

Figure S7: SNP-CC1 *Stx2a* prophages alignment. A. Comparison of the two *stx2a*-prophages in strain 34827. **B**. Comparison of the *stx2a*-prophages in strain 34870 and the homologous prophage in strain 34827. The sequences of the stx-prophages were aligned and visualized in CLC Genomics workbench (version 8.0.2). The length of the fragment aligned is indicated on the right. The nucleotide sequence is figured as a black line. Gaps in the alignment are indicated by breaks in the line. Nucleotide identity at each position between fragments is indicated as a plot below the alignment. The height of the line reflects how conserved that particular position is in the alignment. For example, 100% indicates that the nucleotide is conserved (identical) in 100% of the strains, 50% indicates that the nucleotide is conserved (identical) in 50% of the strains and 0% indicates that the nucleotide is different in all the strains. The ORFs are color-coded according to their predicted function as in Figure 5. The corresponding sequence identity matrices and sequence difference count matrices calculated in BioEdit from the alignments are shown below each alignment.
