# Supplementary material for: The Mobilome; A Major Contributor to Escherichia coli stx2-Positive O26:H11 Strains Intra-Serotype Diversity
Source: Front Microbiol. 2017 Sep 6;8:1625. doi: 10.3389/fmicb.2017.01625 (PMC5592225; doi:10.3389/fmicb.2017.01625)
Supplement: Supplementary file 17 [file Image8.PDF]

A

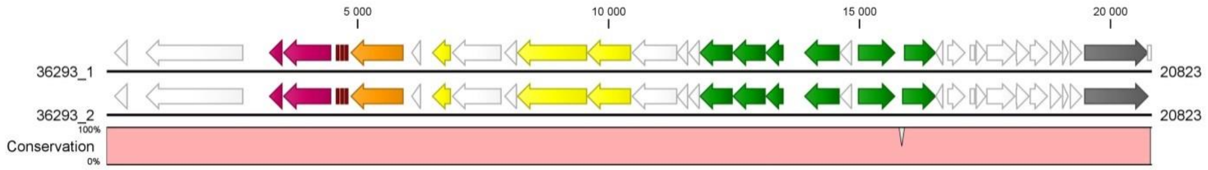

Sequence Identity Matrix

Input Alignment File: 36293-phage1_36293-phage2 alignment.fa

Seq->	36293_ phage1-yecE	36293_ phage2-yciD
36293_ phage1-yecE	ID	0.997
36293_ phage2-yciD	0.997	ID

Sequence Difference Count Matrix

Input Alignment File: 36293-phage1_36293-phage2 alignment.fa

Seq->	36293_ phage1-yecE	36293_ phage2-yciD
36293_ phage1-yecE	ID	62
36293_ phage2-yciD	62	ID

B

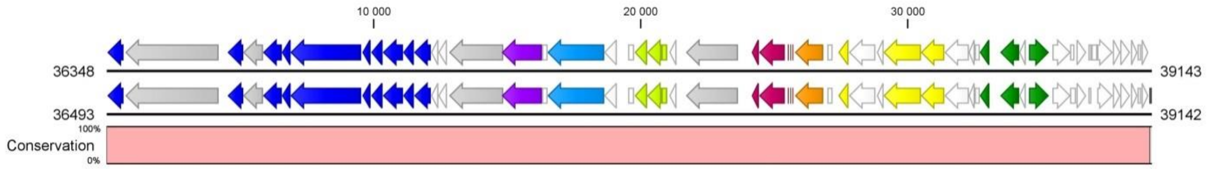

Sequence Identity Matrix

Input Alignment File: 36348_36493 alignment-short.fa

Seq->	36348	36493
36348	ID	0.999
36493	0.999	ID

Sequence Difference Count Matrix

Input Alignment File: 36348_36493 alignment-short.fa

Seq->	36348	36493
36348	ID	31
36493	31	ID

C

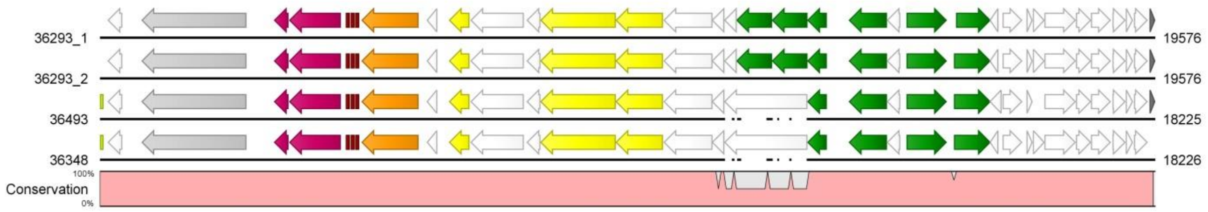

Sequence Identity Matrix
Input Alignment File: All-stx2d-prophages-alignment-short2.1.fa

Seq->	36293_1	36293_2	36493	36348
36293_1	ID	0.997	0.923	0.923
36293_2	0.997	ID	0.926	0.926
36493	0.923	0.926	ID	0.999
36348	0.923	0.926	0.999	ID

Sequence Difference Count Matrix
Input Alignment File: All-stx2d-prophages-alignment-short2.1.fa

Seq->	36293_1	36293_2	36493	36348
36293_1	ID	62	1502	1501
36293_2	62	ID	1444	1443
36493	1502	1444	ID	1
36348	1501	1443	1	ID

Figure S8: Stx2d prophages alignments. A. Comparison of the two stx2d prophages in strain 36293. B. Comparison of the stx2d-prophages in strains 36348 and 36493. C. Comparison of all stx2d prophages over a 20 kb region involved in regulation, replication, nucleotide metabolism and Shiga toxin production. The sequences of the *stx*-prophages were aligned and visualized in CLC Genomics workbench (version 8.0.2). The length of the fragment aligned is indicated on the right. The nucleotide sequence is figured as a black line. Gaps in the alignment are indicated by breaks in the line. Nucleotide identity at each position between fragments is indicated as a plot below the alignment. The height of the line reflects how conserved that particular position is in the alignment. For example, 100% indicates that the nucleotide is conserved (identical) in 100% of the strains, 50% indicates that the nucleotide is conserved (identical) in 50% of the strains and 0% indicates that the nucleotide is different in all the strains. The sequence identity matrix and sequence difference count matrix calculated in BioEdit from the alignment are shown below each alignment. The ORFs are color-coded according to their predicted function as in Figure 5.
