# Supplementary material for: The Mobilome; A Major Contributor to Escherichia coli stx2-Positive O26:H11 Strains Intra-Serotype Diversity
Source: Front Microbiol. 2017 Sep 6;8:1625. doi: 10.3389/fmicb.2017.01625 (PMC5592225; doi:10.3389/fmicb.2017.01625)
Supplement: Supplementary file 18 [file Image9.PDF]

A

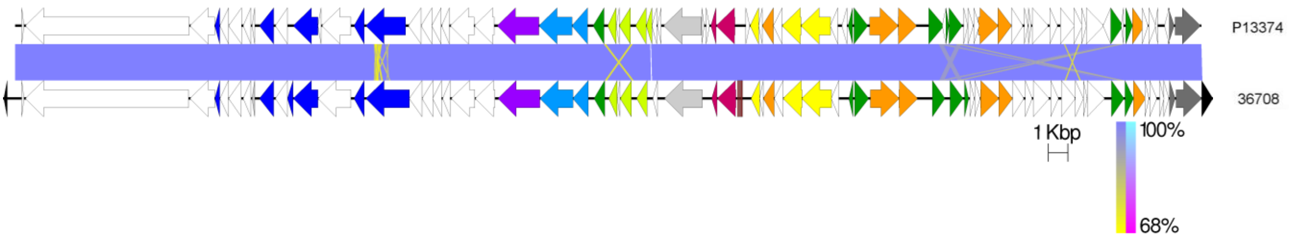

B

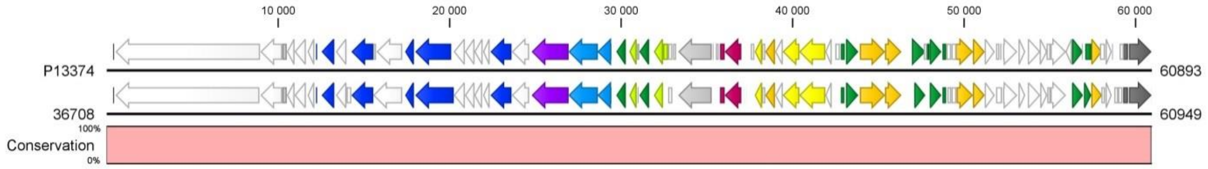

Sequence Identity Matrix
Input Alignment File:P13374-1-36708-alignment.1.fa

Seq->	P13374	36708
P13374	ID	0.997
36708	0.997	ID

Sequence Difference Count Matrix
Input Alignment File: P13374-1-36708-alignment.1.fa

Seq->	P13374	36708
P13374	ID	124
36708	124	ID

Figure S9: **Pairwise comparison of isolate 36708 *Stx2a* prophage with phage P13374 sequence**. **A**. The sequences of the *stx*-prophages were compared with EasyFig. The homologous regions are connected. The color of the zone connecting the strain is related to the direct of reverse homology between the strains, according to the scale present below the comparison. **B**. The sequences of the *stx*-prophages were aligned and visualized in CLC Genomics workbench (version 8.0.2). The length of the fragment aligned is indicated on the right. The nucleotide sequence is figured as a black line. Gaps in the alignment are indicated by breaks in the line. Nucleotide identity at each position between fragments is indicated as a plot below the alignment. The height of the line reflects how conserved that particular position is in the alignment. For example, 100% indicates that the nucleotide is conserved (identical) in 100% of the strains, 50% indicates that the nucleotide is conserved (identical) in 50% of the strains and 0% indicates that the nucleotide is different in all the strains. The sequence identity matrix and sequence difference count matrix calculated in BioEdit from the alignment are shown below each alignment. The ORFs are color-coded according to their predicted function as in Figure 5.
