# Supplementary material for: The Mobilome; A Major Contributor to Escherichia coli stx2-Positive O26:H11 Strains Intra-Serotype Diversity
Source: Front Microbiol. 2017 Sep 6;8:1625. doi: 10.3389/fmicb.2017.01625 (PMC5592225; doi:10.3389/fmicb.2017.01625)
Supplement: Supplementary file 19 [file Image10.PDF]

A

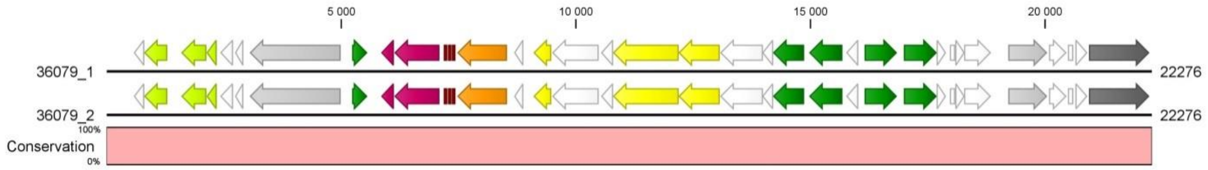

Sequence Identity Matrix
Input Alignment File: 36079_1-36079_2 alignment.fa

Seq->	36079_1	36079_2
36079_1	ID	0.999
36079_2	0.999	ID

Sequence Difference Count Matrix
Input Alignment File: 36079_1-36079_2 alignment.fa

Seq->	36079_1	36079_2
36079_1	ID	1
36079_2	1	ID

B

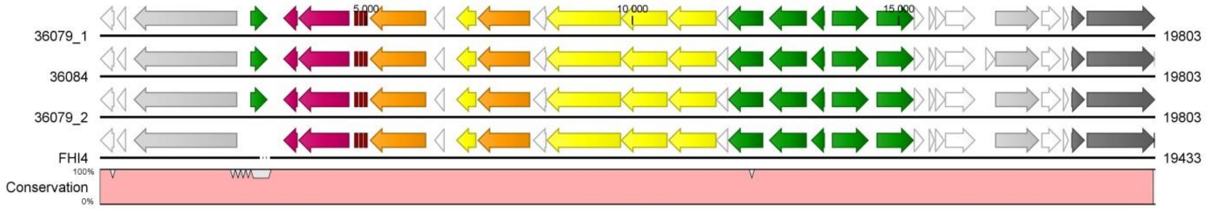

Sequence Identity Matrix
Input Alignment File: stx2a-CC3- alignment-short.fa

Seq->	36079_1	36084	36079_2	FHI4
36079_1	ID	0.999	0.999	0.964
36084	0.999	ID	0.999	0.964
36079_2	0.999	0.999	ID	0.964
FHI4	0.964	0.964	0.964	ID

Sequence Difference Count Matrix

Input Alignment File: stx2a-CC3- alignment-short.fa

Seq->	36079_1	36084	36079_2	FHI4
36079_1	ID	7	1	700
36084	7	ID	6	705
36079_2	1	6	ID	699
FHI4	700	705	699	ID

Figure S10: **SNP-CC3 *Stx2a* prophages alignment. A.** Alignment of strain 36079 *stx2a*-prophages. **B**. Comparison of all SNP-CC3 stx2a prophages over a 19 kb region involved in regulation, replication, nucleotide metabolism and Shiga toxin production. The sequences of the *stx*-prophages were aligned and visualized in CLC Genomics workbench (version 8.0.2). The length of each fragment aligned is indicated on the right. The nucleotide sequence is figured as a black line. Gaps in the alignment are indicated by breaks in the line. Nucleotide identity at each position between fragments is indicated as a plot below the alignment. The height of the line reflects how conserved that particular position is in the alignment. For example, 100% indicates that the nucleotide is conserved (identical) in 100% of the strains, 50% indicates that the nucleotide is conserved (identical) in 50% of the strains and 0% indicates that the nucleotide is different in all the strains. The corresponding sequence identity matrices and sequence difference count matrices calculated in BioEdit from the alignments are shown below each alignment. The ORFs are color-coded according to their predicted function as in Figure 5.
