# Supplementary material for: The Mobilome; A Major Contributor to Escherichia coli stx2-Positive O26:H11 Strains Intra-Serotype Diversity
Source: Front Microbiol. 2017 Sep 6;8:1625. doi: 10.3389/fmicb.2017.01625 (PMC5592225; doi:10.3389/fmicb.2017.01625)
Supplement: Supplementary file 25 [file Image16.PDF]

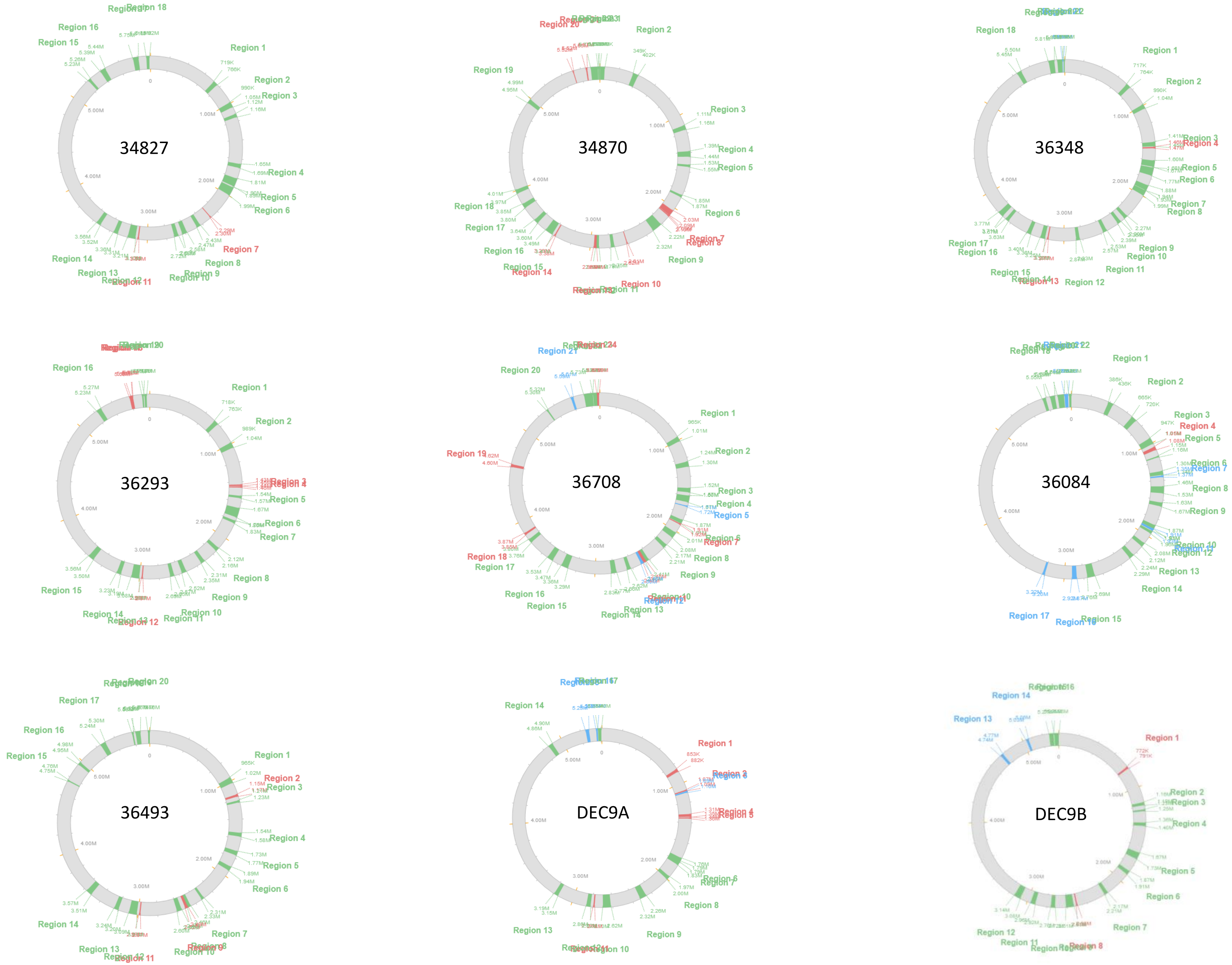
34827
34870
36348
36293
36708
36084
36493
DEC9A
DEC9B

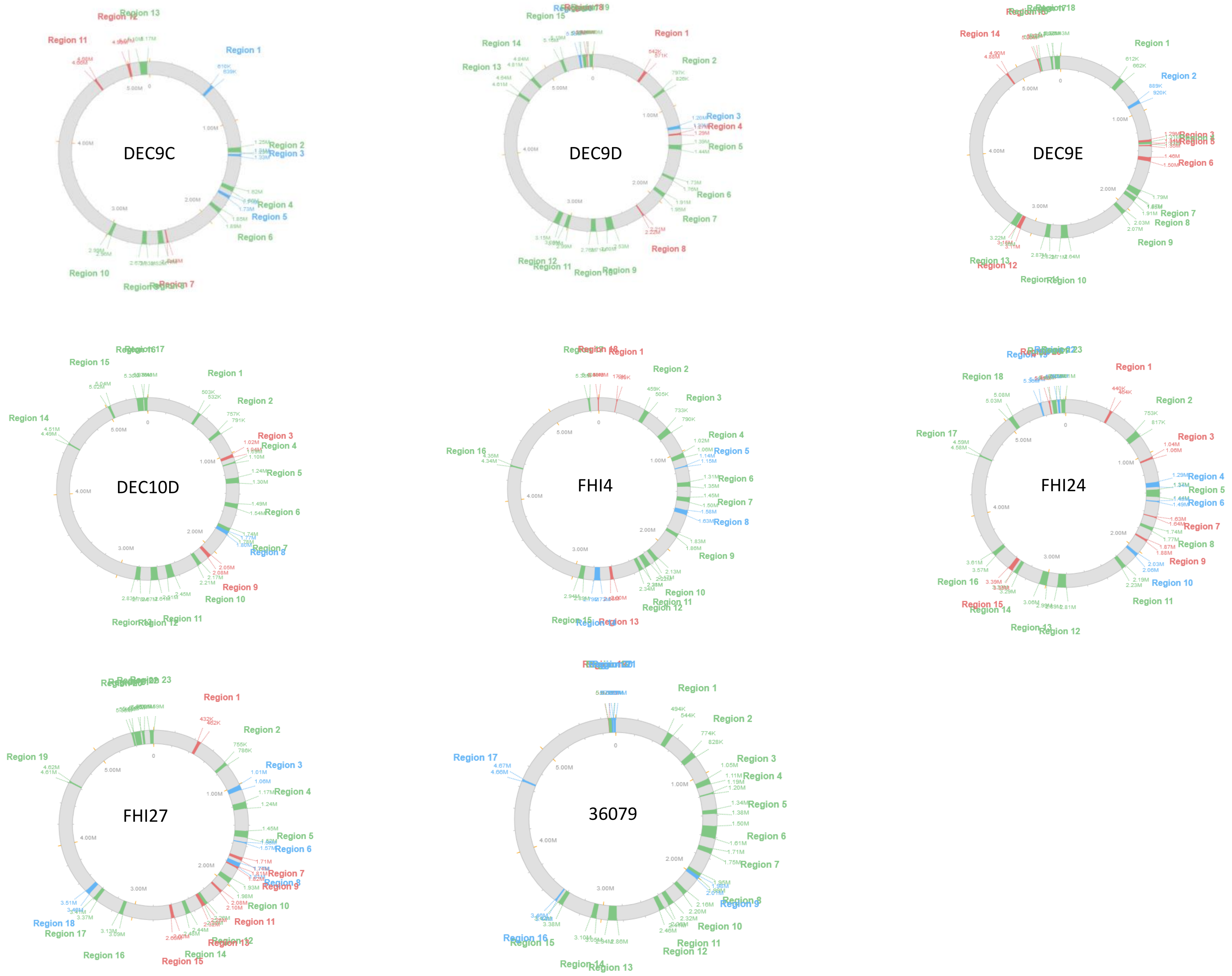
DEC9C
DEC9D
DEC9E
DEC10D
FHI4
FHI24
FHI27
36079

Figure S16: Genomic location of the phage regions. The fasta files generated after Mauve rearrangement to match the genomic arrangement of strain 11368 were concatenated and the prophages were predicted using the PHASTER server. The intact prophages are indicated in green, the questionable prophages are indicated in blue, and the incomplete prophages are indicated in red.
